# SHG-active luminescent thermometers based on chiral cyclometalated dicyanidoiridate(iii) complexes†

**DOI:** 10.1039/d3qi02482b

**Published:** 2024-01-26

**Authors:** Jan Rzepiela, Michal Liberka, Mikolaj Zychowicz, Junhao Wang, Hiroko Tokoro, Kinga Piotrowska, Sebastian Baś, Shin-ichi Ohkoshi, Szymon Chorazy

**Affiliations:** a Faculty of Chemistry, Jagiellonian University Gronostajowa 2 30-387 Kraków Poland simon.chorazy@uj.edu.pl; b Jagiellonian University, Doctoral School of Exact and Natural Sciences Łojasiewicza 11 30-348 Kraków Poland; c Department of Materials Science, Faculty of Pure and Applied Science, University of Tsukuba 1-1-1 Tennodai Tsukuba Ibaraki 305-8573 Japan; d Department of Chemistry, School of Science, The University of Tokyo 7-3-1 Hongo Bunkyo-ku Tokyo 113-0033 Japan

## Abstract

Multifunctional optical materials can be realized by combining stimuli-responsive photoluminescence (PL), *e.g.*, optical thermometry, with non-linear optical (NLO) effects, such as second-harmonic generation (SHG). We report a novel approach towards SHG-active luminescent thermometers achieved by constructing unique iridium(iii) complexes, *cis*-[Ir^III^(CN)_2_(*R*,*R*-pinppy)_2_]^−^ (*R*,*R*-pinppy = (*R*,*R*)-2-phenyl-4,5-pinenopyridine), bearing both a chiral 2-phenylpyridine derivative and cyanido ligands, the latter enabling the formation of a series of molecular materials: (TBA)[Ir^III^(CN)_2_(*R*,*R*-pinppy)_2_]·2MeCN (1) (TBA^+^ = tetrabutylammonium) and (*n*Bu-DABCO)_2_[Ir^III^(CN)_2_(*R*,*R*-pinppy)_2_](i)·MeCN (2) (*n*Bu-DABCO^+^ = 1-(*n*-butyl)-1,4-diazabicyclo-[2.2.2]octan-1-ium) hybrid salts, (TBA)_2_{[La^III^(NO_3_)_3_(H_2_O)_0.5_]_2_[Ir^III^(CN)_2_(*R*,*R*-pinppy)_2_]_2_} (3) square molecules, and {[La^III^(NO_3_)_2_(dmf)_3_][Ir^III^(CN)_2_(*R*,*R*-pinppy)_2_]}·MeCN (4) coordination chains. Thanks to the chiral pinene group, 1–4 crystallize in non-centrosymmetric space groups leading to SHG activity, while the N,C-coordination of ppy-type ligands to Ir(iii) centers generates visible charge-transfer (CT) photoluminescence. The PL characteristics are distinctly temperature-dependent which was utilized in achieving ratiometric optical thermometry below 220 K. The PL phenomena were rationalized by DFT/TD-DFT calculations indicating an MLCT-type of the emission in obtained Ir(iii) complexes with the rich vibronic structure providing a few emission bands that variously depend on temperature due to the role of thermally activated vibrations. As these crucial vibrational modes depend on the crystal lattice, the thermometry performance differs within 1–4 being the most efficient in 4 while the SHG is by far the best also for 4. This proves that pinene-functionalized cyclometalated dicyanidoiridates(iii) are great prerequisites for tunable PL-NLO conjunction with the most effective multifunctionality ensured by the insertion of these anions into bimetallic frameworks.

## Abstract 2

A dicyanidoiridate(iii) complex, bearing chiral (*R*,*R*)-2-phenyl-4,5-pinenopyridine ligands, serves as a SHG-active luminescent thermometer, optimized by embedding into a heterometallic coordination polymer.

## Graphical abstract

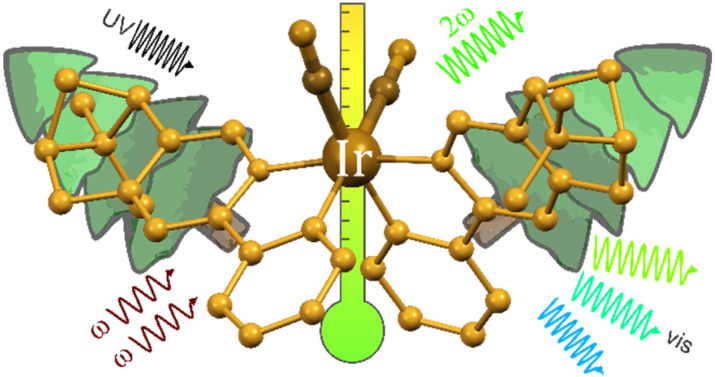

## Introduction

Photo- (PL) and electroluminescent (EL) materials are of great scientific interest due to their wide applications in light-emitting devices (LEDs), photovoltaics, chemical and biochemical sensing, bioimaging, and others.^1–7^ Recent years brought continuous efforts devoted to the design of novel luminescent materials and their unique optical effects, especially those based on metal complexes, including their supramolecular assemblies, coordination polymers (CPs), or metal–organic frameworks (MOFs).^8–10^ An emerging trend is related to the search for luminophores exhibiting strong temperature dependence of emission characteristics, such as band position, lifetime, or ratio between intensities of selected emission peaks.^7,11,12^ Such materials offer contactless and sensitive optical sensing of temperature for medical diagnostics,^13^ chemical reactors,^14^ and nanoelectronics.^15^ Metal complexes, especially those exploring lanthanide ions, and related molecular materials were broadly recognized as an efficient tool for luminescent thermometry operating from cryogenic to high-temperature ranges.^7,16–18^

Non-linear optical (NLO) properties appearing in materials crystallizing in non-centrosymmetric space groups consist of another group of physical effects that are desired due to multiple applications in biological probes/sensors,^19,20^ photo-therapy,^21,22^ imaging,^23,24^ and optics.^25^ The extensive scientific interest is devoted to NLO materials showing second harmonic generation (SHG) or the occurrence of higher harmonics, *e.g.*, the third one (THG).^26–31^ Supramolecular assemblies and CPs/MOFs, exploiting the proper alignment of metal complexes, are efficient in achieving SHG-active crystals.^28–31^

Aiming at extreme miniaturization and optimized efficiency of devices suitable for many applications, the construction of multifunctional materials became an emerging concept.^32,33^ One route of its realization relies on linking optical phenomena, such as the above-mentioned luminescence, including luminescent thermometry, and NLO activity with other physical functionalities, *e.g.*, magnetic or electrical ones.^34–42^ As a result, the unique groups of emissive and SHG-active molecular nanomagnets or ferroelectrics,^34–39^ as well as advanced systems combining luminescent thermometry with nanomagnetism and/or ionic conductivity,^40–42^ were reported. These examples were realized by properly functionalized metal complexes.^32–42^ The alternative pathway toward multifunctionality relies on combining diverse optical phenomena in a single material. For instance, the conjunction of luminescence and chirality results in an emerging class of multifunctional solids, mainly based on metal complexes or organic molecules, exhibiting a circularly polarized luminescence (CPL) with recognized application potential for new-generation LEDs, as well as chiroptical sensors and probes.^43–45^ Recent attention was also devoted to linking NLO properties, such as SHG, with luminescence which was realized using hybrid metal halides or MOFs.^46–49^ For the latter case, the SHG effect could be employed together with up-conversion luminescence (UCL) for optical thermometry.^49^

Our challenge was to develop a novel approach for multi-functional optical materials linking NLO (*e.g.*, SHG) activity and luminescent thermometry that will enable tuning these properties and open the potential to more expanded multi-functionality. Rare previous examples of PL-NLO conjunction focused on the symmetry-breaking toward NLO activity induced by bulky or polar asymmetric organic components (*i.e.*, linkers and/or ions) while the PL-related effects were generated by other molecular building blocks, mainly metal ions and their complexes.^46–49^ On the contrary, we decided to search for versatile chiral metal complexes that will break the crystal's symmetry due to their enantiopure character, ensuring simultaneously both a distinct PL signal and its sensitivity to the temperature. To achieve this, we decided to combine two strategies for molecular luminophores. The first one explores cyclometalated Ir(iii) complexes that contain deprotonated 2-phenylpyridine-type ligands (ppy-L), coordinated to the metal center by N and C atoms. They usually adopt the [Ir^III^(ppy-L)_2_(L′)]^*n*^ (*n* = +1, 0, −1) composition revealing strong visible light PL of a charge transfer (CT) character which can be tuned by an ancillary bidentate L′ ligand. Numerous optical functionalities were achieved for this class of molecules, including efficient EL properties as well as the CPL effect for chiral L′ derivatives.^50–55^ The second strategy relies on using cyanido transition metal complexes which are a great source of stimuli-responsive luminescence, including optical thermometry, usually thanks to the construction of heterometallic coordination frameworks based on inter-metallic cyanido bridges.^56–60^ We and others have found that polycyanidometallates of, *e.g.*, Re(v) or Ru(ii), can be strongly emissive and their PL characteristics are sensitive to external stimuli in related molecular hybrids or CPs/MOFs.^61–65^

**Scheme 1:**
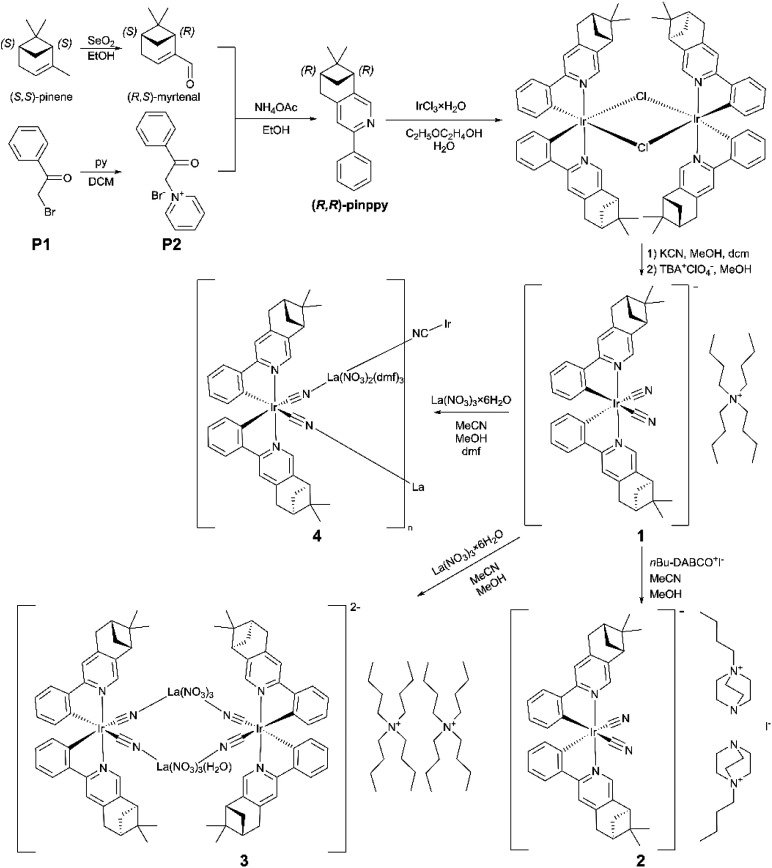
Reported synthetic route for an enantiopure (*R*,*R*)-pinppy ligand starting from (*S*,*S*)-pinene and 2′-bromoacetophenone (P1), and further syntheses of compounds 1–4 which are based on chiral dicyanidoiridate(iii) complexes bearing the deprotonated form of (*R*,*R*)-pinppy. In all cases, the chirality at the ligand is of the (*R*,*R*)-type, whereas the detected diastereoisomers related to the chirality at the metal differ, *i.e.*, the *Δ*-isomers are present for 1 and 2, while the *Λ*-isomers are observed in 3 and 4. They are properly visualized in this scheme (see the text for details).

Following these perspectives, we focused on the synthesis of chiral Ir(iii) complexes bearing both properly functionalized ppy-type and cyanido ligands. To incorporate chirality, we decided to prepare (*R*,*R*)-2-phenyl-4,5-pinenopyridine (*R*,*R*-pinppy) starting from (*S*,*S*)-pinene which is a terpene-type compound, naturally occurring in European pines (Scheme 1).^66,67^ It was found to be an efficient ligand for the construction of chiral metal complexes,^68–73^ including chiral luminescent cyclometalated Ir(iii) and Pt(ii) species.^69–73^ Thus, we used this enantiopure ligand, employing it in the formation of chiral cyanido-cyclometalated Ir(iii) complexes, which were further examined as a source of diverse molecular materials, explored to tune the PL-NLO optical properties. As a result, here, we report the structure and physical properties of chiral [Ir^III^(CN)_2_(*R*,*R*-pinppy)_2_]^−^ complexes incorporated into four different molecular materials, ranging from (TBA)[Ir^III^(CN)_2_(*R*,*R*-pinppy)_2_]·2MeCN (1) (TBA^+^ = tetrabutylammonium) and (*n*Bu-DABCO)_2_[Ir^III^(CN)_2_(*R*,*R*-pinppy)_2_](i)·MeCN (2) (*n*Bu-DABCO^+^ = 1-(*n*-butyl)-1,4-diazabicyclo-[2.2.2]octan-1-ium) hybrid salts, through (TBA)_2_{[La^III^(NO_3_)_3_(H_2_O)_0.5_]_2_[Ir^III^(CN)_2_(*R*,*R*-pinppy)_2_]_2_} (3) square molecules, up to {[La^III^(NO_3_)_2_(dmf)_3_][Ir^III^(CN)_2_(*R*,*R*-pin-ppy)_2_]}·MeCN (4) coordination chains (Scheme 1). These compounds serve as SHG-active luminescent thermometers whose NLO properties and *T*-variable PL patterns originate from the property of chiral Ir(iii) complexes but they are also efficiently tuned by the incorporation of these metal complexes into different supramolecular and coordination assemblies, which was discussed based on experimental data supported by DFT and TD-DFT theoretical calculations.

## Results and discussion

### Synthesis and structural studies

**Fig. 1:**
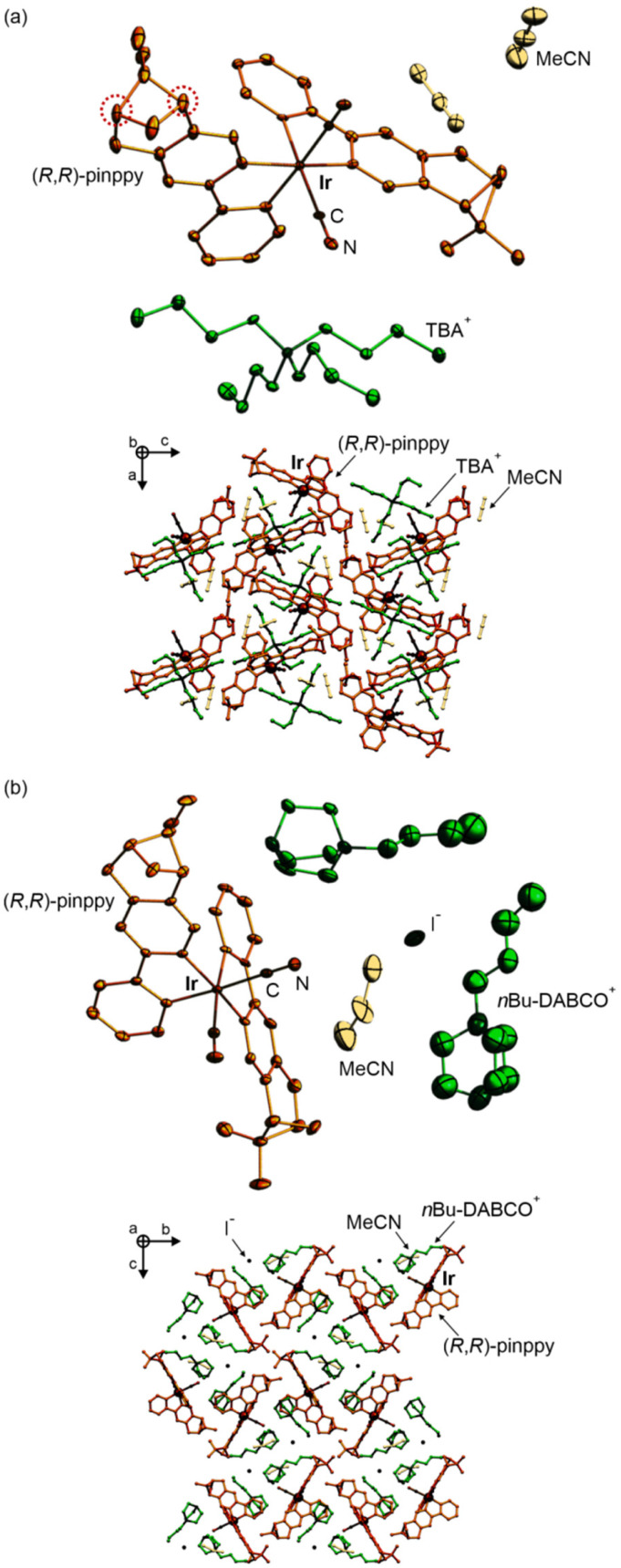
The representative views on the crystal structures of 1 (a) and 2 (b), including the molecular building units (top parts) and the supramolecular arrangements of molecular components (bottom parts). The chiral ligand, heavy atoms, cyanido ligands, solvent molecules, and counter-ions are labeled. The atoms in the top parts were presented as thermal ellipsoids at the 40% and 35% probability levels for 1 and 2, respectively.

At the first stage of the synthetic part, an enantiopure organic ligand (*R*,*R*)-2-phenyl-4,5-pinenopyridine, later referred to as (*R*,*R*)-pinppy, was synthesized by the Kröhnke reaction from α,β-unsaturated aldehyde (*R*,*S*)-myrtenal (obtained from an (*S*,*S*)-pinene terpene precursor) and appropriate pyridinium salt (Scheme 1, see the ESI† for details).^67,74^ The (*R*,*R*)-pinppy ligand was comprehensively characterized by NMR and IR spectra, as well as a single-crystal X-ray diffraction (SC-XRD) method (Fig. S1 and S3, Table S1†). Next, a three-step synthetic procedure was applied to obtain an enantiopure (*R*,*R*)-pinppy-functionalized dicyanidoiridate(iii) anionic complex which was done by modifying and optimizing the methods described for analogous non-chiral Ir(iii) complexes (Scheme 1, see the ESI† for details).^75^ The tetrabutylammonium (TBA^+^) salt of the formula of TBA[Ir^III^(CN)_2_(*R*,*R*-pinppy)_2_]· *n*(solvent) (1) was primarily obtained as an amorphous solid but further recrystallized by the slow diffusion of diethyl ether into the related acetonitrile solution. It gave the crystalline phase with the MeCN as a solvent which was sufficient to be examined by the SC-XRD method performed together with the set of spectroscopic and TG characterization (Fig. S1 and S2†). 1 crystallizes in the orthorhombic *P*2_1_2_1_2_1_ space group as a molecular hybrid salt consisting of an octahedral *cis*-[Ir^III^(CN)_2_{*trans*-(*R*,*R*-pinppy)_2_}]^−^ anion (*cis*-configuration for cyanido ligands, and *trans*-configuration for pyridine groups of pinppy ligands, these features remain in all compounds), an aliphatic TBA^+^ cation, and weakly bonded MeCN molecules, two per one Ir^III^ complex (Fig. 1a and S4, Tables S1, S2 and S6†).

**Fig. 2:**
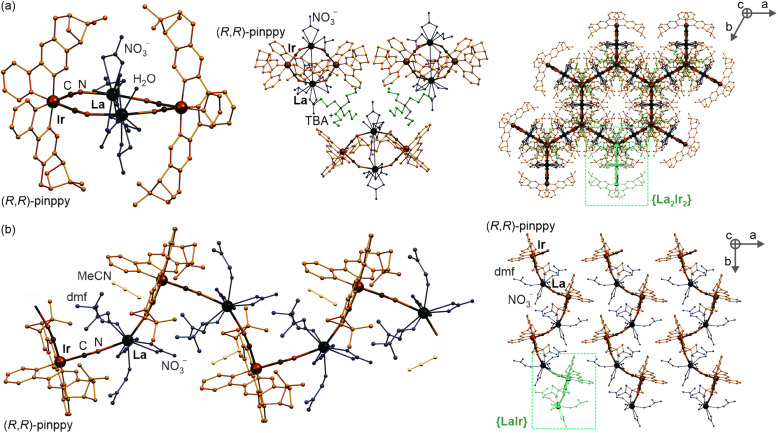
The representative views on the crystal structures of 3 (a), including the tetranuclear molecule (left part, without TBA^+^ cations that were omitted for clarity), the alignment of these molecules and organic counter-ions (middle part), and the broader view on the supramolecular framework along the six-fold axis, on which one tetranuclear molecule is highlighted in light green (right part), and the view on the crystal structure of 4 (b), including the fragment of a coordination polymer together with solvent molecules of crystallization (left part) and the arrangement of these coordination chains, on which the dinuclear molecular unit is highlighted in light green (right part). The chiral ligand, heavy atoms, cyanido ligands, solvent molecules, and counter-ions are labeled.

Compound 1 was further used as a precursor for the synthesis of three different chiral molecular materials of 2–4 (Scheme 1). For the case of 2, a polar cation, namely *n*Bu-DABCO^+^, in the form of an iodide salt was introduced to the solution of 1, resulting in the formation of a multi-component (*n*Bu-DABCO)_2_[Ir^III^(CN)_2_(*R*,*R*-pinppy)_2_](i)·MeCN (2) molecular hybrid. It also crystallizes in the orthorhombic *P*2_1_2_1_2_1_ space group, consisting of the chiral octahedral Ir^III^ complex, preserved from the structure of 1, but accompanied by two *n*Bu-DABCO^+^ cations, one MeCN molecule, and a single iodide ion ensuring the overall neutrality of the material (Fig. 1b, S2, S3 and S5, Tables S1 and S6†). To check the coordination ability of the obtained Ir^III^ anionic complex, 1 was combined with La^III^(NO_3_)_3_·6H_2_O in the MeCN/MeOH solution which was followed by slow diffusion of diethyl ether resulting in (TBA)_2_ {[La^III^(NO_3_)_3_(H_2_O)_0.5_]_2_[Ir^III^(CN)_2_(*R*,*R*-pinppy)_2_]_2_} (3) coordination assembly. It crystallizes in the different hexagonal *P*6_2_22 space group and is composed of {La^III^_2_Ir^III^_2_}^2−^ cyanido-bridged squares, incorporating unchanged chiral Ir^III^ complexes but linked to La^III^ centers bearing also nitrate anions and aqua ligands. The additional La–La bridging is ensured by one of the present nitrate anions. These tetranuclear molecular anions are accompanied with TBA^+^ counter-ions (Fig. 2a, S1, S2 and S6, Tables S1, S4 and S6†). To modulate the structure of a bimetallic La^III^–Ir^III^ material, a small amount of dimethylformamide (dmf), revealing a strong affinity to coordinate to lanthanide ions, was added to the solution of 1 and La^III^(NO_3_)_3_·6H_2_O. This results in {[La^III^(NO_3_)_2_(dmf)_3_][Ir^III^(CN)_2_(*R*,*R*-pinppy)_2_]}·MeCN (4) cyanido-bridged chains crystallizing in the orthorhombic *P*2_1_2_1_2_1_ space group, identical to 1 and 2 (Fig. 2b, S1, S2 and S7, Tables S1, S5 and S6†). These chains exhibit a zig-zag shape which is due to the presence of an almost ideally octahedral Ir^III^ metalloligand bearing two bridging CN^−^ ligands that are in the *cis*-configuration. The La^III^ complexes are 9-coordinated of a rarely observed muffin geometry realized by two CN^−^, three dmf, and two bidentate nitrate ligands. The latter ensures the neutrality of the material, thus it crystallizes only with weakly bonded MeCN molecules.

It is important to note here that bis-cyclometalated Ir(iii) complexes, [Ir^III^(CN)_2_(*R*,*R*-pinppy)_2_]_2_, detected in the crystal structures of all reported compounds, 1–4, exhibit not only chirality on the ligand (*i.e.*, *R*,*R*-pinppy in all investigated compounds) but also at the metal (*e.g.*, *Δ* and *Λ* isomers). In the presented structural models of 1–4, which are determined by the SC-XRD method, a single-type diastereoisomer was found. The detected diastereoisomers for each compound are visualized in Scheme 1; in 1 and 2, the *Δ*-isomers were found, while, in 3 and 4, the *Λ*-isomers are observed; however, this was found for selected single crystals, thus this result does not prove that only one diastereoisomer is present in the respective bulk samples. The P-XRD data (discussed below) for the powder sample cannot also help in the discrimination between two different diastereoisomers. Most probably, the mixture of crystals with two opposite diastereoisomers is observed for all compounds. This suggestion is supported by the performed ^1^H NMR studies on the dissolved sample of 1 which reveals that the mixture of diastereoisomers (*RRΛ* and *RRΔ*) is present in the powder sample of 1 (see Experimental details in the ESI†). As this compound was further used to prepare the other materials, 2–4, it can be concluded that all obtained materials presumably contain the mixture of crystals of two different diastereoisomers. We do not focus on this issue here as the investigated optical properties (see below), including SHG, PL, and optical thermometry, are not chiroptical ones, thus they are expected to be identical for both diastereoisomers. For eventual future work, *e.g.*, related to the CPL activity, the purification of the precursor Ir(iii) complexes towards a single diastereoisomer will be the critical issue. Such work is planned in our future work but it is far beyond the scope of the presented work.

The physicochemical characterization of 1–4, including CHN elemental analysis, IR spectra, and TGA indicate that these materials are hygroscopic, and exposition to air results in their hydrated forms (see the ESI† for details); however, it does not lead to significant structural transformation as was confirmed by the powder X-ray diffraction (P-XRD) analysis showing the phase purity and the identity of the patterns for the air-dried powder sample and the calculated one from the structural model obtained in the SC-XRD analysis (Fig. S8†). Moreover, the process of gradual water adsorption was found to occur slowly, within a couple of days; thus, for accurate and consistent experimental data, all further physical measurements were performed on freshly filtered samples.

### Second-harmonic generation activity

**Fig. 3:**
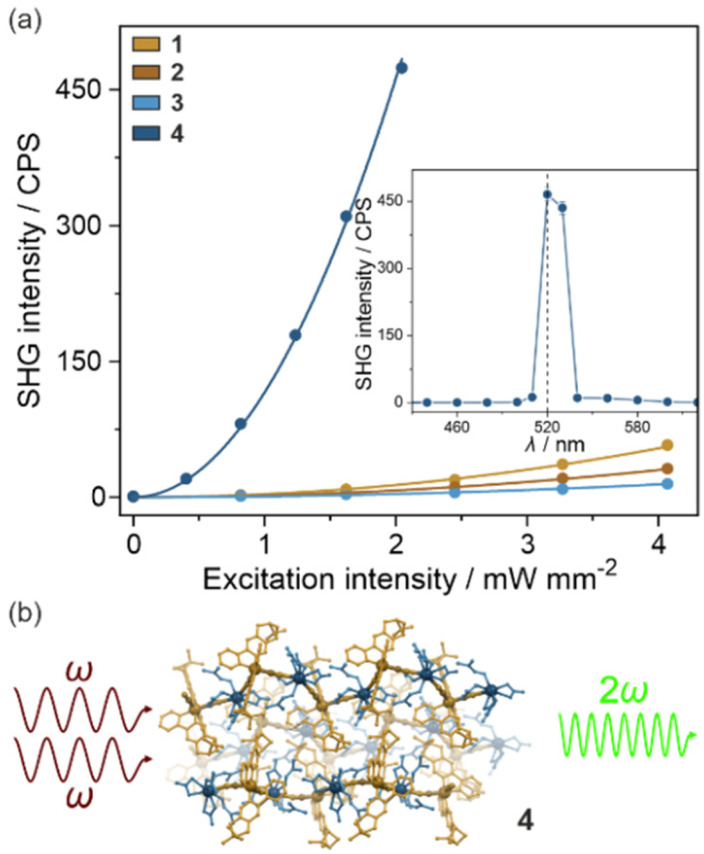
Room-temperature SH light intensity for 1–4 as a function of excitation intensity (a) shown together with the dependence of the SH signal upon wavelength (the inset). The filled points represent experimental data while solid lines (for the SHG *versus* excitation intensity characteristics) indicate the best-fit curves (a quadratic equation). The solid line in the inset is only to guide the eye. The (b) part contains the schematic illustration of the SHG effect generated in the structure of 4.

When an enantiopure ligand is present in the crystal structure, the number of possible space groups is limited to the 65 Sohncke group and all of them are non-centrosymmetric enabling the observation of the second harmonic generation (SHG) effect. Therefore, thanks to the (*R*,*R*)-pinppy ligand coordinated to Ir(iii) centers (Fig. 1 and 2), the whole series of obtained compounds, 1–4, were tested for SHG activity. This effect is related to the interaction of two photons at the *ω* frequency with the crystalline sample without an inversion center that leads to the emission of a photon with double frequency (2*ω*). To examine this effect for powder samples of 1–4, a homemade experimental setup was used, which was described previously.^38^ In principle, the samples were placed between two glass plates and irradiated with a near-infrared pulse laser (1040 nm). Consequently, the intensity of visible light emitted by the sample was measured both in the function of wavelength as well as laser power (Fig. 3, S9, and S10†). For all compounds, this emitted light reveals a sharp peak at 520 nm and its intensity is proportional to the square of the laser power, both observations indicating that the two-photon SHG process is observed. To further quantify the quality of the SHG activity in the obtained materials, the reference sample of commercially used potassium dihydrogen phosphate (KDP) was measured under identical experimental conditions, and the resulting signal was compared with those for 1–4 (Fig. S9 and S10†). It was found that the intensities of the emitted SH light in 1–3 were small, not exceeding 0.2% of KDP, reaching 0.17% (±0.01%) of KDP for 1, 0.096% (±0.006%) of KDP for 2, and 0.045% (±0.005%) of KDP for 3. On the other hand, compound 4 reveals a relatively strong SHG effect, nearly thirty times stronger than 1–3, as the related SH light was found to reach 5.8% (±0.2%) of KDP. This indicates the positive role of the incorporation of chiral Ir(iii) complexes into heterometallic coordination polymer, especially since both 1, 2, and 4 possess identical space groups (*P*2_1_2_1_2_1_) but the hybrid salts of 1 and 2 reveal much weaker SHG activity.

Following these findings, one could have expected a relatively higher SHG activity for 3 which is composed of coordination squares with similar cyanido bridges as 4; however, it crystallizes in a very different *P*6_2_22 space group. In principle, according to the Kleinman rule, in non-dispersive materials, the SHG effect should not be observed in the space groups representing the series of 422, 432, and 622 point groups.^76,77^ Thus, it is not surprising that the SHG activity for 3 is the weakest along the obtained series of compounds; however, we have repeatedly obtained the non-negligible SHG activity for this compound which did not come from the impurity as the purity of this material was precisely checked (Fig. S8†). This non-zero SHG activity appeared despite the 622 point group which agrees with the other reports on similarly crystallizing molecular materials and inorganic solids that were shown to break the Kleimann symmetry rule.^78–80^

### Light absorption and photoluminescence studies

**Fig. 4:**
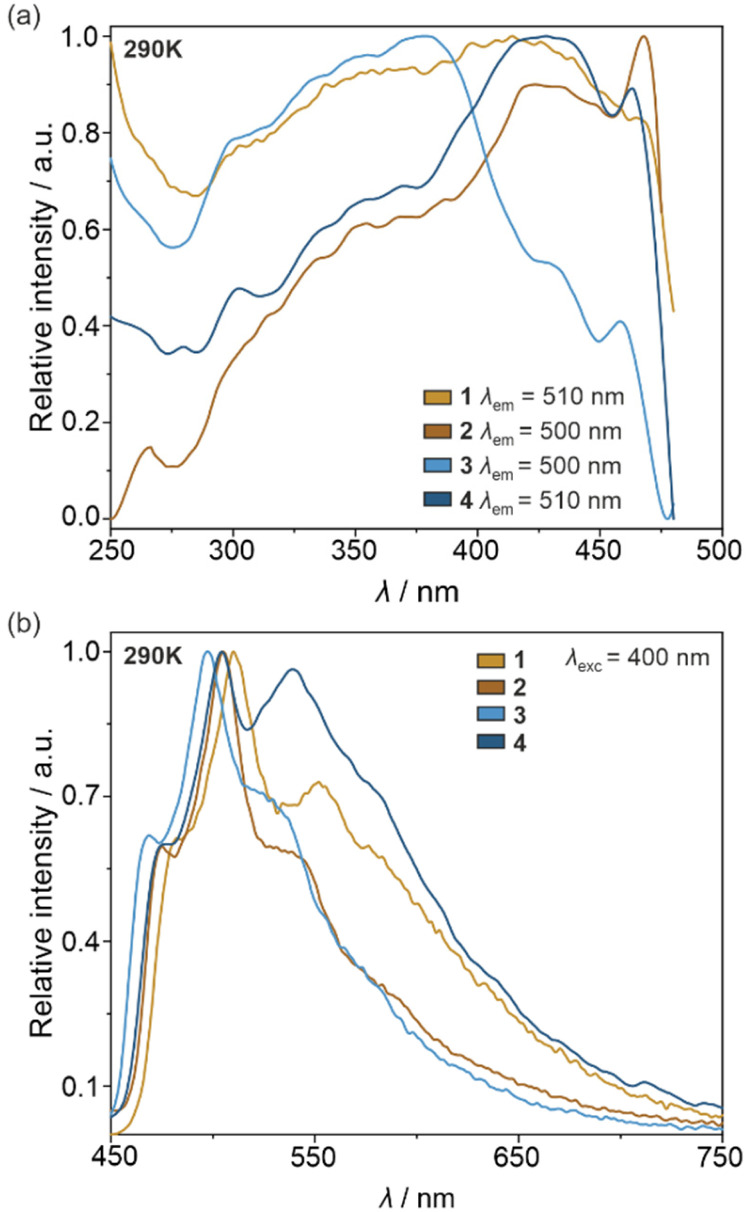
Solid-state photoluminescence characteristics of 1–4 at 290 K, including the set of respective excitation spectra for indicated monitored emission wavelengths (a) and the corresponding emission spectra for the indicated excitation wavelength (b).

Under daylight, the physical color of the powder samples of all obtained molecular materials is yellow. Simultaneously, they contain the cyclometalated Ir(iii) complexes, known for their efficient vis-to-NIR photoluminescence.^50–55,75^ Therefore, 1–4 together with the crystalline form of (*R*,*R*)-pinppy were investigated by solid-state UV-vis-NIR absorption (Fig. S11†) as well as photoluminescence spectra (Fig. 4 and S12–S17†), the latter discussed using also the analysis of emission colors on the CIE 1931 chromaticity scale (Tables S7–S11 and Fig. S18†). All obtained solids show strong absorption in the 220–340 nm region related to the π → π* electronic transitions within the (*R*,*R*)-pinppy ligands and, for 1–4, charge transfer (CT) electronic transitions within the [Ir^III^(CN)_2_(*R*,*R*-pinppy)_2_]^−^ complexes. The eventual additional absorption related to the d–d electronic transitions of Ir(iii) centers is expected to be much weaker, thus without significant contribution to the observed absorption bands. Moreover, 1–4 exhibit a broad absorption band in the visible part of the spectrum ranging from the UV region edge to *ca.* 440–460 nm, which is also associated with the CT electronic transitions, mainly of a metal-to-ligand charge transfer (MLCT) type, within the Ir(iii) complexes as proven by TD-DFT calculations (see below). For 3 and 4, this MLCT band is shifted to the higher energies, which can be assigned to the coordination of the crucial Ir(iii) complexes to the heavy La(iii) centers. Following strong UV and visible light absorption limiting to *ca.* 460 nm, the visible-to-NIR photoluminescence of obtained solids was investigated. The pure (*R*,*R*)-pinppy molecules do not show any luminescent properties at room temperature; however, when cooled to 77 K and excited with the 435 nm light, they emit yellowish-green light with a maximum positioned at 510 nm (Fig. S12†). The photoluminescence under various wavelengths of the UV-to-vis light excitation (at least from 250 to 460 nm) was found for compounds 1–4 (Fig. 4). The related emission bands are broad and situated in the similar 450–660 nm range as found for (*R*,*R*)-pinppy ligand; however, contrary to a pure ligand, the emission of 1–4 is easily detectable at room temperature, and three distinguishable emission components, with the maxima separated by approximately 1200–1400 cm^−1^, are observed (Fig. 4b). For instance, for 1 at 290 K, they are detected at 482.5, 510, and 552 nm. The enhancement of emission going from organic (*R*,*R*)-pinppy to 1–4 bearing Ir(iii) complexes with (*R*,*R*)-pinppy, together with the appearance of structured emission patterns, indicates that the luminescence of 1–4 can be assigned to the charge transfer electronic transitions which, for a family of cyclometalated Ir(iii) complexes, typically originates from the MLCT excited state.^50–55,75^ This emission is often structured due to the coupling with vibrational modes, typically with aromatic C–C bond stretching vibrations appearing in the 1200–1400 cm^−1^ energy range. This seems to be also the case of 1–4 as such the energy splitting of the emission is observed and the properly lying absorption bands in the IR spectra are also present (Fig. 4b and S1†). We further confirmed the assignment of the observed emission in 1–4 to the MLCT character using TD-DFT calculations (see below).

As a result, all obtained molecular materials exhibit strong luminescence at room temperature and below (Fig. 4 and S18, Tables S7–S11†). At 290 K, this luminescence ranges from yellowish green for 1 and 4, green for 2, up to blue-green for 3 (Fig. S18†). The related excitation spectra contain numerous broad bands ranging from 250 to *ca.* 480 nm, and they are qualitatively similar along the series of 1–4; however, compounds 2 and 4 reveal much weaker excitation bands in the deeper UV range. The differences within the 1–4 family are also noticeable in the emission patterns. What can be easily noticed is the energy position of a global emission maximum, which is shifted gradually towards higher energies within the sequence of 1–2–4–3 compounds. This effect can be associated with the gradual structural modification of Ir(iii) complexes due to the appearance of heavy atoms, *i.e.*, iodide ions interacting non-covalently with Ir(iii) complex in 2 and La(iii) centers bridged through cyanido ligands to Ir(iii) centers in 3 and 4 (Fig. 1 and 2). Moreover, an additional energy shift of the emission maximum can be seen between the last two compounds; the blue shift is observed going from 4 to 3, which can be correlated with the further structural changes occurring for the Ir(iii) complex in the molecular squares of 3. This is connected with additional intramolecular nitrate bridges between La(iii) centers decreasing the distance between these metal centers, and further enforcing the smaller NC–Ir–CN angle within the Ir(iii) complex (approaching, in 3, almost 90 degrees giving a nearly perfect octahedron, Table S6†) than in the simpler coordination polymer bearing only cyanido bridges in 4. The analogous energy shifts can be observed for other emission bands, *e.g.*, those positioned at higher energies around 470 nm. Together with the energy shifts of the emission pattern, compounds 1–4 reveal variable room-temperature absolute emission quantum yields (QYs), ranging from 11(3)% for 3, through 18(3)% for 4, up to 20(3)% for 1 and 22(3)% for 2. The noticeably smaller value for 3 can be, as above, correlated with the significant deformation of Ir(iii) complexes within molecular squares of 3 as well as the large structural disorder in this compound and the presence of emission-quenching water molecules within the Ir(iii)-complex-containing tetranuclear squares (Fig. 2a). Interestingly, the emission lifetime for the respective main emission maxima adopt almost the opposite trend, ranging from 0.54(1) μs for 1, 0.95(2) μs for 2, 1.5(1) μs for 4, up to 2.5(2) μs for 3 (Fig. S19–S22, Tables S12 and S13†).

Another significant difference between emission spectra of 1–4 is related to the relative intensities of three main components detectable at 290 K (Fig. 4b). This observation could not be straightforwardly correlated with the structural features of respective compounds as the related trends do not follow those in energy shifts or QYs; however, the variation in the relative intensities of emission components suggest that the related electronic transitions and their coupling with vibrational modes of organic ligands are very sensitive to the coordination and supramolecular environment, and this correlation differs depending on the selected component of the emission pattern. This will be discussed more in the DFT and TD-DFT calculations section after getting a better insight into the nature of the emissive electronic transitions (see below). Nevertheless, one could expect that external stimuli, such as temperature, will be also able to affect the emission components in a distinguishable way. We tested this by gathering the set of temperature-dependent luminescence characteristics for 1–4, uncovering the strong influence of temperature on photoluminescence, and opening the pathway for the construction of luminescent thermometers which will be described in the next section.

### Luminescent thermometry

**Fig. 5:**
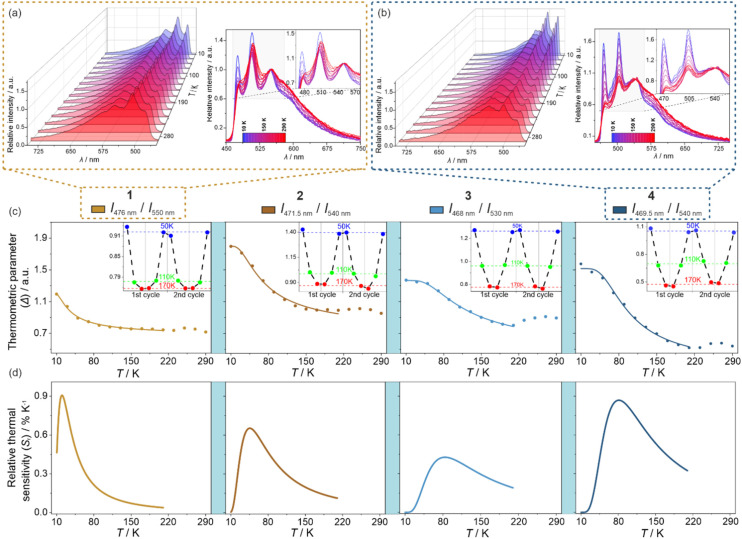
Luminescent thermometry characteristics of 1–4, including the sets of temperature-variable normalized emission spectra under the 400 nm excitation for 1 (a) and 4 (b), presented by a 3-D plot as well as by overlapped spectra at indicated temperatures (the comparison of data for all compounds is provided in Fig. S13–S16†), the temperature dependences of selected thermometric parameters (c) (defined as the ratios between indicated emission wavelengths), shown with the repeatability cycles of thermometric behavior (the insets), and the resulting relative thermal sensitivity curves (d). In (c), filled points are experimental data while solid lines represent the best-fit curves following eqn (1).

Both excitation and emission spectra of the freshly prepared samples of 1–4 were gathered under variable temperatures from the 10–290 K range. The obtained results, which are presented in Fig. 5, S13–S18, and Tables S7–S11,† indicate the strong impact of temperature on luminescent features. Upon cooling, there are noticeable, yet not dramatic, energy shifts of the emission maxima, generally of the redshift character. The tiny thermally-induced energy shifts are also visible in the excitation spectra, which, however, reveal rather small changes in the shape upon cooling. On the contrary, there are significant cooling-induced changes in the detailed emission spectra. To emphasize the thermal changes in photoluminescence, the temperature-variable emission spectra of 1–4 were normalized to the emission component lying in the 530–570 nm range. After this procedure, it is nicely visible that, upon cooling, the two higher-energy emission components increase their intensity much stronger than the normalization peak (Fig. 5a, b and S13–S16†). As a result, the ratio between the intensities of the respective peaks, one of the peaks below 520 nm and the peak lying in the 530–570 nm region, can be used as the thermometric parameter, *Δ*, which is the basis of the application of 1–4 as ratiometric optical thermometers.^7,11–18^ The most pronounced thermometric effect of the described type was detected in 4. Therefore, this compound was used as a model to optimize the thermometric parameter by testing various possible combinations of the emission intensities, including the intensities of the exact maxima slightly shifting with temperature, the intensities at the specific wavelengths, the areas below the indicated peaks, *etc*. (see Fig. S16 with the comment in the ESI†). From this analysis, it was found that the best-performance optical thermometry is achieved using the ratio between the emission intensities at wavelengths at which the maxima of two peaks, the first from the 460–480 range and the second from the 530–570 nm range, appear at the lowest measured temperature (10 K). Thus, for 4, the optimized thermometric parameter was defined as the ratio between the emission intensities at 469.5 and 540 nm (Fig. 5b, c and S16†). The thermometric parameters for other compounds, 1–3, were defined analogously, thus they slightly differ as given in Fig. 5c and S17.† In all cases, the thermometric parameter was found to be strongly temperature-dependent as presented in Fig. 5c, which is the representation of a ratiometric luminescent thermometric effect. In the range of largest thermal changes appearing below 220 K, the *Δ*(*T*) curves could be fitted to a classical Mott-Seitz model taking into account a single non-radiative relaxation channel governing the thermal emission changes in obtained compounds. It is represented by eqn (1):1
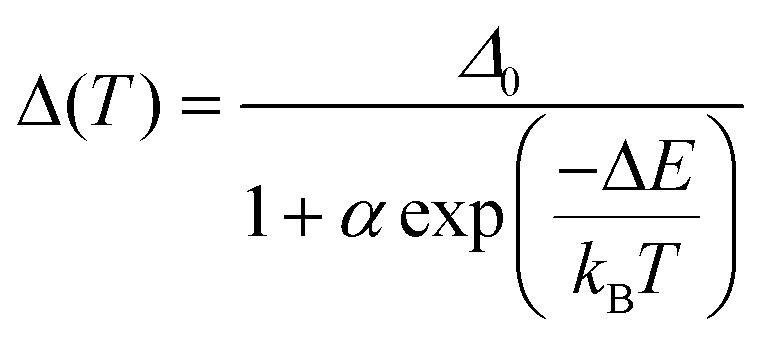
where *Δ*_0_ is a thermometric parameter at 0 K, *α* is the ratio between non-radiative and radiative rates, and Δ*E* is the activation energy of a non-radiative channel. The best-fit curves are presented in Fig. 5c, S16 and S17,† while the resulting best-fit parameters are given in Table S11.† They were used to obtain the relative thermal sensitivity curves, *S*_r_(*T*), and the related temperature uncertainty (Fig. 5d, see also Fig. S17 and the related comment in the ESI† for details). The relative thermal sensitivity does not exceed 1% K^−1^ indicating that the optical thermometric effect is rather moderate within the whole series; however, the reasonable limit of 0.5% K^−1^ is achieved for certain temperature ranges in all compounds, except 3. The highest *S*_r_ value, 0.91% K^−1^ at 20 K, was found for 1 but the operating range of the related thermometry (within the reasonable limit of *S*_r_) appears only in the very low-temperature range below 50 K, while 4 shows similarly high maximal *S*_r_ value, 0.87% K^−1^ at 81 K but it accompanied by the broader 40–150 K range of *S*_r_ > 0.5% K^−1^ (Fig. 5d). This indicates that the thermometric effect related to the intrinsic thermal variation of cyclometalated Ir(iii) complexes is the most pronounced after their incorporation into heterometallic coordination polymers of 4. The observed optical thermometric effect was checked in a few consecutive cycles of heating and cooling (Fig. 5c). These experiments prove the good repeatability of the constructed ratiometric thermometers. Besides the ratiometric approach towards thermometry, we also tested the possible application of emission lifetime as a thermometric parameter; however, it shows much weaker thermal changes than those described for emission intensities ratios (Fig. S19–S22 and Table S13†). Representative parameters of all presented optical properties of 1–4, including SHG activity as well as photoluminescence, and the related ratiometric optical thermometry, were gathered in Table 1.

**Table 1:** Comparison of main parameters illustrating SHG activity, photoluminescence, and optical thermometry found in compounds 1–4. The italicised values of emission maxima correspond to the main maximum within the respective emission pattern

Compound	SHG intensity (Fig. 3)/% KDP	Emission maxima, *λ*_em,max_ at 290 K (Fig. 4)/nm	Emission quantum yield, QY/%	Max. *S*_r_ of a thermometric effect (Fig. 5)/% K^−1^
1	0.17(1)	482.5, *510*, 552	20(3)	0.91 (at 20 K)
2	0.096(6)	475, *505*, 538.5	22(3)	0.65 (at 45 K)
3	0.045(5)	469, *497.5*, 529	11(3)	0.43 (at 84 K)
4	5.8(2)	473, *503.5*, 537.5	18(3)	0.87 (at 81 K)

### DFT and TD-DFT calculations

To elucidate the observed optical phenomena in the discussed series of compounds, including both absorption as well as temperature-variable emission, we employed the density-functional theory (DFT) and its time-dependent variant (TD-DFT). The calculations were conducted using only the anionic [Ir^III^(CN)_2_(*R*,*R*-pinppy)_2_]^−^ moiety, with the initial set of atomic positions derived from the crystal structure of 1.

**Fig. 6:**
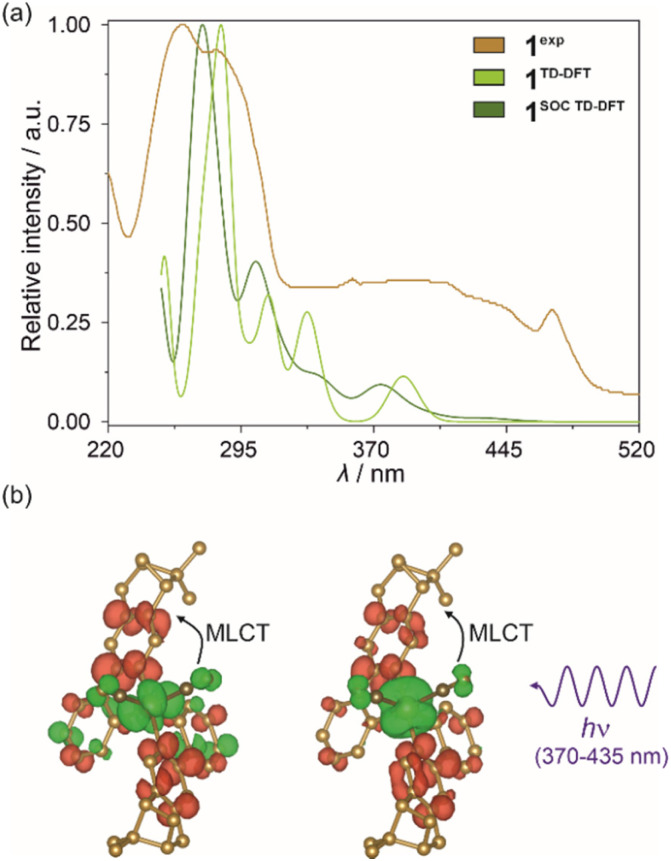
Room-temperature solid-state UV-vis absorption spectrum of 1 (1^exp^) compared with the spectra obtained from TD-DFT calculations (1^TD-DFT^) and SOC-corrected TD-DFT (1^SOC TD-DFT^) calculations performed for *cis*-[Ir^III^(CN)_2_(*R*,*R*-pinppy)_2_]^−^ units (a) (see the text, Fig. S24 and S25, Tables S15 and S16†), and differential electron density maps for two low-lying SO-states for the ground state geometry of the mentioned Ir(iii) complex (b), which represent the changes in electron density (positive changes shown in red, negative changes shown in green) upon optical transitions responsible for the absorption of light from the 370–435 nm range (the calculated lower-energy part of absorption bands of 1, see Table S16 and Fig. S25† for details). The changes in electron density illustrating a dominant metal-to-ligand charge transfer (MLCT) character of electronic transitions were indicated. The densities were plotted with an isosurface level of 0.0016.

The DFT calculations were performed according to the steps thoroughly described in the Computational details in the ESI† using the ORCA 5.0.1 quantum chemistry program package.^81^ The geometry of the selected structural unit was optimized for the ground state and later also for the first excited state. The resulting structural models were found to be similar to those found from SC-XRD (Fig. S23 and Table S14†). However, minor differences were noticed which could be the reason for further differences between the experimental and simulated optical spectra. Density maps of molecular TD-DFT orbitals for a few HOMO and LUMO states were obtained for the optimized ground state. Nevertheless, they do not reveal any useful information on the critical electronic transitions due to the extensive delocalization of electron density on the whole molecule (Fig. S24†). Additionally, the related singlet and triplet states responsible for light absorption are complex mixtures of these molecular orbitals (Table S15†). Therefore, to take into account the presence of heavy elements, which is expected to be crucial for the validity of the DFT approach in the case of Ir(iii) complexes, and to improve the correlation between the theoretical and experimental data, the TD-DFT calculations were further corrected for SOC (spin–orbit coupling). This resulted in the set of SO-states having now mixed single-triplet characters (Table S16†). Based on such states, electronic dipole transition intensities were calculated to recreate the experimental UV-vis absorption spectrum of 1 (Fig. 6a). Incorporating spin–orbit coupling in the theoretical treatment resulted in a shift of the states to slightly higher energies enabling the overall better match with the experiment. Then, to understand the nature of electronic transitions responsible for the observed light absorption in the UV-vis range, the related differential electron density maps were calculated for the lowest-lying SO-states. They represent the differences between linear combinations of molecular orbitals before and after the absorption of light of the indicated energy. Such maps, for the two lowest-lying SO-states, are shown in Fig. 6b while the broader set is provided in Fig. S25.† The red parts illustrate the gain in electron density, and the green parts the loss in electron density after transition. For all of the lowest-lying SO-states, it can be seen that a negative change in electron density is placed mainly on Ir(iii) center, only partially on cyanido ligands, and the parts of the (*R*,*R*)-pinppy ligands which are close to the metal center. Moreover, the resemblance of the regions with an outflow of electron density to the shape of d-orbitals is clear. On the other hand, the positive change in electron density is localized exclusively on both (*R*,*R*)-pinppy ligands, mainly on their aromatic rings. Therefore, according to this analysis, the observed light absorption in 1 could be described as a metal-to-ligand charge transfer (MLCT) transition with the partial assistance of cyanido ligands, and a small admixture of (*R*,*R*)-pinppy intraligand electronic transitions.

**Fig. 7:**
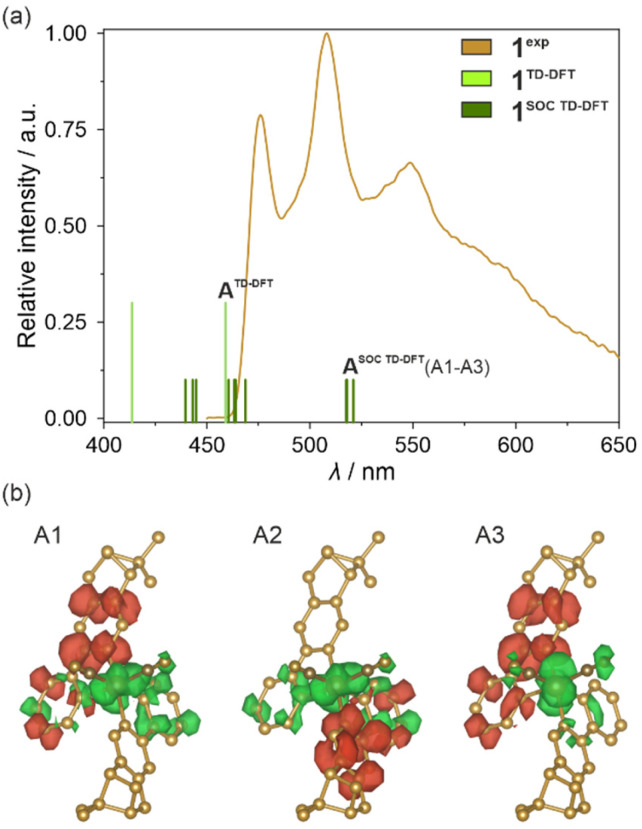
Visible light emission spectra of 1 in the solid state (1^exp^, *λ*_exc_ = 400 nm, 10 K) compared with the energy positions of emissive electronic transitions obtained from TD-DFT (1^TD-DFT^, including the lowest-energy state marked as A^TD-DFT^) and SOC-corrected TD-DFT (1^SOC TD-DFT^, including the lowest-energy state marked as A^SOC TD-DFT^ which is splitted into three components, A1–A3) calculations performed for *cis*-[Ir^III^(CN)_2_(*R*,*R*-pinppy)_2_]^−^ units (a) (see the text, Fig. S26 and S27, Tables S17 and S18†), and differential electron density maps for three low-lying SO-states (A1–A3) for the optimized geometry of the mentioned Ir(iii) complex in their first excited SO-state (b), which represent the changes in electron density (positive changes in red, negative changes in green) occurring upon optical transitions responsible for three selected absorption bands in the geometry of the indicated excited states. The densities were plotted with an isosurface level of 0.0016.

In the next step, the analogous type of TD-DFT calculations were performed for the optimized excited state geometry of the Ir(iii) complex, as electronic states extracted for this configuration should represent light emission from the excited state. First, a set of molecular orbitals and energies of lowest-lying singlet and triplet states were obtained but their highly delocalized character was identical to those previously found for the characteristics of the ground state geometry (Fig. S26 and Table S17†). Next, the previously described SOC approach was applied. The resulting set of TD-DFT-calculated singlet states and the set of singlet–triplet SO-states are gathered in Table S18.† Their parts corresponding to the 400–650 nm range were further compared with the solid-state emission pattern of 1 which is presented in Fig. 7a. The differential electron density maps for the set of three lowest-lying SO-states (A1–A3 of the A^SOC TD-DFT^ group marked in Fig. 7a) were visualized in Fig. 7b while their comparison with the next, higher-lying one, is presented in Fig. S27.† The lowest-lying TD-DFT state (marked as A^TD-DFT^ in Fig. 7a) lies at *ca.* 459 nm which well corresponds to the highest-energy emission band of 1, revealing the maximum at 476 nm at 10 K (Fig. 7a). Thus, the emission of 1 can be assigned to the electronic transition from this lowest-lying TD-DFT state which agrees with Kasha's rule; then, the lower-energy emission bands can be ascribed to the vibronic structure appearing due to the electron–phonon coupling. As already discussed in the Light absorption and photo-luminescence studies section, the energy differences between the main emission components in 1, as well as in 2–4, vary within the range of 1200–1400 cm^−1^. Thus, they match well the energies of stretching vibrations of aromatic C–C bonds of (*R*,*R*)-pinppy ligands (Fig. 4 and S1†) which can be then assigned as the main source of the vibronic structure of the observed emission patterns. The spin–orbit correction shifts the lowest-energy TD-DFT state to lower energies and leads to the energy splitting of the lowest-energy level into three states (A1–A3 of the A^SOC TD-DFT^ group marked in Fig. 7a) leading to the theoretical emission maxima in the narrow range of 517–520 nm. These three states are placed close to each other (within *ca.* 165 cm^−1^ range), and at a large energy distance to the higher-lying ones (placed above 21 300 cm^−1^, *i.e.*, below 470 nm, also shown in Fig. 7a); therefore, the emission can occur from the set of all these three closely-lying excited states. The calculated energy positions of these SO-states are still within the range of the observed experimental emission pattern; however, they are situated at lower energies than the maximum of the higher-energy emission band of 1 (476 nm at 10 K). This discrepancy can be assigned to the character of the TD-DFT calculations that were performed for the isolated Ir(iii) complex while the emission of 1 (and also other compounds) represents the Ir(iii) complex placed in the crystalline solids. Nevertheless, both the TD-DFT and SOC-corrected TD-DFT approaches lead to the conclusion that the observed emission in 1 can be assigned to the electronic transition from the lowest-lying TD-DFT/SOC-corrected TD-DFT states while the lower-energy emission bands are due to the vibronic structure.

In this context, the character of the emission from investigated Ir(iii) complexes can be discussed based on the differential electron density maps for the lowest-lying A-type states (A1–A3, Fig. 7b). In principle, these differential electron density maps represent the light absorption of investigated molecules in the geometry of an excited state so to analyze emission it is necessary to simply invert the sign of the differential density. For clarity, we kept the convention of absorption transition, so the red and green colors represent the positive and negative changes in electron density upon absorption transition, respectively. Therefore, under this interpretation, all calculated lowest-lying SO-states here indicate that the emission is related to the flow of the electron density from both (*R*,*R*)-pinppy ligands to mainly Ir(iii) centers, partially to the other parts of the same ligands and cyanido ligands. This undoubtedly proves that the emission observed for [Ir^III^(CN)_2_(*R*,*R*-pinppy)_2_]^−^ complexes, not only in 1 but also in the whole series of obtained compounds, is of a charge transfer (CT) character involving mainly Ir(iii) center and (*R*,*R*)-pinppy, and to some extent cyanido, ligands. Keeping the classical convention of naming the excited state from which the emission originates, this emission can be described as the metal-to-ligand charge transfer (MLCT). The small admixture of intraligand transitions can be also postulated when analyzing the flow of electron density upon the emission transition. Further evidence of a charge transfer character of the emission for investigated Ir(iii) complexes is the strong dependence of the wavelengths of emission maxima on the concentration that was examined for soluble compound 1 (Fig. S28†).

As stated above, according to Kasha's rule, the emission in 1–4 results only from the lowest-energy electronic state; thus, the observed three-component structure of the emission patterns can be ascribed to the coupling with vibrational modes. The related energy splitting suggests that the main role is here played by stretching vibrations of aromatic C–C bonds of (*R*,*R*)-pinppy. In this regard, the optical thermometry, observed in 1–4 (Fig. 5), can be assigned to the variable ratio between the emission components ascribable to the electronic transitions from the bottom (lowest-energy vibrational level) of the lowest-lying excited MLCT state of Ir(iii) complexes to three different vibrational levels of the ground electronic state. Therefore, the thermometric behavior can be assigned to different thermal quenching of these electronic transitions through the overlap with other thermally activated vibrational modes of the compounds. Interestingly, the strongest thermal effect, *i.e.*, the strongest temperature dependence of the intensity, was observed for higher-energy components below 530 nm while a weaker thermal effect was found for the lower-energy emission bands above 530 nm (this is an origin of ratiometric thermometry, see Fig. 5). This phenomenon cannot be easily explained and the additional thorough computational methods will be needed to elucidate the temperature variation of vibrational sublevels in such multicomponent crystalline solids as 1–4; however, the important role should be played by some thermally activated vibrations responsible for quenching of the observed emission. This effect was found to be strongly dependent on the compound along the 1–4 series which indicates that the vital role might be played by cyanido ligand stretching vibrations related to two cyanido ligands coordinated to Ir(iii) centers in all obtained materials. They are expected to influence the emission quenching as they directly contribute to the observed MLCT luminescence (Fig. 7b). In this context, concerning the temperature working range of the thermometric effect (Fig. 5), 1, which is a simple TBA^+^ salt of the emissive Ir(iii) anionic complex, reveals the lowest-temperature optical thermometry with the highest sensitivity at *ca.* 20 K (Fig. 5d). The higher temperature working range with the maximal *S*_r_ at *ca.* 45 K is observed for the next molecular hybrid salt of 2. In both of these compounds, the cyanido ligands are only terminal, thus the related vibrations are situated at slightly lower energies than in 3 and 4 (Fig. S1†). There is, however, a difference between 1 and 2, as the latter shows the maximal *S*_r_ at a noticeably higher temperature which correlates well with the slightly higher energy of cyanido vibrations ascribable to their involvement in stronger interactions, including the incorporated heavy iodine atom. Finally, the highest operating temperature range of thermometry with the maximal *S*_r_ above 80 K is observed for 3 and 4 where the Ir(iii) complex is attached through cyanido bridges to the heavy La(iii) centers. The energies of the cyanido stretching vibrations are then increased as typically observed for bridging cyanido ligands,^17^ which can be the reason for their activation at relatively higher temperatures. This can lead to an increased *T*-range of the observed thermometric effect in 3 and 4. Presumably, there are also other vibrational modes contributing to the thermometric effect, especially when the additional components, such as nitrate and aqua ligands in 3 or dmf ligands in 4, appear in the structure of reported compounds. Such the modulated mixture of variously *T*-activated vibrational modes is presumably crucial in tuning the performance of the thermometric effect which was found the most efficient in the coordination polymer case of 4. Further detailed discussion on the tunable electron–phonon coupling and its role in optimizing the thermometric behavior of the investigated Ir(iii) complexes will demand performing the advanced periodic TD-DFT calculations which is an extremely difficult task and stay beyond the scope of this work.^82^

## Conclusions

We report a unique member of a family of photoluminescent cyclometalated Ir(iii) complexes, [Ir^III^(CN)_2_(*R*,*R*-pinppy)_2_]^−^, which contains two enantiopure (*R*,*R*)-2-phenyl-4,5-pineno-pyridine ligands ensuring the chiral structure and two cyanido ligands opening the convenient route for the synthesis of diverse supramolecular and coordination assemblies with the obtained Ir(iii) anionic complex. As a proof-of-concept, we report a series of four molecular materials incorporating the above-mentioned chiral Ir(iii) luminophore. This family includes two molecular hybrid salts with TBA^+^ counter-ions (1) or *n*Bu-DABCO^+^ and iodide ions (2), a molecular {La^III^_2_Ir^III^_2_} cyanido-bridged squares (3), and La^III^–Ir^III^ coordination polymers based on cyanido bridges (4). All these compounds demonstrate the combination of (a) SHG activity related to the lack of an inversion center due to the presence of enantiopure ligand, (b) room-temperature photoluminescence (PL) related to the MLCT electronic transitions within the Ir(iii) complexes, and (c) ratiometric optical thermometry observed thanks to the different temperature variations of low- and high-energy components of the MLCT emission. Therefore, we present rare examples of SHG-active luminescent molecular thermometers. We proved that chiral pinene-functionalized cyclometalated dicyanidoiridate(iii) complexes offer an efficient route for the SHG-PL (more generally, NLO-PL) conjunction enriched by ratiometric optical thermometry. Moreover, we found that this multifunctionality can be tuned by incorporation of the Ir(iii) complexes into various molecular materials. We found that the SHG activity is greatly enhanced when the Ir(iii) complex is embedded into a d-f coordination polymer, which is particularly visible as the supramolecular frameworks of 1 and 2 crystallize in the same *P*2_1_2_1_2_1_ space group. The PL property with the quantum yields at the 11–22% level is ensured for all materials by an MLCT emission, typical for cyclometalated Ir(iii) complexes. More importantly, the optical thermometry effect was achieved thanks to the variable temperature dependences of two different emission components observed within the emission pattern of Ir(iii) complexes. We showed, using the experimental data and the results of TD-DFT calculations, that this ratiometric optical thermometry can be rationalized by the vibronic structure of the emission, appearing due to the coupling with the stretching vibrations of aromatic C–C bonds of (*R*,*R*)-pinppy ligands, and the further role of other thermally activated vibrational modes, *e.g.*, related to cyanido ligands. This appears to be the intrinsic property of such Ir(iii) complexes, thus this ability can be transferred into many other molecular materials bearing this metalloligand. The optical thermometric effect could be also tuned and, similarly to SHG, was found to be the most efficient for the coordination polymer case. Therefore, the future improvement of the NLO-PL bifunctionality should be achieved by the insertion of chiral dicyanidoiridate ions into highly-dimensional heterometallic frameworks with other f- or d-block metal ions. This pathway should also provide an enriched scope of properties by using magneto-luminescent lanthanide centers or testing the chiroptical properties, *e.g.*, CPL.

## Author contributions

The manuscript was written through the contributions of all authors. All authors have approved the final version of the manuscript.

## Conflicts of interest

There are no conflicts to declare.

## Supplementary Material

QI-011-D3QI02482B-s001

QI-011-D3QI02482B-s002
