# Supplementary material for: SHG-active luminescent thermometers based on chiral cyclometalated dicyanidoiridate(iii) complexes
Source: Inorg Chem Front. 2024 Jan 26;11(5):1366–80. doi: 10.1039/d3qi02482b (PMC10897766; doi:10.1039/d3qi02482b)
Supplement: QI-011-D3QI02482B-s001 [file QI-011-D3QI02482B-s001.pdf]

Electronic Supplementary Material (ESI) for Inorganic Chemistry Frontiers.
This journal is © the Partner Organisations 2024

Supporting information

SHG-active luminescent thermometers based on chiral cyclometalated dicyanidoiridate(III) complexes

Jan Rzepiela,[a,b] Michal Liberka,[a,b] Mikolaj Zychowicz,[a,b] Junhao Wang,[c,d] Hiroko Tokoro,[c] Kinga Piotrowska,[a,b] Sebastian Baś,[a] Shin-ichi Ohkoshi,[d] and Szymon Chorazy*,[a]

[a]Faculty of Chemistry, Jagiellonian University, Gronostajowa 2, 30-387 Kraków, Poland; [b]Jagiellonian University, Doctoral School of Exact and Natural Sciences, Łojasiewicza 11, 30-348 Kraków, Poland; [c]Department of Materials Science, Faculty of Pure and Applied Science, University of Tsukuba, 1-1-1 Tennodai, Tsukuba, Ibaraki 305-8573, Japan; [d]Department of Chemistry, School of Science, The University of Tokyo, 7-3-1 Hongo, Bunkyo-ku, Tokyo 113-0033, Japan.

*Corresponding author: simon.chorazy@uj.edu.pl

Experimental details.	S2–S5
Infrared (IR) spectra of compounds **1**–**4** and **(*R,R*)-pinppy** (Figure S1) with the comment.	S7
Thermogravimetric (TG) curves for compounds **1**–**4** (Figure S2) with the comment.	S8
Details of the crystal data and structure refinement for **1**–**4** and **(*R,R*)-pinppy** (Table S1).	S9
Representative views of the crystal structures of **(*R,R*)-pinppy** and compounds **1**–**4** (Figures S3–S7).	S10–S15
Detailed structure parameters of **(*R,R*)-pinppy** and compounds **1**–**4** (Tables S2–S5).	S11–S16
Results of Continuous Shape Measure (CShM) analysis for compounds **1**–**4** (Table S6).	S17
The experimental and calculated P-XRD patterns for the polycrystalline samples of **1**–**4** (Figure S8).	S18
Wavelength and power dependences of the SHG effect for the powder samples of compounds **1**–**4** (Figures S9 and S10).	S19–S20
Solid-state UV-vis absorption spectra of **(*R,R*)-pinppy** and compounds **1**–**4** (Figure S11).	S21
Temperature dependences of the excitation and emission spectra for **(*R,R*)-pinppy** and compounds **1**–**3** (Figures S12–S15).	S21–S25
The positions of well-distinguished maxima of the solid-state emission patterns of **1–4** at various indicated temperatures (Tables S7–S10).	S23–S27
Temperature dependences of the excitation and emission spectra as well as selected thermometric parameters for compound **4** (Figure S16).	S26
Comparison of selected optical thermometry characteristics for compounds **1–4** (Figure S17).	S28
Comment on Figures S16 and S17.	S29
Comparison of best-fit parameters to the thermometric calibration curves for **1**–**4**. (Table S11)	S30
Thermal changes in emission colors for **1**–**4**, shown on the CIE 1931 chromaticity diagram (Figure S18).	S31
Emission decay profiles for **1**–**4**, the related best-fit parameters for emission lifetimes, and the temperature dependences of the emission lifetimes for **3** and **4** (Figures S19–S22, Tables S12 and S13).	S32–S35
Computational details.	S36
Results of DFT and TD-DFT calculations and their visualization (Figures S23–S27, Tables S14–S18)	S37–S45
Comparison of theoretical and experimental emission patterns of the Ir(III) complexes of **1** (Figure S28).	S46
References to the Supporting Information.	S47–S48

Experimental Details

Materials

Starting reagents and solvents were purchased from Merck or TCI Chemicals and used without any purification.

All stages of the synthetic part are visualized in Scheme 1 (see the main text for details).

Synthesis of (*R,S*)-myrtenal

To the solution of SeO_2 (1.33 g, 12 mmol, 1.2 eq) in 20 ml of anhydrous ethanol was slowly added **(*S,S*)-pinene** (1.59 mL, 10 mmol, 1.0 eq). The resulting mixture was refluxed for 18 h, cooled, and filtered through celite. The resulting filtrate was evaporated on a rotary evaporator, devolved in diethyl ether, and washed with water and brine. The organic phase was dried over anhydrous magnesium sulfate, evaporated, and purified by silica gel column chromatography (Hx:AcOEt, 95:5) resulting in a slightly yellow liquid. Yield, 0.93 g (62%).

Rf: 0.42 (Hx:AcOEt, 95:5)

^{1}H NMR (600 MHz, $CDCl_3$, Me_4Si) δ 9.44 (s, 1H), 6.72 (tt, J = 3.2, 1.5 Hz, 1H), 2.87 (td, J = 5.7, 1.5 Hz, 1H), 2.59 (t, J = 3.2 Hz, 1H), 2.55 (t, J = 3.0 Hz, 1H), 2.49 (dt, J = 9.2, 5.6 Hz, 1H), 2.19 (ttd, J = 5.7, 2.8, 1.4 Hz, 1H), 1.34 (s, 3H), 1.06 (d, J = 9.2 Hz, 1H), 0.75 (s, 3H).

^{13}C{^{1}H} NMR (151 MHz, $CDCl_3$, Me_4Si) δ 191.4, 151.7, 148.0, 40.8, 37.7, 33.1, 31.2, 25.8, 21.0.

The NMR data were found to be in good agreement with the data reported in the literature.[S1]

Synthesis of 1-(2-oxo-2-phenylethyl)pyridin-1-ium bromide (P2)

To the solution of 2'-bromoacetophenone (2 g, 10 mmol, 1.0 eq; **P1** in Scheme 1) in 10 ml of anhydrous dichloromethane under Ar atmosphere, pyridine (0.89 mL, 11 mmol, 1.1eq) was added and the resulting mixture was stirred overnight. The white precipitate of the product **P2** appeared. It was filtered through a sintered glass funnel, washed with a small amount of cold dichloromethane, and dried under a vacuum. Yield, 2.77g (99.6%).

^{1}H NMR (600 MHz, DMSO-d6) δ 9.15 (dd, J = 6.6, 1.1 Hz, 2H), 8.78 (tt, J = 7.9, 1.4 Hz, 0H), 8.34 – 8.30 (m, 1H), 8.09 (dd, J = 8.3, 1.2 Hz, 2H), 7.82 – 7.78 (m, 0H), 7.67 (t, J = 7.8 Hz, 1H), 6.70 (s, 1H).

^{13}C{^{1}H} NMR (151 MHz, DMSO-d_6) δ 191.4, 151.7, 148.0, 40.8, 37.7, 33.1, 31.2, 25.8, 21.0.

The NMR data were found to agree with the data reported in the literature.[S2]

Synthesis of (*R*,*R*)-2-phenyl-4,5-pineno-pyridine ((*R*,*R*)-pinppy)

To the ACE pressure vessel, the solution of **P2** (2.04 g, 7.3 mmol, 1.1 eq) and NH_4OAc (1.03 g, 13.3 mmol, 2.0 eq) was added, and the vessel was filled with the 25 ml portion of anhydrous ethanol. To this mixture, **(*R,S*)-myrtenal** (1.01 ml, 6.7 mmol, 1.0 eq) was added, then the pressure vessel was closed and heated to 110 °C overnight. After cooling, the reaction mixture was poured into water (50 ml) and extracted with dichloromethane (3×75 mL). The combined organic layers were dried over anhydrous magnesium sulfate, evaporated, and purified by column chromatography of silica gel (Hx:AcOEt, 95:5). Yield, 1.11 g (61%). The single crystals of **(*R,R*)-pinppy** were obtained by crystallization from pentane. Its composition and phase purity were determined by ^{1}H NMR and ^{13}C{^{1}H} NMR studies, supported by single-crystal X-ray diffraction (SC-XRD) analysis.

Rf: 0.30 (Hx:AcOEt, 95:5)

^{1}H NMR (600 MHz, $CDCl_3$, Me_4Si) δ 8.22 (s, 1H), 7.98 – 7.95 (m, 2H), 7.52 (s, 1H), 7.47 – 7.43 (m, 2H), 7.40 – 7.36 (m, 1H), 3.03 (d, J = 2.7 Hz, 3H), 2.86 (t, J = 5.5 Hz, 1H), 2.72 (dt, J = 9.6, 5.8 Hz, 1H), 2.32 (tt, J = 5.8, 2.8 Hz, 1H), 1.43 (s, 3H), 1.25 (d, J = 9.7 Hz, 1H), 0.67 (s, 3H).

^{13}C{^{1}H} NMR (151 MHz, $CDCl_3$, Me_4Si) δ 155.8, 146.0, 145.3, 141.3, 140.0, 128.8, 128.5, 126.8, 120.2, 44.5, 40.2, 39.5, 33.0, 32.0, 26.2, 21.6.

The NMR data were found to agree with the data reported in the literature.[S3]

Synthesis of *n*Bu-DABCO^{+}I^{-}

DABCO (1,4-Diazabicyclo[2.2.2]octane, 840 mg, 7.5 mmol, 1.0 eq) was dissolved in 22.5 ml of diethyl ether and 1-iodobutane (1.28 mL, 11.25 mmol, 1.5eq) was added to the resulting solution. The reaction was carried out at room temperature for 24 hours. The resulting precipitate of ***n*Bu-DABCO^{+}I^{-}** was filtered through a sintered glass funnel, washed with diethyl ether, and dried under vacuum. Yield, 1.92 g (95%).

^{1}H NMR (600 MHz, CD_3OD) δ 3.47 (t, J = 7.5 Hz, 4H), 3.33 (t, J = 3.9 Hz, 1H), 3.25 (t, J = 7.5, 5.5 Hz, 4H), 1.86 – 1.77 (m, 1H), 1.86 – 1.77 (m, 1H), 1.46 (h, J = 7.4 Hz, 1H), 1.06 (t, J = 7.4 Hz, 2H).

^{13}C{^{1}H} NMR (151 MHz, CD_3OD) δ 65.6, 65.6, 65.5, 53.5, 53.5, 53.4, 46.1, 24.9, 20.8, 13.9.

The NMR data were found to correspond well to the data reported in the literature.[S4]

Synthesis of 1

The three-step synthesis of **1** started by mixing iridium(III) chloride hydrate (200 mg, 0.67 mmol, 1.0 eq) and **(*R*,*R*)-pinppy** (417 mg, 1.68 mmol, 2.5eq) with 15 ml of ethoxyethanol and 5 mL of water. After 24 h refluxing this mixture, the resulting solid product was precipitated with water, filtrated on a sintered glass funnel, washed with water and pentane, and dried. The yellow solid was dissolved in dcm, dried over anhydrous magnesium sulfate, and evaporated to dryness. (Yield, 436 mg, ca. 90%). Secondly, the as-prepared [Ir^{III}_2(μ_2-Cl)$_2$(*R*,*R*-pinppy)$_4$] (436 mg, 0.30 mmol, 1 eq) was dissolved in the mixture of methanol (100 ml) and dcm (50 mL). Then, KCN (80 mg, 1.23 mmol, 4.1 eq) was added and the reaction mixture was refluxed for 24 h. After being cooled, the reaction mixture was filtered, and the resulting filtrate was evaporated. The resulting potassium salt, K[Ir^{III}(CN)$_2$(*R*,*R*-pinppy)$_2$] was dried and used directly in the last step. Therefore, the entire product was dissolved in 100 ml of methanol, and 215 mg of tetrabutylammonium (TBA) perchlorate (0.63 mmol, ca. two-time excess) was added. The mixture was refluxed for 24 h and the resulting precipitate was filtered. The filtrate was evaporated to dryness, dissolved in a small amount of dichloromethane (dcm), filtered, and the product was precipitated by diethyl ether. The two last steps resulted in 560 mg (95% yield) of the yellow powder of the objective compound **1**. A single crystal of **1** was obtained by slow diffusion of diethyl ether into an acetonitrile (MeCN) solution of the compound. Its composition, TBA[Ir^{III}(CN)$_2$(*R*,*R*-pinppy)$_2$]·2MeCN (M_W = 1065.47 g·mol^{-1}, **1**), was determined by single-crystal X-ray diffraction (SC-XRD) analysis, while the phase purity and its air-stability were proven by the powder X-ray diffraction (P-XRD) method (Fig. S8). The crystals of **1** are quite hygroscopic and, while left in the air, they adsorb water molecules (generating the hydrated form of **1·4H$_2$O**), which does not cause any distinct structural transformation, as evidenced by the results of the P-XRD and CHN elemental analysis. These water molecules can be easily removed by an inert gas purge, which was confirmed by the results of the TG experiment (see Fig. S2 with the related comment). Elemental analysis calculated for the hydrated form, **1·4H$_2$O** ($C_{58}H_{86}Ir_1N_7O_4$; M_W = 1137.55 g mol^{-1}): C, 61.24%; H, 7.62%; N, 8.62%. Found: C, 61.08%; H, 7.54%; N, 8.34%. TG (Fig. S2 with comments): loss of 2 MeCN molecules together with the loss of 1 remaining H_2O molecule per formula unit, calculated: 9.24 %; found: 9.35 % (as the other water molecules are removed before the start of the TG measurement). IR spectrum (see Fig. S1 with the related comment): the bands located at 2104 and 2096 cm^{-1} can be assigned to the stretching vibrations of terminal cyanido ligands, proving the presence of these ligands in the structure.[S5,S6]

In addition to the set of physical methods presented above, compound **1** was also investigated by the ^{1}H NMR method in the dissolved form.

^{1}H NMR (400 MHz, CD_3Cl, Me_4Si) δ 9.28 (s, 0.84H), 9.23 (s, 1.04H), 7.58 (s, 1H), 7.56 (s, 0.92H), 7.47 – 7.41 (m, 2H), 6.71 (td, *J* = 7.3, 1.4 Hz, 2H), 6.64 (dtd, *J* = 10.7, 7.2, 1.3 Hz, 2H), 6.26 (ddd, *J* = 9.0, 7.3, 1.4 Hz, 2H), 3.38 – 3.29 (m, 8H), 3.16 – 3.13 (m, 4H), 2.95 (t, *J* = 5.5 Hz, 0.86H), 2.90 (t, *J* = 5.4 Hz, 1.05H), 2.72 (ddt, *J* = 15.4, 9.4, 5.7 Hz, 2H), 2.40 – 2.31 (m, 2H), 1.66 – 1.53 (m, 8H), 1.44 (s, 3.23H), 1.43 (s, 2.79H), 1.36 (q, *J* = 9.3, 8.3 Hz, 8H), 1.29 – 1.23 (m, 2H), 0.95 (t, *J* = 7.3 Hz, 12H), 0.81 (s, 3.25H), 0.77 (s, 2.65H).

Comment on NMR studies of 1: Only signals from the complex anion and TBA^{+} cation were reported above. Signals from acetonitrile and water molecules are visible in the spectrum but were omitted for the clarity of

interpretation. A signal at around 1.57 ppm related to the -CH_2- protons of TBA^+ ion is affected by the water signal. Underlined signals in the ^1H NMR are connected with the protons that are different between the diastereoisomers (*RRΛ* and *RRΔ*), thus their integration can be used to estimate the ratio of diastereoisomers as 1.25 to 1. We were not able to measure ^{13}C NMR with satisfactory results, due to the low solubility of the complex in deuterated chloroform and thus low signal-to-noise ratio. Moreover, NMR studies did not allow us to determine which diastereomer is major. According to these results, and the fact that all other molecular materials were obtained from an identically prepared sample of **1**, it can be assumed that not only compound **1** but also further materials **2–4** are mixtures of diastereisomers. The analogous NMR studies for further compounds **2**–**4** were, however, not performed as they reveal more complex structures that are expected not to be preserved in the solution due to the dissociation (see the details on these compounds below).

Synthesis of 2

The 25 mg portion of freshly filtrated crystals of **1** (0.025 mmol, 1.0 eq) were dissolved in a mixture of methanol (1 ml) and acetonitrile (1 mL), and the 15 mg portion of *n*-$BuDABCO^+I^-$ (0.05 mmol, 2 eq) was added directly to this solution. After 5 min of stirring, the mixture was filtrated and diethyl ether was layered on top of the solution. After 2 weeks, a large number of yellow plate crystals of **2** appeared. Yield, 15 mg (50%). Their composition, $(n\text{Bu-DABCO})_2[\text{Ir}^{\text{III}}(\text{CN})_2(R,R\text{-pinppy})_2](\text{I})\cdot\text{MeCN}$ (M_W = 1247.43 g·mol^{-1}, **2·MeCN**), was determined by SC-XRD analysis, Exposure of the crystals of **2** to the air causes the loss of acetonitrile molecules and adsorption of water molecules (generating the hydrated form, **2·2H₂O**). It does not cause any structural transformation which was confirmed by P-XRD results (Fig. S8), CHN elemental analysis, and TG experiment (see Fig. S2 with the related comment).

Elemental analysis calculated for the hydrated form, **2·2H₂O** ($C_{58}H_{82}I_1Ir_1N_8O_2$; M_W = 1242.52 g mol^{-1}): C, 56.07%; H, 6.65%; N, 9.02%. Found: C, 56.55%; H, 6.67%; N, 9.51%. TG (Fig. S2 with the comment): loss of 2 H_2O molecules per formula unit, calculated: 2.90 %; found: 3.14 %. IR spectrum (Fig. S1 with the related comment): the bands located at 2102 and 2096 cm^{-1} indicate the presence of stretching vibrations of terminal cyanido ligands.[S5]

Synthesis of 3

The equimolar solution of freshly filtrated crystals of **1** (31 mg, 0.0315 mmol) and lanthanum nitrate hexahydrate (14 mg, 0.0315 mmol) in 2 mL of acetonitrile and 0.1 ml of MeOH was slowly diffused by diethyl ether. After a few days, well-shaped yellow crystals of **3** appeared. Yield, 26 mg (63%). Their composition, $(\text{TBA})_2\{[\text{La}^{\text{III}}(\text{NO}_3)_3(\text{H}_2\text{O})_{0.5}]_2[\text{Ir}^{\text{III}}(\text{CN})_2(R,R\text{-pinppy})_2]_2\}$ (M_W = 2632.60 g·mol^{-1}, **3**), was determined by SC-XRD analysis, while the purity of the phase together with its air stability were proven by the P-XRD method (Fig. S8). Similarly to **1**, the exposition of **3** in the air causes the surface adsorption of water molecules (generating the hydrated form **3·4.5H₂O**), occurring without any structural transformation, which was confirmed by P-XRD, CHN elemental analysis, and TG results. Note that one coordinated water molecule per the formula unit is present in **3** while another 4.5 water molecules per the formula unit appear as the solvent of crystallization in the hydrated form, **3·4.5H₂O**. Thus, in the TG experiment, a loss of the total number of 5.5 water molecules per the formula unit is observed (for details regarding the TG, see Fig. S2 with the related comment).

Elemental analysis calculated for the hydrated form, **3·4.5H₂O** ($C_{108}H_{154}Ir_2La_2N_{16}O_{23.5}$; M_W = 2713.69 g mol^{-1}): C, 47.76%; H, 5.75%; N, 8.25%. Found: C, 47.92 %; H, 5.70 %; N, 8.33%. TG (Fig. S2 with the comment): loss of 5.5 H_2O molecules per formula unit, calculated: 3.65%; found: 3.63%. IR spectrum (Fig. S1 with the related comment): the bands situated at 2110 and 2097 cm^{-1} can be assigned to cyanido ligands; they are noticeably shifted towards higher energy than, e.g., in the ionic salt of **2** which suggests their bridging character.[S5]

Synthesis of 4

The yellow crystals of **4** were obtained by slow diffusion of diethyl ether into the equimolar solution of freshly filtrated crystals of **1** (31 mg, 0.0315 mmol) and lanthanum nitrate hexahydrate (14 mg, 0.0315 mmol) in the solvents mixture containing 1 ml of acetonitrile, 0.1 ml of methanol and 0.1 mL of dimethylformamide. Yield, 22 mg (55%). Their composition, {[$La^{III}(NO_3)_2$(dmf)$_3$][$Ir^{III}(CN)_2$(*R*,*R*-pinppy)$_2$]}·MeCN (M_W = 1264.18 g·mol^{-1}, **4**), was determined by SC-XRD analysis, while the phase purity together with its air stability were proven by the P-XRD method (Fig. S8). In the air, the crystals of **4** adsorb water molecules (generating the hydrated form of **4·2H$_2$O**), which can be removed by an inert gas purge, as confirmed by P-XRD, CHN elemental analysis, and TG results (for details, see Fig. S1 with the related comment).

Elemental analysis calculated for the hydrated form, **4·2H$_2$O** ($C_{49}H_{64}Ir_1La_1N_{10}O_{11}$; M_W = 1300.22 g mol^{-1}): C, 45.26%; H, 4.96%; N, 10.77%. Found: C, 45.54%; H, 4.97%; N, 10.51% TG (Fig. S2 with the comment): loss of 3 dmf molecules together 1 MeCN molecule per formula unit, calculated: 20.59%; found: 20.30%. IR spectrum (Fig. S1 with the related comment): the bands situated at 2119 and 2092 cm^{-1} can be assigned to cyanido ligands; they are noticeably shifted towards higher energy than, e.g., in the ionic salt of **2** which indicates their bridging character.[S5]

Additional comment on the description of syntheses of compounds 1, 3, and 4: All obtained materials are generally air-stable; however, the prolonged exposition to the air leads to the adsorption of small amounts of water molecules (as determined by the CHN elemental analysis). As confirmed by structural studies, this process does not cause structural changes. We suppose that this adsorption takes place only on the surface of fine crystalline samples of **1, 3**, and **4** because most of these water molecules can be easily and quickly removed by an inert gas purge (the detailed analysis of this effect is presented as the comment to Fig. S2, see below). Therefore, all physical measurements were carried out on freshly filtrated compounds which eliminated the adsorption of moisture as much as possible.

Structural studies

Single crystal X-ray diffraction (SC-XRD) analysis for all compounds was performed at 100(2) K, using a Bruker D8 Quest Eco Photo50 CMOS diffractometer, equipped with the Mo Kα (0.71073 Å) radiation source and a graphite monochromator. The single crystals of **(*R*,*R*)-pinppy** ligand and compounds **1–4** selected for the SC-XRD experiments were taken directly from the respective mother solutions, covered by Apiezon N grease, and mounted on the dedicated Micro Mounts™ holders. The SAINT and SADABS programs were used for data reduction and cell refinement processes. The absorption correction was performed using a multi-scan procedure with the help of the TWINABS program. The crystal structures were solved by an intrinsic phasing method using a SHELXT program implemented within the Apex3 package.[S7] The further refinements were carried out by a weighted full-matrix least squares method on F^2 of SHELX-2014/7 within the WinGX (ver. 2018.3) software.[S8] All non-hydrogen atoms were anisotropically refined, while hydrogen atoms for (*R*,*R*)-pinppy ligands, aliphatic *n*Bu-DABCO$^+$, and TBA$^+$ cations, as well as MeCN solvent molecules were calculated in their idealized positions and refined using a riding model. The methine hydrogen atoms in dmf molecules were found directly from an electron density map. A reasonable number of restraints of the DFIX, ISOR, and DELU types were applied for the selected non-hydrogen atoms of (*R*,*R*)-pinppy ligands, counter-cations, coordinated (but highly disordered) nitrate(V) anions, and the part of solvent molecules. It was done to ensure the proper geometries and the convergence of the respective refinement procedures. Due to the significant disorder, two DFIX restraints were applied to the La−O bond distances in **3** to ensure its proper geometry and keep the convergence of the refinement. For similar reasons, the FLAT restraint was applied for one of the coordinated NO_3^- anions in the crystal structure of **3**. Some of the reflections with intensities endowed with especially large errors (affected by the beamstop) were removed from the final refinement using the OMIT commands. Using all of these procedures, the sets of satisfactory refinement parameters were achieved. The reference CCDC numbers for the

crystal data of **(*R*,*R*)-pinppy** and compounds **1**, **2**, **3**, and **4** are 2297257, 2297255, 2297258, 2297256, and 2297254, respectively. Details of the crystal data and refinement of the structure are summarized in Table S1, while representative structural parameters are gathered in Tables S2–S6. The structural figures (Fig. 2, 3, and S3–S7) were prepared using Mercury 3.8 software. Powder XRD data were collected using a Bruker D8 Advance Eco powder diffractometer equipped with a Cu Kα (1.5419 Å) radiation source. The P-XRD measurements were conducted at room temperature for the dried polycrystalline samples inserted into the 0.5 mm glass capillaries using the appropriate commercial experimental setup for rotating capillaries (Fig. S8).

Physical techniques

The ^{1}H and $^{13}C\{^{1}H\}$ NMR spectra were recorded for the proper solutions of investigated organic compounds at room temperature using a Bruker AVANCE 600 MHz spectrometer, while the ^{1}H NMR spectrum for **1** (in solution) was recorded at room temperature using a Jeol 400 MHz ECZR spectrometer (see the details in the Synthesis section above). All solid-state physical measurements were performed on freshly prepared and filtrated samples (see the comment to the description of the syntheses above). CHN elemental analyses were performed with standard microanalysis procedures using the Elementar Vario Micro Cube CHN analyzer. The infrared (IR) absorption spectra were collected on a Nicolet iN10 MX FT-IR microscope in transmission mode. Measurements were made in the range of 3800−670 cm^{-1} for selected single crystals placed on CaF_2 windows. Thermogravimetric (TG) measurements were performed on a NETZSCH TG 209 F1 Libra apparatus under inert gas at a heating rate of 1 $°C\cdot min^{-1}$ in the temperature range of 20–400 °C. The second harmonic generation (SHG) experiment was carried out on a homemade optical setup using a 1040 nm femtosecond laser as an excitation light source. This setup is described in our previous work.[S9] To verify the SHG effect, for all samples **1–4,** the power and wavelength dependencies of the output light were measured. To quantify the SH intensities measured in this setup, a potassium dihydrogen phosphate (KDP) was used as a reference sample. Solid-state UV-vis absorption spectra were measured in the range of 220−750 nm on a Shimadzu UV-3600i plus spectrometer using the thin films of power samples inserted between quartz plates. Solid-state photoluminescent properties were measured using an FS5 spectrofluorometer equipped with an Xe (150 W) arc lamp as an excitation source and a Hamamatsu photomultiplier of the R928P type as a detector. Emission lifetime measurements were conducted on the FS5 spectrofluorometer using a time-correlated single photon counting method with EPLED-380 picosecond pulsed light emitting diode (374.4 nm). The temperature-variable emission and excitation spectra were collected on the same spectrofluorometer using a CS204SI-FMX-1SS cooling power optical helium cryostat which is equipped with a DE-204SI closed cycle cryo-cooler (cold head), water-cooled He compressor (ARS-4HW model), and a model 335 cryogenic temperature controller. For all types of photoluminescent measurements, freshly prepared powder samples were placed between two quartz plates. Absolute luminescence quantum yields (QY) were determined by a direct excitation method using an integrating sphere module for the FS5 spectrofluorometer and barium sulfate as the reference material, following the method described in our previous work.[S10] Luminescent background corrections were performed within the Fluoracle software.

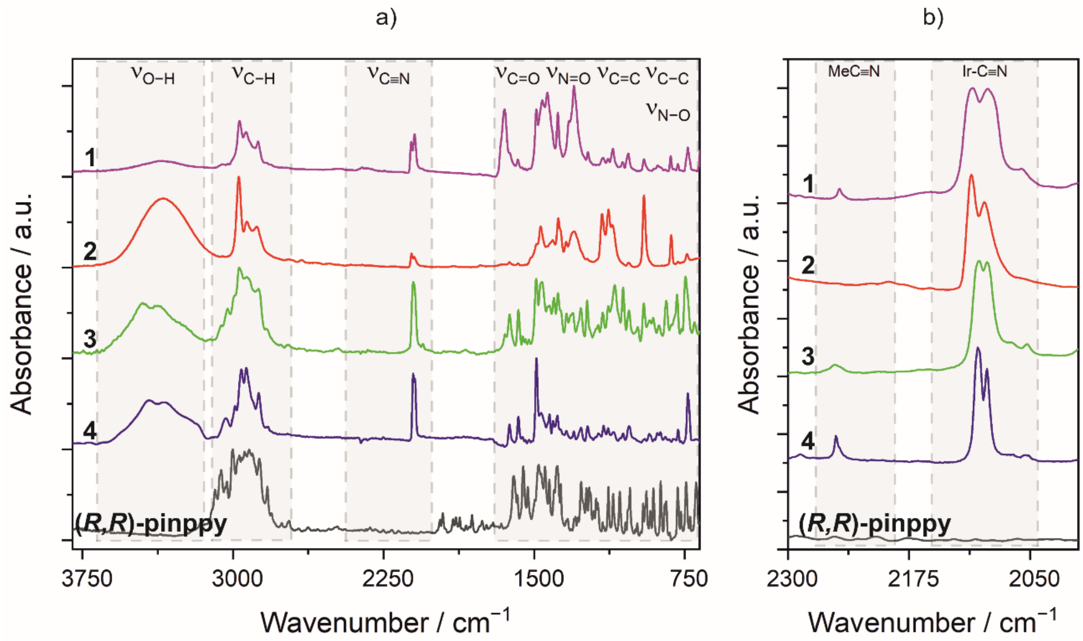

Figure S1. Infrared (IR) absorption spectra of the crystalline samples of **1**, **2**, **3**, **4**, and **(*R*,*R*)-pinppy** ligand presented in (a) the broad range of 3800–670 cm^{-1} and (b) the limited 2300−2000 cm^{-1} range.

Comment on Figure S1: Significant absorption in the range of 3150−2600 cm^{-1} is related to stretching vibrations *ν*(C−H) of the aromatic and aliphatic parts of organic ligands, cations, and solvent molecules. The 1700–670 cm^{-1} range is composed of many characteristic absorption peaks connected with skeletal vibrations such as *ν*(C−C), *ν*(C=C), *ν*(C=O), *ν*(N=O), *ν*(N−O). In part (b), characteristic peaks related to stretching vibrations of cyanido groups/ligands are presented. In this view, the highest energy peaks at ca. 2250 cm^{-1} are related to the acetonitrile molecules of crystallization (in **1** and **4**). In the lower energy region around 2100 cm^{-1}, the peaks related to cyanido ligands are observed. The maxima around 2094 and 2104 cm^{-1} in **1** and **2** correspond to the vibrations of terminal cyanido ligands in [Ir^{III}(CN)$_2$(*R*,*R*-pinppy)$_2$]$^-$ anions. In **3** and **4**, the *ν*(C≡N) energies are slightly shifted to higher wavenumbers which can be ascribed to the coordination of the ligands by lanthanide ions. All observed vibrations in the IR spectra of all compounds are consistent with the structural analysis (see below).

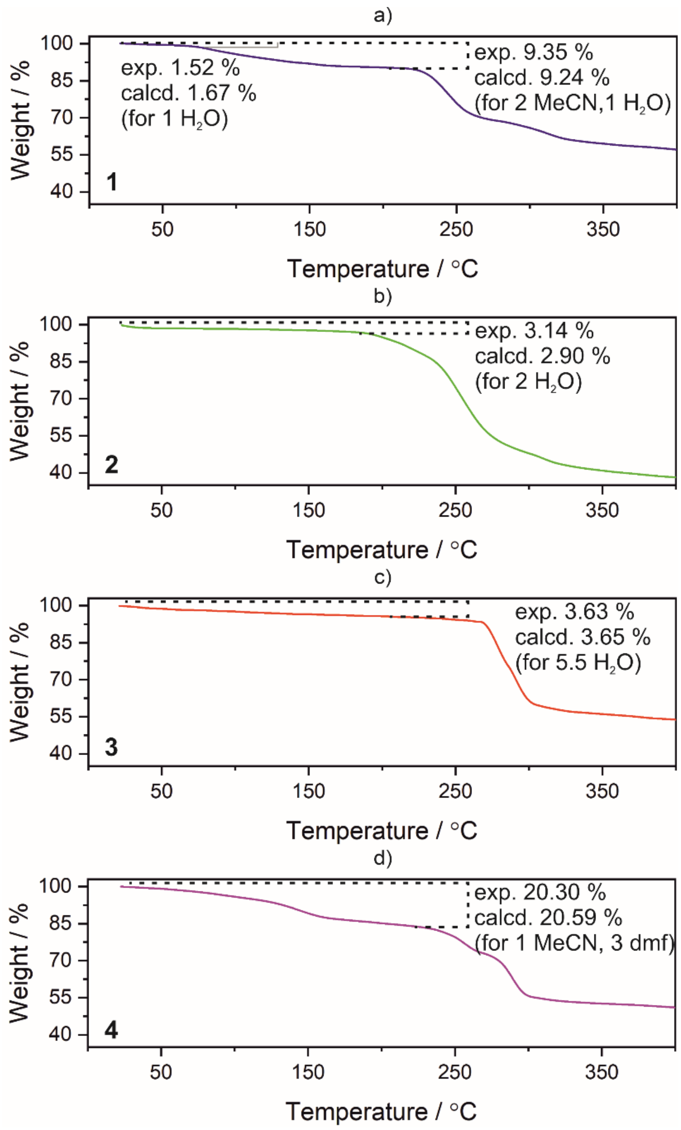

Figure S2. Thermogravimetric (TG) curves, collected in the temperature range of 20–400 °C, for the crystalline samples of (a) **1**, (b) **2**, (c) **3**, and (d) **4**. The steps related to the loss of solvent molecules s are depicted.

Comment on Figure 2: The results of the CHN elemental analysis indicate that the samples adsorb small amounts of water from the air. Moreover, its absorption/removal does not result in a structural transformation, and the above-presented TG results show that most of this solvent is removed in an inert gas purge (even before the start of the TG measurement). Thus, the weight loss in the TG curve of **1** corresponds to two crystallizing acetonitrile molecules and some residual water (about 1 molecule per formula unit). The exposition of the crystals of **2** to air results in the loss of acetonitrile molecules and the adsorption of water molecules. As a result, the thermogravimetric curve of this compound shows the loss of two water molecules. The mass change in the TG curve in **3** corresponds to the weight of 5.5 water molecules per unit of formula, which is consistent with the results of CHN elemental analysis (so, in this case, water molecules are not that easily removed as for the previous cases). Note that among these 5.5 water molecules per the formula unit, one molecule is coordinated to the La(III) center while the remaining 4.5 molecules serve as the solvent of crystallization; however, they are removed under heating in a single, rather featureless, broad step. The sample of **4**, similar to **1** and **2**, when exposed to air, adsorbs about two water molecules per formula unit, which can be removed by a gas purge. As the temperature increased, the removal of the coordinated dmf molecules and the co-crystallizing acetonitrile molecules can be observed. All compounds, **1**–**4** are losing the solvents up to ca. 250 °C. Further heating of the samples causes a rapid decrease in mass, which is related to the decomposition of aliphatic cations and ligands and/or the removal of cyanido ligands, which causes the decomposition of the sample.

Table S1. Crystal data and structure refinement parameters for ligand **(*R,R*)-pinppy** and compounds **1–4**.

Compound	**(*R,R*)-pinppy**	**1**	**2**	**3**	**4**
Formula	$C_{18}H_{19}N_1$	$C_{58}H_{78}Ir_1N_7$	$C_{60}H_{81}I_1Ir_1N_9$	$C_{108}H_{146}Ir_2$ $La_2N_{16}O_{19}$	$C_{49}H_{60}Ir_1$ $La_1N_{10}O_9$
Formula weight / g·mol^{-1}	249.34	1065.47	1247.43	2632.60	1264.18
T / K	100(2)				
λ / Å	0.71073 (Mo Kα)				
Crystal system	monoclinic	orthorhombic	orthorhombic	hexagonal	orthorhombic
Space group	C 2	P $2_12_12_1$	P $2_12_12_1$	P 6_222	P $2_12_12_1$
a / Å	17.8175(6)	13.624(3)	9.5351(3)	17.3485(8)	14.5078(3)
b / Å	6.2588(2)	14.206(2)	18.9523(5)	17.3485(8)	16.0432(4)
c / Å	12.4934(5)	27.254(5)	30.5719(9)	37.291(3)	21.8900(4)
α / °	90	90	90	90	90
β / °	93.7720(10)	90	90	90	90
γ / °	90	90	90	120	90
V / Å^3	1390.20(9)	5275.0(16)	5524.7(3)	9719.8(11)	5094.93(19)
Z	4	4	4	3	4
Density / g·cm^{-1}	1.191	1.342	1.500	1.349	1.648
Absorption coefficient / cm^{-1}	0.069	2.575	3.023	2.755	3.501
F(000)	536	2208	2536	3972	2520
Θ range / °	2.901–27.127	2.554–25.027	2.25–25.027	2.411–25.691	2.253–26.368
Limiting indices	$-22 < h < 22$ $-8 < k < 8$ $-16 < l < 16$	$-16 < h < 16$ $-16 < k < 16$ $-32 < l < 32$	$-11 < h < 11$ $-22 < k < 22$ $-36 < l < 36$	$-21 < h < 21$ $-21 < k < 21$ $-45 < l < 45$	$-18 < h < 18$ $-20 < k < 19$ $-27 < l < 26$
Collected reflections	9428	61588	66980	147854	40778
R_{int}	0.0200	0.0455	0.0534	0.0452	0.0476
Completeness	0.996	0.997	0.998	0.998	0.995
Flack parameter	-	0.015(3)	0.016(4)	0.147(7)	0.056(4)
Data /parameters /restraints	3054/172/1	9286/0/597	9746/131/ 645	6170/122/358	10357/23/641
GOF on F^2	1.066	1.088	1.104	1.226	1.059
Final R indices (R_1 for [$I > 2\sigma(I)$] wR_2 for all data)	R_1 = 0.0391 wR_2 = 0.0995	R_1 = 0.0244 wR_2 = 0.0524	R_1 = 0.0403 wR_2 = 0.0979	R_1 = 0.1005 wR_2 = 0.2293	R_1 = 0.0352 wR_2 = 0.0678
Largest diff. peak and hole /e·Å^{-3}	0.263/−0.215	0.907/−1.087	0.85/−1.531	1.887/−2.424	1.503/−1.194

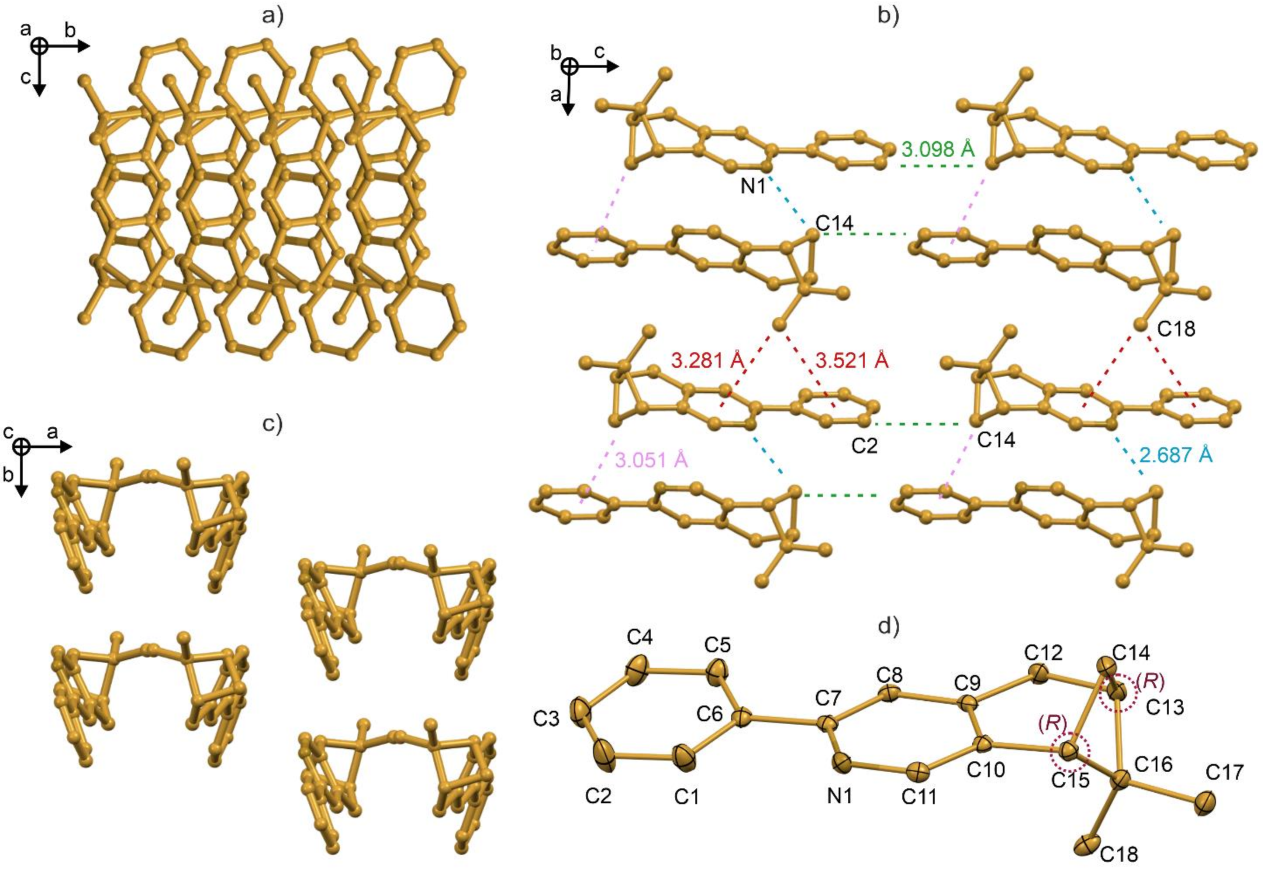

Figure S3. The views of the crystal structure of **(*R,R*)-pinppy** along the main *a*, *b*, and *c* crystallographic axes (a−c), and the asymmetric unit with the labeling scheme for symmetrically independent atoms (d). In (b), the C−H····· aromatic ring interactions between molecules are highlighted. Thermal ellipsoids were presented at a 40% probability level. Hydrogen atoms were omitted for clarity.

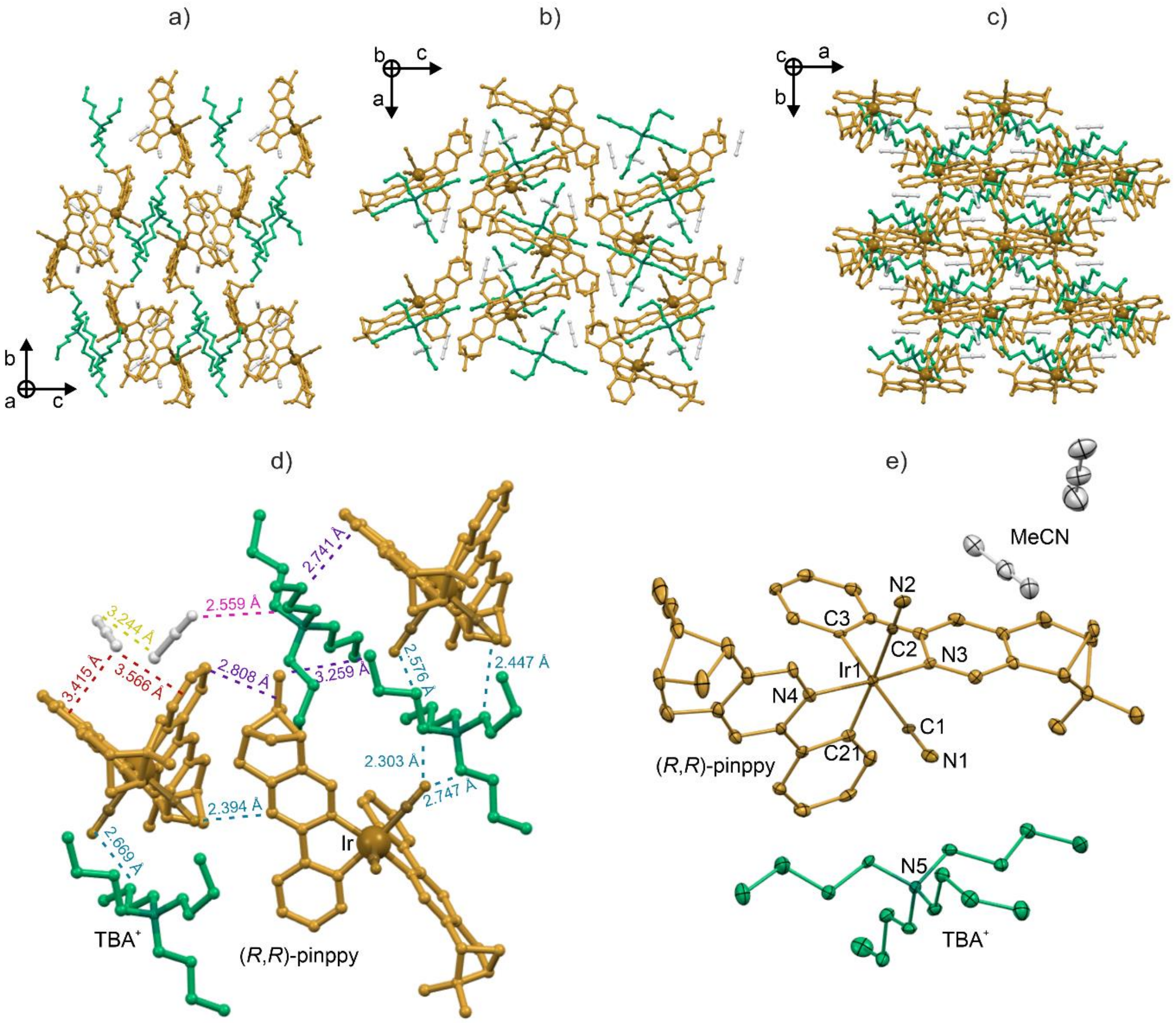

Figure S4. The views of the crystal structure of **1** along the main *a*, *b*, and *c* crystallographic axes (a−c), the interactions scheme between ions in the crystal structure (d), and the asymmetric unit with the labeling scheme for selected symmetrically independent atoms (e). Thermal ellipsoids were presented at a 40% probability level. Hydrogen atoms were omitted for clarity. Colors: yellow with various hues = Ir centers with cyanido and (*R*,*R*)-pinppy ligands attached to them, green = TBA$^+$ cations, grey = MeCN molecules.

Table S2. Detailed structure parameters of **1**.

Selected bond distances in *cis*-[Ir1(CN)$_2$(*R*,*R*-pinppy)$_2$]$^-$ complexes / Å					
Ir1−C1	2.033(5)	Ir1−C3	2.055(5)	Ir1−N3	2.055(4)
Ir1−C2	2.044(5)	Ir1−C21	2.050(5)	Ir1−N4	2.047(4)
Selected angles in *cis*-[Ir1(CN)$_2$(*R*,*R*-pinppy)$_2$]$^-$ complexes / °					
C1−Ir1−C2	88.1(2)	C2−Ir1−C3	94.61(19)	C3−Ir1−N3	79.92(18)
C1−Ir1−C3	174.8(2)	C2−Ir1−C21	173.6(2)	C3−Ir1−N4	91.8(2)
C1−Ir1−C21	89.5(2)	C2−Ir1−N3	90.29(19)	C21−Ir1−N3	95.87(18)
C1−Ir1−N3	95.72(18)	C2−Ir1−N4	94.19(18)	C21−Ir1−N4	79.96(18)
C1−Ir1−N4	92.4(2)	C3−Ir1−C21	88.2(2)	N3−Ir1−N4	170.89(17)

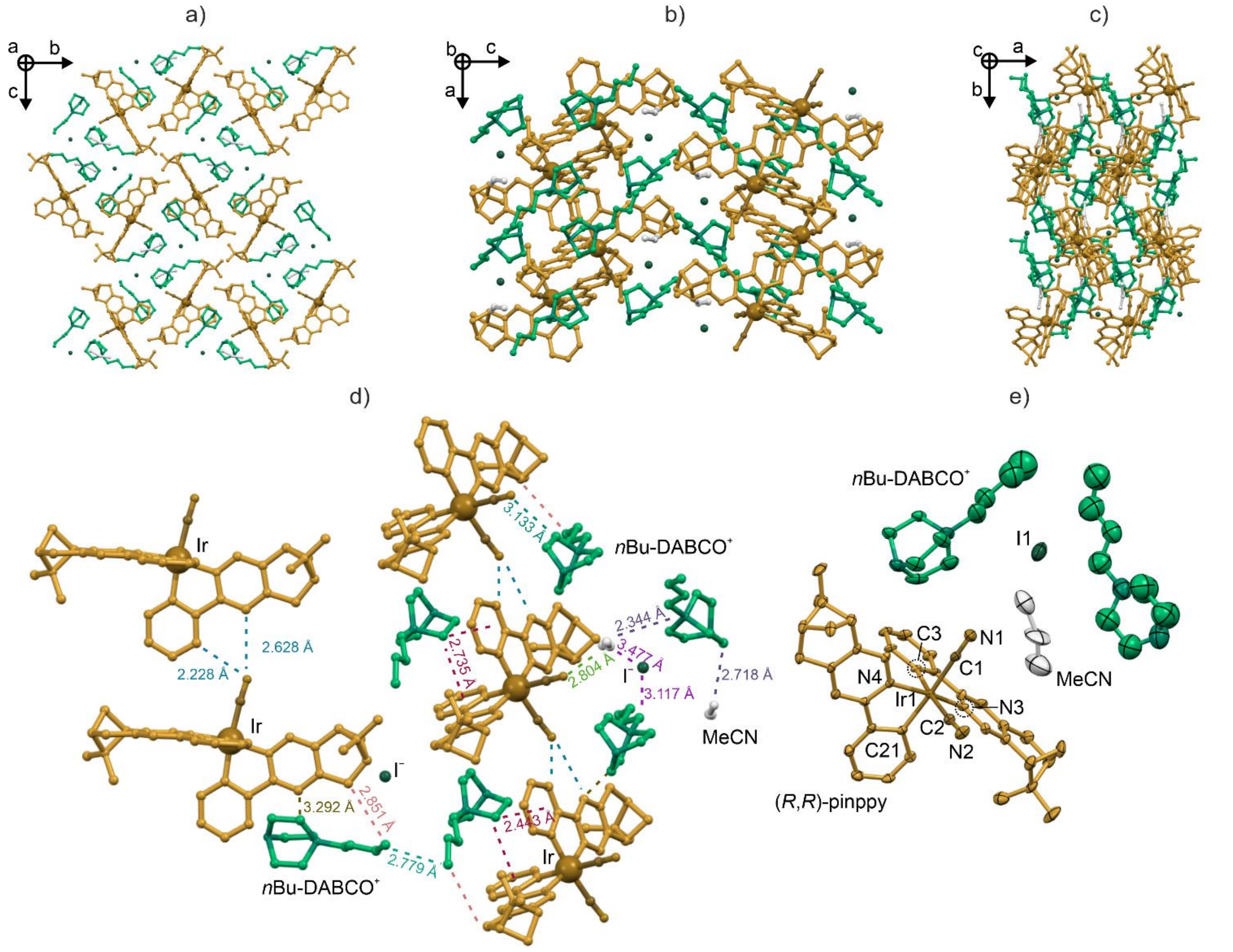

Figure S5. The views of the crystal structure of **2** along the main *a*, *b*, and *c* crystallographic axes (a–c), the interactions scheme between ions in the crystal structure (d), and the asymmetric unit with the labeling scheme for = symmetrically independent atoms (e). Thermal ellipsoids were presented at a 40% probability level. Hydrogen atoms were omitted for clarity. Colors: yellow with various hues = Ir centers with cyanido and (*R*,*R*)-pinppy ligands attached to them, dark green = iodide anions, light green = *n*-BuDABCO$^+$ cations, grey = MeCN.

Table S3. Detailed structure parameter of **2**.

Selected bond distances in *cis*-[Ir1(CN)$_2$(*R*,*R*-pinppy)$_2$]$^-$ complexes / Å					
Ir1−C1	2.048(10)	Ir1−C3	2.053(9)	Ir1−N3	2.045(8)
Ir1−C2	2.061(10)	Ir1−C21	2.041(9)	Ir1−N4	2.058(8)
Selected angles in *cis*-[Ir1(CN)$_2$(*R*,*R*-pinppy)$_2$]$^-$ complexes / °					
C1−Ir1−C2	88.5(4)	C2−Ir1−C3	173.2(4)	C3−Ir1−N3	79.7(4)
C1−Ir1−C3	88.8(4)	C2−Ir1−C21	92.4(4)	C3−Ir1−N4	91.4(3)
C1−Ir1−C21	176.5(4)	C2−Ir1−N3	94.1(4)	C21−Ir1−N3	90.9(3)
C1−Ir1−N3	92.4(4)	C2−Ir1−N4	95.2(3)	C21−Ir1−N4	79.6(3)
C1−Ir1−N4	97.0(4)	C3−Ir1−C21	90.6(4)	N3−Ir1−N4	167.0(3)

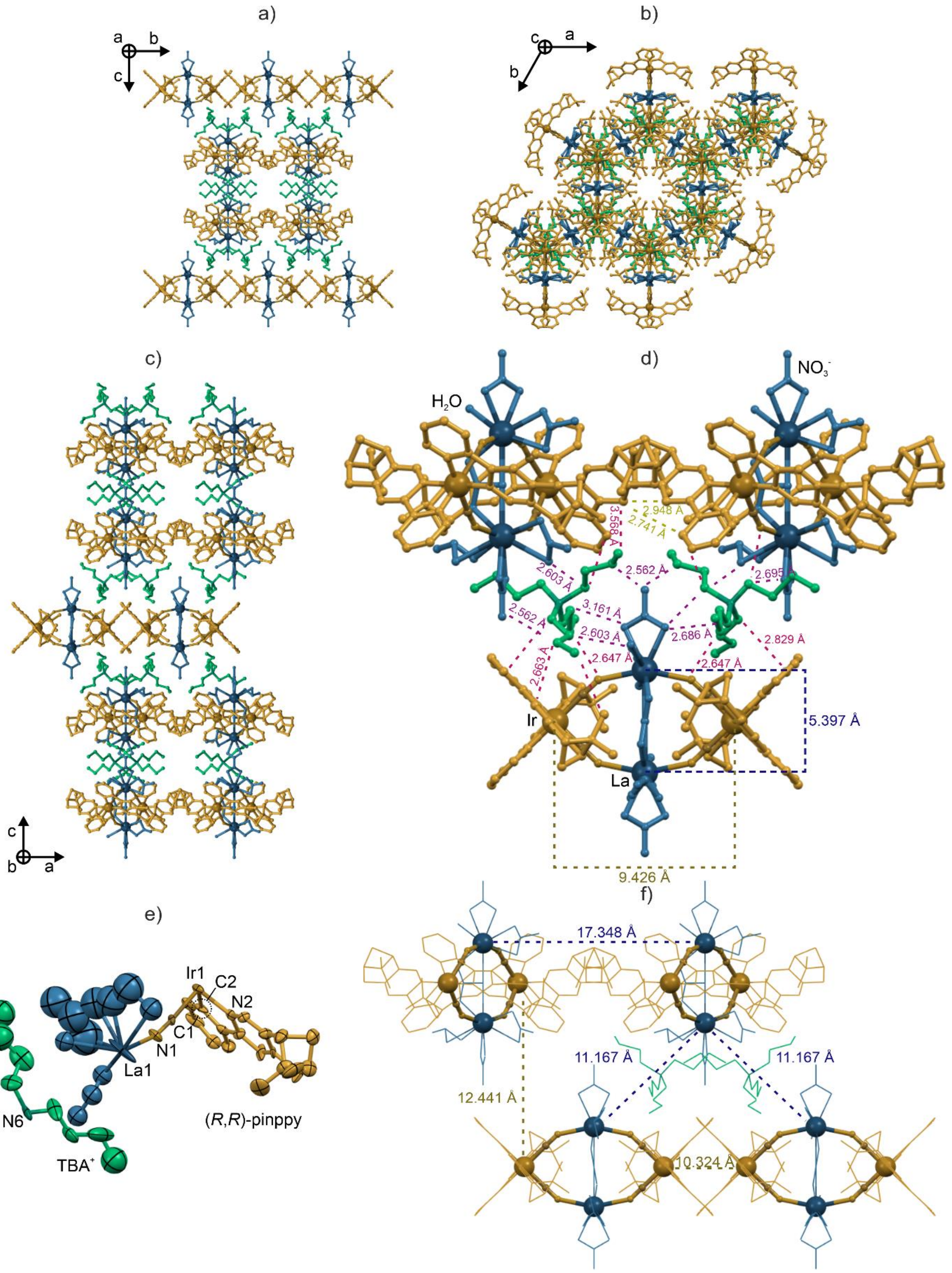

Figure S6. The views of the crystal structure of **3** along the main *a*, *b*, and *c* crystallographic axes (*a*−*c*), the interaction scheme between ions in the crystal structure (d), the asymmetric unit with the labeling scheme for symmetrically independent atoms (e), and the arrangement of cyanido-bridged $\{La^{III}_2Ir^{III}_2\}$ clusters, together with the schematic presentation of metal complexes forming the clusters (f). Thermal ellipsoids were presented at a 40% probability level. Hydrogen atoms were omitted for clarity. Colors: yellow with various hues = Ir centers with cyanido and (*R*,*R*)-pinppy ligands attached to them, blue with various hues = La centers with nitrate anions and water molecules attached to them, green = TBA^+ cations.

Table S4. Detailed structure parameters of **3**.

Selected bond distances and angles in *cis*-$[Ir1(\mu\text{-}CN)_2(R,R\text{-pinppy})_2]^-$ complexes / Å, °					
Ir1−C1	2.040(16)	Ir1−C2	2.052(17)	Ir1−N2	2.032(19)
C1−Ir1−C1	89.6(9)	C1−Ir1−N2	88.2(7)/ 95.7(8)	C2−Ir1−N2	82.7(10)/ 93.3(10)
C1−Ir1−C2	90.6(6)/ 178.4(11)	C2−Ir1−C2	89.2(10)	N2−Ir1−N2	174.4(8)
Selected bond distances and angles in $[La1(\mu\text{-}NC)_2(\kappa^2\text{-}NO_3)_3(\mu,\kappa^2\text{-}NO_3)]^{3-}$ and $[La1(\mu\text{-}NC)_2(\kappa^2\text{-}NO_3)_2(\mu,\kappa^2\text{-}NO_3)(H_2O)]^{2-}$ complexes / Å °					
La1−N1	2.515(17)	N1−La1−O7	82(5)/ 84(5)	O3−La1−O8	118(3)
La1−O1	2.52(3)	N1−La1−O8	78.4(4)	O4−La1−O4	124.4(1)
La1−O3	2.50(3)	O1−La1−O1	53.2(14)	O4−La1−O5	51.2(9)/ 156(2)
La1−O4	2.84(6)	O1−La1−O3	47.7(18)/ 81(5)	O4−La1−O7	69(3)/ 158(5)
La1−O5	2.63(3)	O1−La1−O4	63.0(16)/ 67.7(13)	O4−La1−O8	117.8(12)
La1−O7	2.43(6)	O1−La1−O5	93.5(18)/ 118.9(14)	O5−La1−O5	145(2)
La1−O8	2.6985(18)	O1−La1−O7	136(3)/ 113(2)	O5−La1−O7	20.4(13)/ 124.2(13)
N1−La1−N1	156.8(7)	O1−La1−O8	153.4(7)	O5−La1−O8	72.3(12)
N1−La1−O1	77.9(8)/ 124.7(8)	O3−La1−O3	124(7)	O7−La1−O7	103.8(12)
N1−La1−O3	85(5)/ 106(4)	O3−La1−O4	31(4)/ 115.1(17)	O7−La1−O8	51.9(6)
N1−La1−O4	76.6(14)/ 114.8(13)	O3−La1−O5	47(3)/ 165.9(19)	C1−N1−La1	147.72
N1−La1−O5	84(2)/ 88(2)	O3−La1−O7	67(3)/ 166(6)	-	-

Selected distances and angles between metal ions within the $\{La^{III}_2Ir^{III}_2\}$ clusters / Å °		Selected distances between metal ions between the $\{La^{III}_2Ir^{III}_2\}$ clusters / Å	
La1−La1	5.397	La1−La1 (in [021] and [02-1])	11.167
La1−Ir1	5.430/5.432	La1−La1 (in [100] and [010])	17.349
Ir1−Ir1	9.426	Ir1−Ir1 (in [001])	12.441
La1−Ir1−La1	59.59	-	-
Ir1−La1−Ir1	120.43	-	-

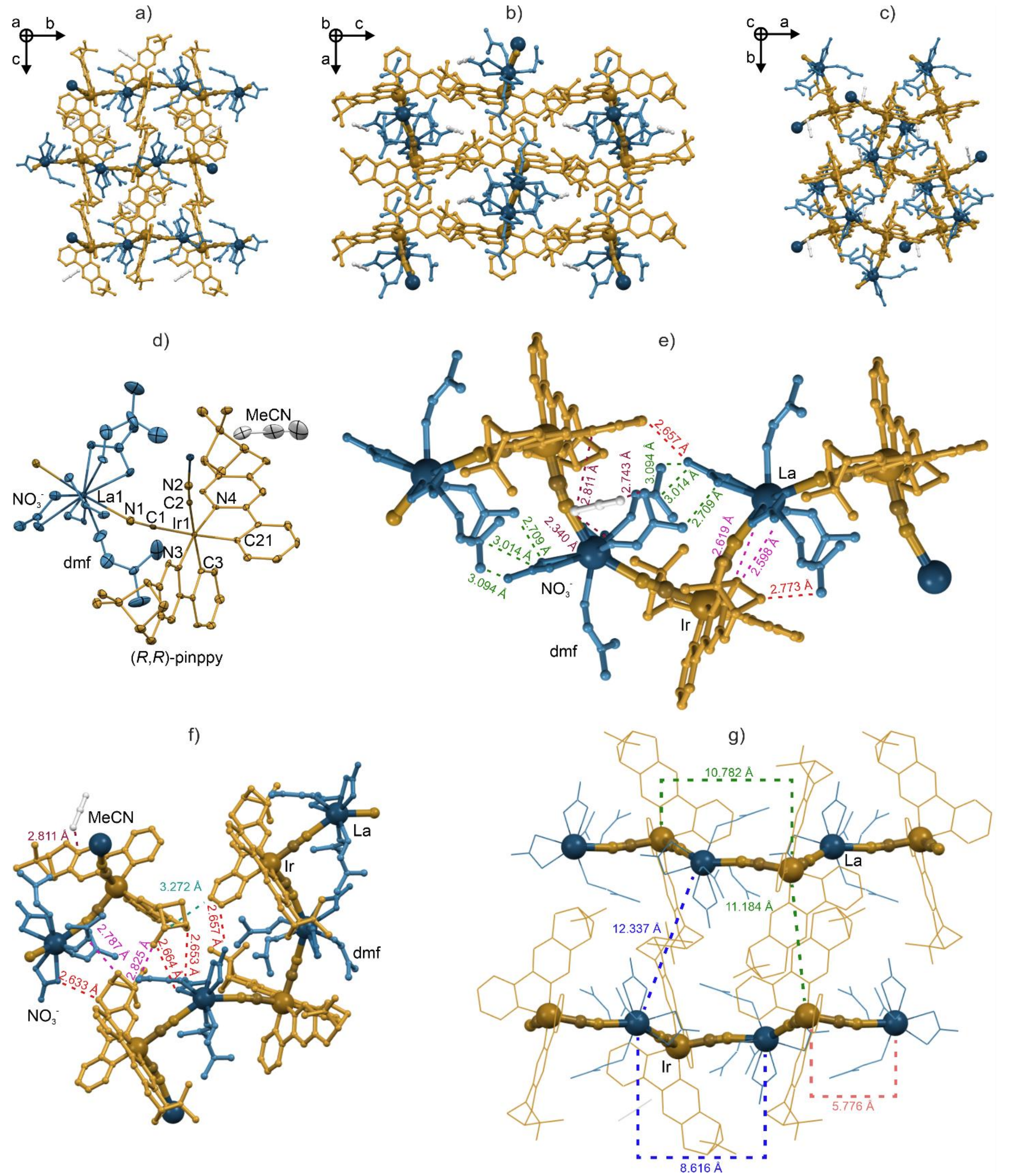

Figure S7. The view of the crystal structure of **4** along the main *a*, *b*, and *c* crystallographic axes (a–c), the asymmetric unit with the labeling scheme for symmetrically independent atoms (d), the interactions scheme between ions in the crystal structure (e,f*)*, the detailed insight into the intermetallic distances between neighboring metal centers within cyanido-bridged {$La^{III}Ir^{III}$} chains together with their arrangement in the crystal structure (g). Thermal ellipsoids were presented at a 40% probability. Hydrogen atoms were omitted for clarity. Colors: yellow with various hues = Ir centers with cyanido and (*R*,*R*)-pinppy ligands attached to them, blue with various hues = La centers with nitrate anions and dmf molecules attached to them, grey = MeCN molecules.

Table S5. Detailed structure parameters of compound **4**.

Selected bond distances and angles in *cis*-[Ir1(μ-CN)$_2$(*R*,*R*-pinppy)$_2$]$^-$ complexes / Å, °					
Ir1–C1	2.055(8)	C1–Ir1–C3	92.9(3)	C2–Ir1–N4	93.1(3)
Ir1–C2	2.044(7)	C1–Ir1–C21	174.5(3)	C3–Ir1–C21	86.7(3)
Ir1–C3	2.062(7)	C1–Ir1–N3	92.3(3)	C3–Ir1–N3	79.9(3)
Ir1–C21	2.051(8)	C1–Ir1–N4	94.6(3)	C3–Ir1–N4	92.3(3)
Ir1–N3	2.069(6)	C2–Ir1–C3	171.9(3)	C21–Ir1–N3	93.0(2)
Ir1–N4	2.053(6)	C2–Ir1–C21	88.2(3	C21–Ir1–N4	79.9(3)
C1–Ir1–C2	92.8(3)	C2–Ir1–N3	94.1(2)	N3–Ir1–N4	169.8(2)
Selected bond distances and angles in [La1(μ-NC)$_2$(κ^2-NO_3)$_2$(dmf)$_3$]$^-$ complexes / Å, °					
La1–N1	2.655(7)	N1–La1–O9	74.6(2)	O3–La1–O7	108.6(3)
La1–N2	2.629(7)	N2–La1–O1	121.70(19)	O3–La1–O8	142.5(2)
La1–O1	2.601(6)	N2–La1–O3	74.0(2)	O3–La1–O9	72.7(2)
La1–O3	2.624(6)	N2–La1–O4	75.7(2)	O4–La1–O5	49.14(18)
La1–O4	2.573(6)	N2–La1–O5	73.41(19)	O4–La1–O7	68.3(2)
La1–O5	2.604(6)	N2–La1–O7	139.6(2)	O4–La1–O8	127.49(19)
La1–O7	2.475(7)	N2–La1–O8	79.2(2)	O4–La1–O9	139.35(19)
La1–O8	2.443(5)	N2–La1–O9	79.0(2)	O5–La1–O7	69.5(2)
La1–O9	2.461(5)	O1–La1–O3	48.65(18)	O5–La1–O8	79.78(19)
N1–La1–N2	145.2(2)	O1–La1–O4	91.6(2)	O5–La1–O9	146.66(19)
N1–La1–O1	72.8(2)	O1–La1–O5	135.69(19)	O7–La1–O8	108.8(2)
N1–La1–O3	117.9(2)	O1–La1–O7	77.8(2)	O7–La1–O9	141.1(2)
N1–La1–O4	138.5(2)	O1–La1–O8	140.50(18)	O8–La1–O9	76.9(2)
N1–La1–O5	120.4(2)	O1–La1–O9	75.4(2)	C1–N1–La1	167.54
N1–La1–O7	70.8(2)	O3–La1–O4	70.03(19)	C2–N2–La1	167.77
N1–La1–O8	73.1(2)	O3–La1–O5	115.88(19)	-	-

Selected distances and angles between metal ions within the {La^{III}Ir^{III}} chains / Å, °		Selected distances between metal ions between the {La^{III}Ir^{III}} chains / Å	
La1–La1	8.616	the shortest La1–La1	12.337
La1–Ir1	5.776/5.819	the shortest Ir1–Ir1	11.184
Ir1–Ir1	10.782	-	-
La1–Ir1–La1	95.99	-	-
Ir1–La1–Ir1	136.84	-	-
Ir1–La1–Ir1–La1 (torsion)	3.04	-	-
La1–Ir1–La1–Ir1 (torsion)	1.44	-	-

Table S6. Results of Continuous Shape Measure (CShM) analysis for six-coordinated iridium(III) complexes in **1**–**4** and nine-coordinated lanthanum(III) complexes in **4**.

Comp.	Metal complex	CShM parameter*					Geom.
		HP-6	PPY-6	OC-6	TPR-6	JPPY-6	
**1**	*cis*-[Ir1(μ-CN)$_2${*trans*-(*R*,*R*-pinppy)$_2$}]$^-$	29.42	26.88	**0.54**	14.03	30.29	**OC-6**
**2**	*cis*-[Ir1(μ-CN)$_2${*trans*-(*R*,*R*-pinppy)$_2$}]$^-$	30.89	26.50	**0.60**	13.82	30.13	**OC-6**
**3**	*cis*-[Ir1(μ-CN)$_2${*trans*-(*R*,*R*-pinppy)$_2$}]$^-$	29.67	28.59	**0.28**	15.36	31.80	**OC-6**
**4**	*cis*-[Ir1(μ-CN)$_2${*trans*-(*R*,*R*-pinppy)$_2$}]$^-$	30.64	27.01	**0.48**	14.63	30.79	**OC-6**
**-**	-	JCSAPR-9	CSAPR-9	JTCTPR-9	TCTPR-9	MFF-9	**-**
**4**	[La1(μ-NC)$_2$(κ^2-NO$_3$)$_2$(dmf)$_3$]$^-$	2.78	1.92	3.67	2.96	**1.62**	**MFF-9**

Comment on Table S6: Continuous Shape Measure (CShM) analysis for Ir(III) and Ln(III) complexes was performed using SHAPE software ver. 2.1.21.[S11] The Continuous Shape Measure (CShM) parameter represents the distortion from an ideal geometry. It equals 0 for an ideal polyhedron and increases with increasing distortion. Due to the significant disorder in the crystal structure of **3** (see Experimental Details), some DFIX restraints were applied for the La−O bond distances to ensure the convergence of the refinement procedure. Therefore, the CShM analysis is burdened with a large experimental error. However, for the record, the best geometries for the nine-coordinated lanthanum(III) complexes in **3** are also found as MFF-9 (C_s), while the ten-coordinated complexes are best characterized by the geometry of JSPC-10 (sphenocorona, C_{2v}).

*Continuous Shape Measure (CShM) parameters[S12]

- for six-coordinated complexes:

CShM HP-6 − the parameter related to the hexagon (D_{6h} symmetry)

CShM PPY-6 − the parameter related to the pentagonal pyramid (C_{5v})

CShM OC-6 − the parameter related to the octahedron (O_h)

CShM TPR-6 − the parameter related to the trigonal prism (D_{3h})

CShM JPPY-6 − the parameter related to the Johnson pentagonal pyramid (C_{5v})

- for nine-coordinated complexes:[S13]

CShM JCSAPR-9 − the parameter related to the capped square antiprism J10 (C_{4v})

CShM CSAPR-9 − the parameter related to the spherical capped square antiprism (C_{4v})

CShM JTCTPR-9 − the parameter related to the tricapped trigonal prism J51 (D_{3h})

CShM TCTPR-9 − the parameter related to the spherical tricapped trigonal prism (D_{3h})

CShM MFF-9 − the parameter related to the muffin (C_s)

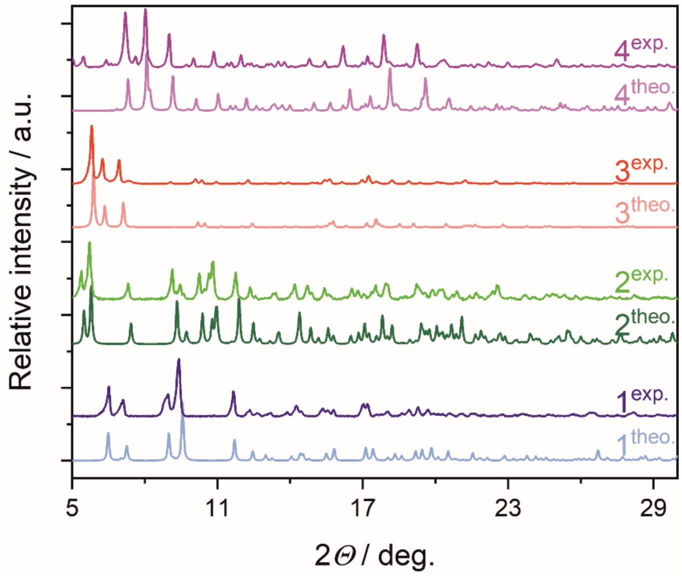

Figure S8. Comparison of experimental (exp.) and theoretical (theo.) powder X-ray diffraction (P-XRD) patterns of compounds **1**–**4**, presented in the 2Θ range of 5–30°. Theoretical P-XRD patterns were obtained from the single-crystal X-ray diffraction (SC-XRD) structural analysis (T = 100 K).

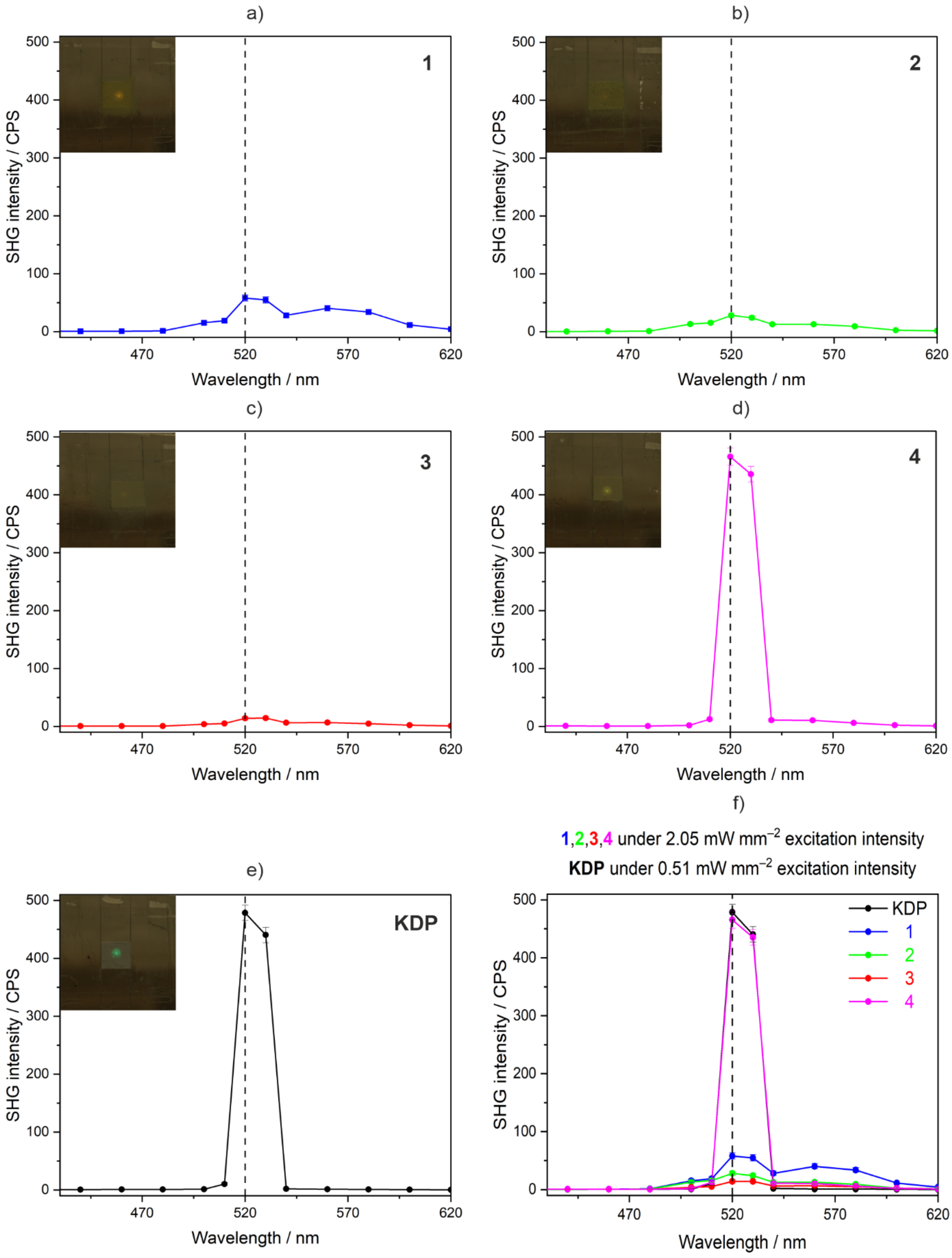

Figure S9. Wavelength dependences of the SH signal for the powder samples **1** (a), **2** (b), **3** (c), and **4** (d), and potassium dihydrogen phosphate (**KDP**), used as reference material (e), shown together with the comparison of the SH signals for all samples and the KDP reference (f). Insets in (a–e): the photos of respective samples, mounted within the setup of the SHG experiment under the 1040 nm laser light irradiation.

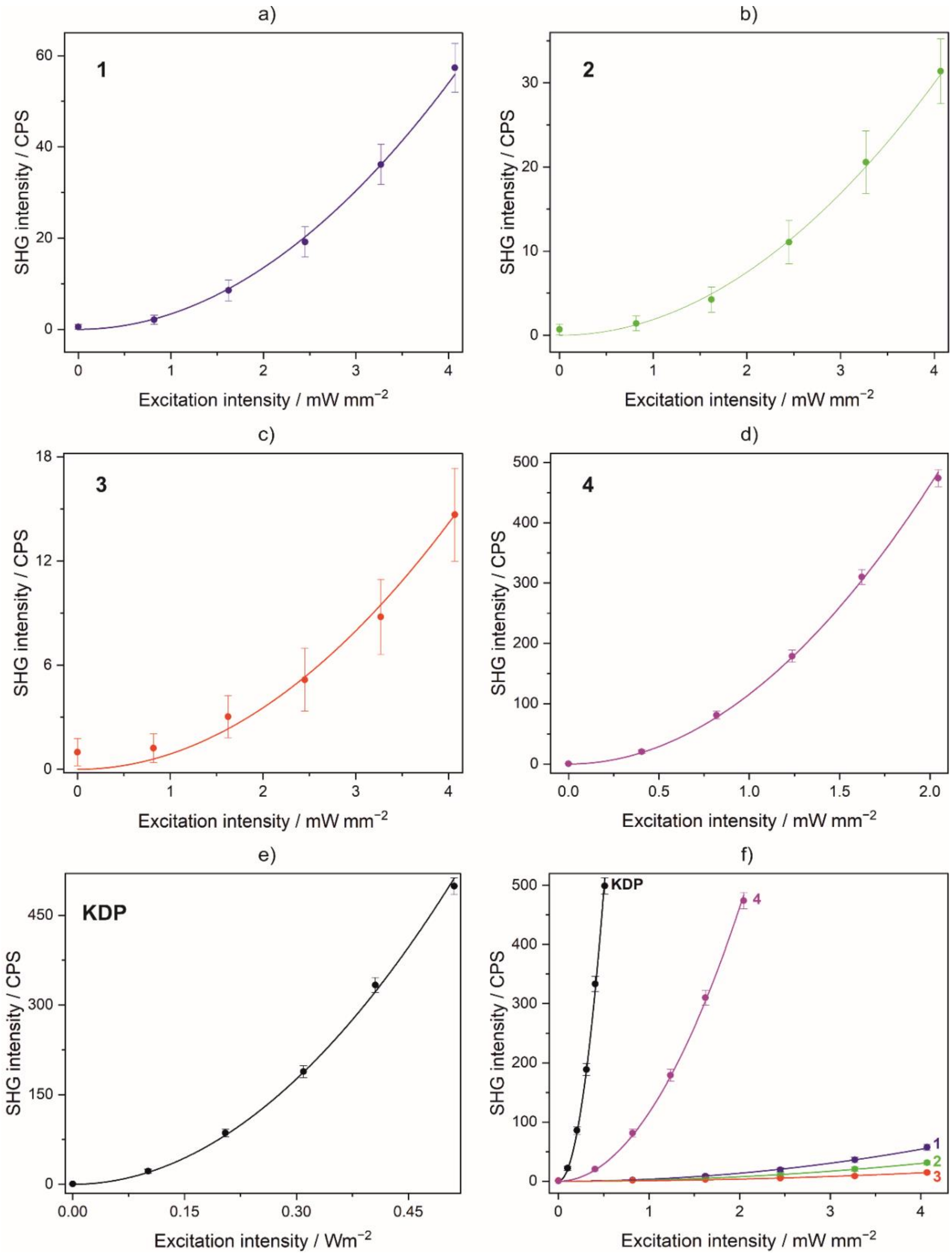

Figure S10. Dependences of the SH effect on the excitation intensity for the powder samples of **1** (a), **2** (b), **3** (c), and **4** (d), as well as potassium dihydrogen phosphate (**KDP**), used as a reference material (e), shown together with the comparison of all measured samples (f). Points represent measured data with intensity uncertainties. Lines correspond to best-fit curves to the quadratic equation pointing to the SH nature of emitted light.

Comment on the SHG effect shown in Figures S9 and S10: According to the Kleinman symmetry in non-dispersive materials, the SHG effect should not be observed in the *422*, *432*, and *622* point groups.[S14,S15] Thus, we suspected that the weak but non-negligible signal obtained for the powder sample of **3** could be connected with the contamination with the precursor or other molecular material; however, the P-XRD analysis (Fig. S8) showed that this sample is homogenous and does not contain any crystalline impurities. After further literature research, we were able to confirm that Kleinman symmetry is often violated for molecular materials apart from the quality of the crystal sample.[S16–S18] Therefore, we suppose that the results obtained for **3** are reliable.

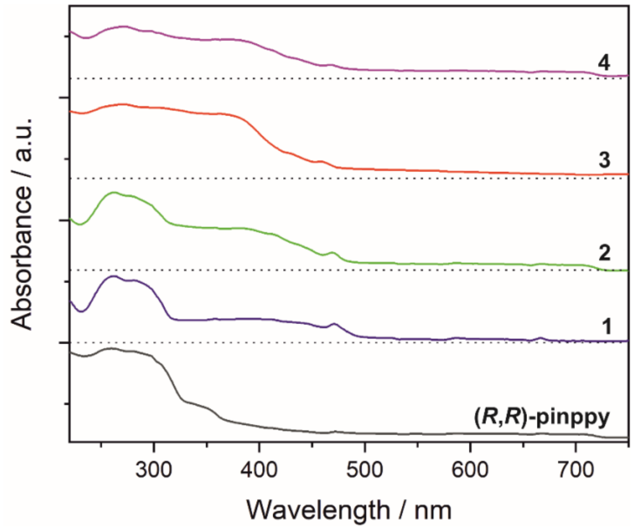

Figure S11. Solid-state UV-vis absorption spectra for **1**–**4** and **(*R,R*)-pinppy** ligand, gathered in the 220−750 nm wavelength range.

Comment on Figure S11: All obtained solids exhibit strong absorption in UV and visible range (up to ca. 400–480 nm) which is responsible for their yellow color. The spectra of **1** and **2** consist of two main bands, positioned in the range of 220–320 nm and 320–480 nm. The very broad band located in the visible range corresponds to metal-to-ligand charge transfer transitions (Ir^{III} to (*R,R*)-pinppy, MLCT). The second band, positioned in the UV region, corresponds to the $\pi \rightarrow \pi^*$ electronic transitions within the **(*R,R*)-pinppy** ligands, the higher energy charge-transfer transitions, and high energy d-d electronic transitions related to Ir metal centers. These bands are also detected in the absorption spectra of **3** and **4**; however, the strong MLCT bands are shifted to lower wavelengths, which might be correlated with the presence of heavy lanthanum(3+) ions to which the $[Ir(CN)_2(R,R\text{-pinppy})_2]^-$ metalloligands are coordinated.

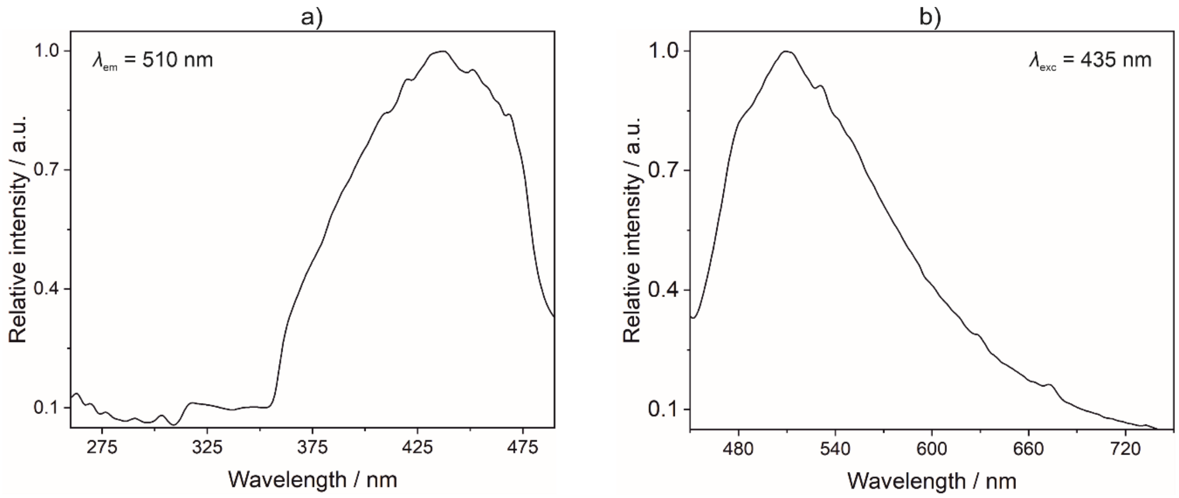

Figure S12. Solid-state excitation (a) and emission (b) of ***(R,R)*-pinppy** ligand gathered for the indicated emission and excitation wavelengths, respectively, at 77 K.

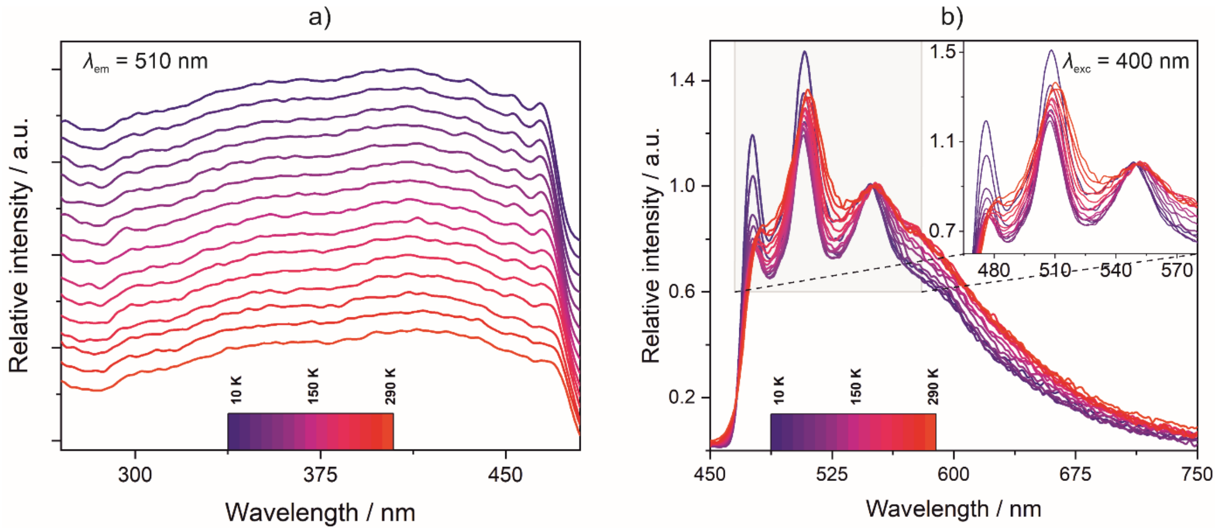

Figure S13. Temperature-variable solid-state excitation (a) and emission (b, normalized) spectra of **1**. The inset in (b) represents the enlargement of the 465−580 nm region of the emission spectrum. The related spectroscopic parameters of the emission patterns at each temperature are gathered in Table S7. The emission spectra in the (b) part were normalized to the third (going from lower to higher wavelengths) emission peak.

Table S7. The positions of well-distinguished maxima of the solid-state emission patterns of **1** detected at various indicated temperatures (the position of the main maximum at each temperature was underlined), shown together with the chromaticity parameters of the CIE 1931 scale (Fig. S13).

T / K	$\lambda_{em,max}$ / nm	CIE 1931 chromaticity parameters		*T* / K	$\lambda_{em,max}$ / nm	CIE 1931 chromaticity parameters	
		x	*y*			*x*	*y*
10	476, 508, 548.5	0.327	0.502	170	477.5, 508, 550	0.362	0.505
30	476.5, 507.5, 550	0.334	0.506	190	478, 507.5, 550.5	0.363	0.505
50	476, 508, 548.5	0.343	0.507	210	479, 508.5, 551.5	0.363	0.505
70	476.5, 507.5, 549	0.348	0.507	230	479.5, 509, 549	0.363	0.506
90	467, 507.5, 550.5	0.352	0.507	250	481.5, 510, 549.5	0.356	0.508
110	476, 507, 551	0.356	0.506	270	482, 511,550	0.356	0.509
130	477, 507, 550.5	0.359	0.505	290	482.5, 510, 552	0.354	0.510
150	477, 507.5, 551	0.361	0.505	-	-	-	-

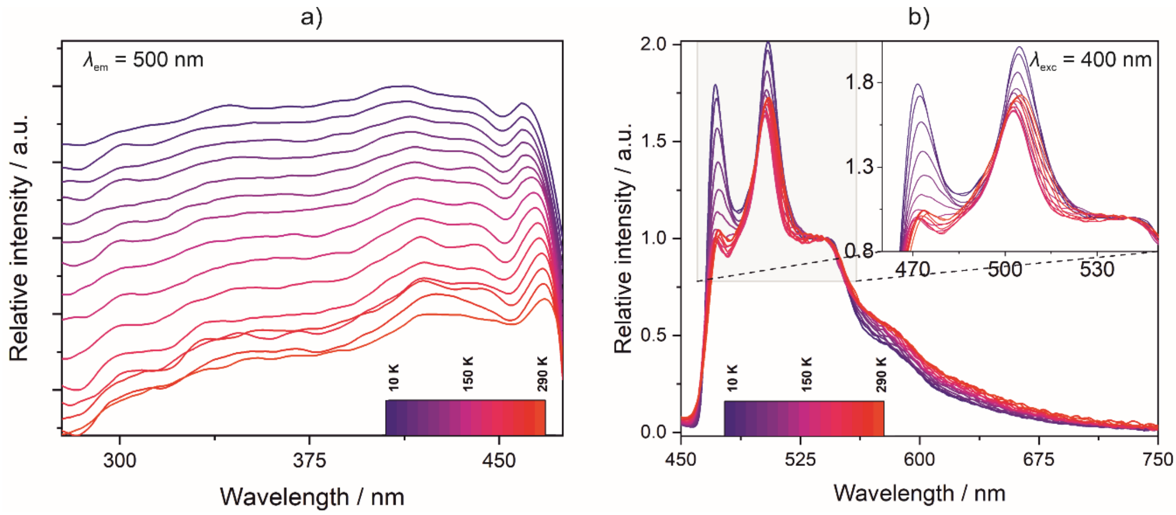

Figure S14. Temperature-variable solid-state excitation (a) and emission (b, normalized) spectra of **2**. The inset in (b) represents the enlargement of the 465−550 nm region of the emission spectrum. The related spectroscopic parameters of the emission patterns at each temperature are gathered in Table S8. The emission spectra in the (b) part were normalized to the third (going from lower to higher wavelengths) emission peak.

Table S8. The positions of well-distinguished maxima of the solid-state emission patterns of **2** detected at various indicated temperatures (the position of the main maximum at each temperature was underlined), shown together with the chromaticity parameters of the CIE 1931 scale (Fig. S14).

T / K	$\lambda_{em,max}$ / nm	CIE 1931 chromaticity parameters		*T* / K	$\lambda_{em,max}$ / nm	CIE 1931 chromaticity parameters	
		x	*y*			*x*	*y*
10	471.5, <u>504.5</u>, 539	0.240	0.473	170	471, <u>502.5</u>, 534.5	0.276	0.493
30	472, <u>504.5</u>, 541	0.243	0.477	190	472, <u>503</u>, 541	0.278	0.492
50	472.5, <u>504</u>, 542	0.248	0.481	210	472.5, <u>503</u>, 540.5	0.280	0.491
70	473, <u>503.5</u>, 540	0.254	0.486	230	472.5, <u>503.5</u>, 538.5	0.281	0.491
90	473.5, <u>503</u>, 541.5	0.260	0.488	250	473.5, <u>504</u>, 542	0.280	0.493
110	472.5, <u>502.5</u>, 541.5	0.266	0.490	270	474, <u>504.5</u>, 540.5	0.280	0.493
130	471.5, <u>502.5</u>, 539	0.270	0.492	290	475, <u>505</u>, 538.5	0.279	0.498
150	471.5, <u>502.5</u>, 540	0.272	0.493	-	-	-	-

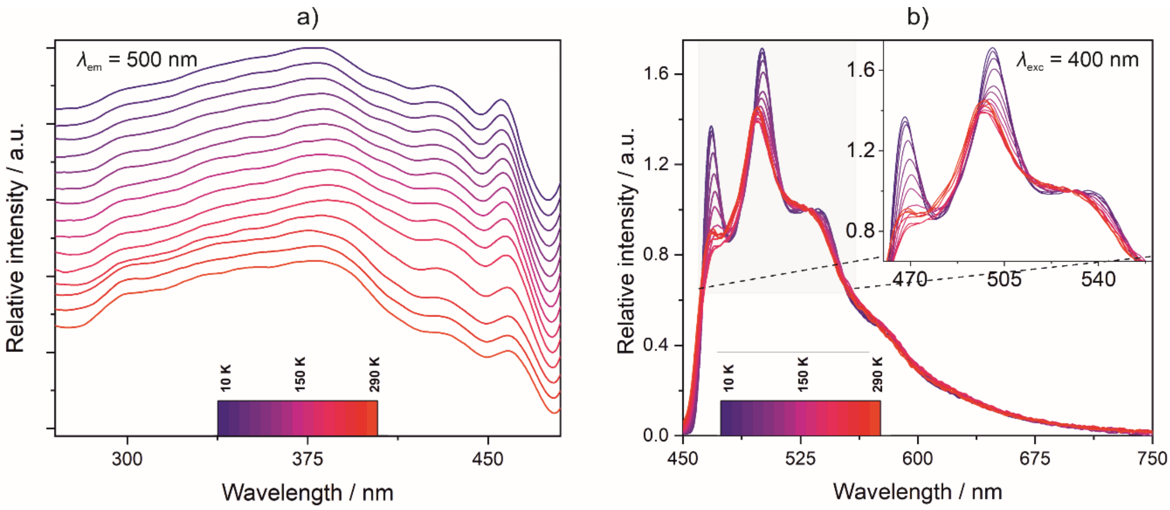

Figure S15. Temperature-variable solid-state excitation (a) and emission (b, normalized) spectra of **3**. The inset in (b) represents the enlargement of the 460−560 nm region of the emission spectrum. The related spectroscopic parameters of the emission patterns at each temperature are gathered in Table S9. The emission spectra in the (b) part were normalized to the third (going from lower to higher wavelengths) emission peak.

Table S9. The positions of well-distinguished maxima of the solid-state emission patterns of **3** detected at various indicated temperatures (the position of the main maximum at each temperature was underlined), shown together with the chromaticity parameters of the CIE 1931 scale (Fig. S15).

T / K	$\lambda_{em,max}$ / nm	CIE 1931 chromaticity parameters		*T* / K	$\lambda_{em,max}$ / nm	CIE 1931 chromaticity parameters	
		x	*y*			*x*	*y*
10	468, 500.5, 527	0.249	0.463	170	470.5, 497.5, 530.5	0.263	0.476
30	468, 500.5, 528.5	0.250	0.465	190	470, 497, 531	0.264	0.476
50	468.5, 501, 528	0.251	0.467	210	471, 496.5, 531	0.263	0.477
70	469, 501, 532	0.254	0.471	230	471, 497, 532.5	0.258	0.468
90	469.5, 501, 529	0.256	0.472	250	469.5, 496.5, 529	0.253	0.462
110	470.5, 500, 529	0.257	0.475	270	469, 498, 526.5	0.252	0.460
130	470, 499.5, 527.5	0.260	0.474	290	469, 497.5, 529	0.252	0.459
150	472, 499.5, 531.5	0.262	0.476	-	-	-	-

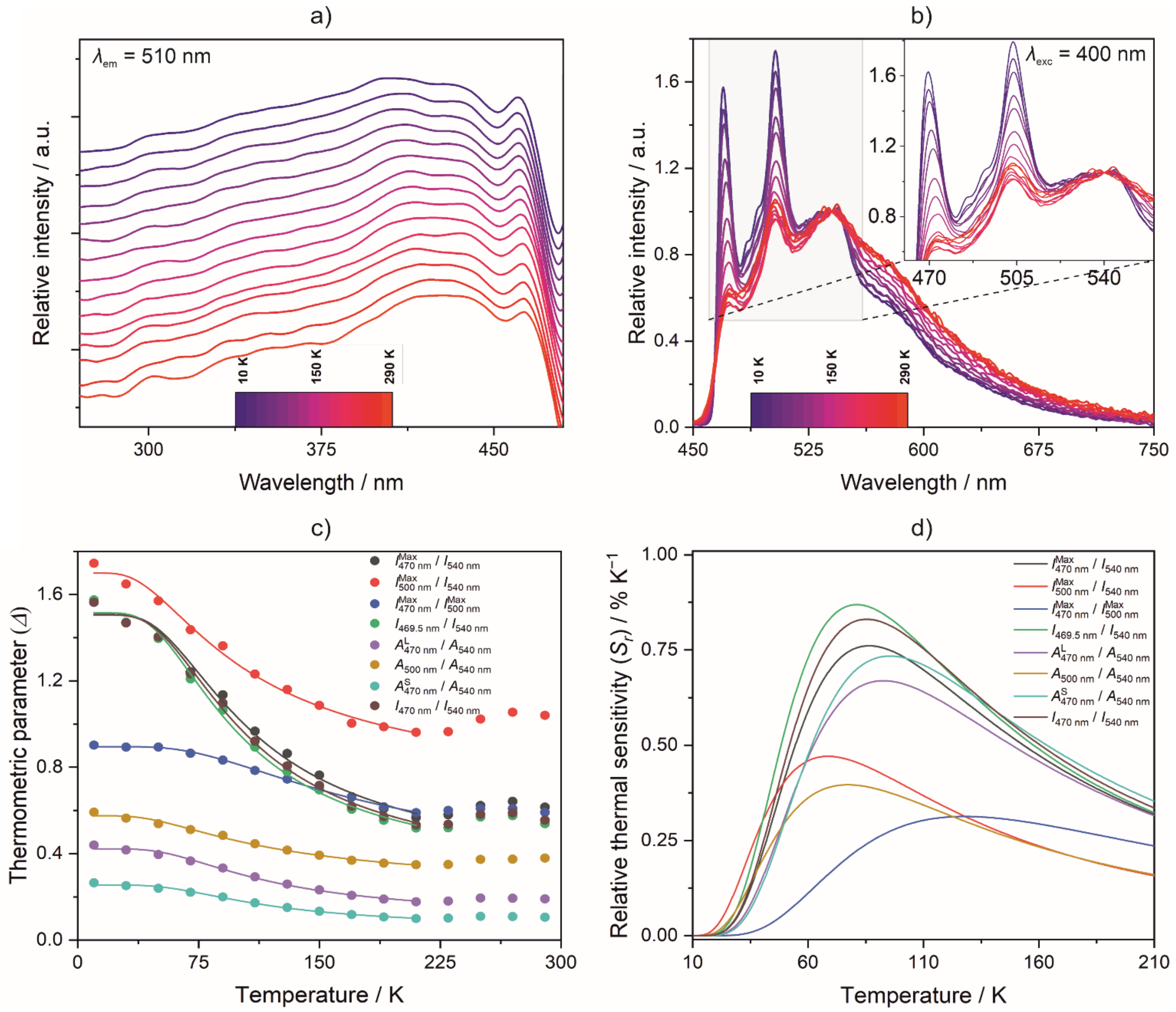

Figure S16. Temperature-variable solid-state excitation (a) and emission (b, normalized) spectra of **4**, shown together with the comparison of a few different indicated thermometric parameters (c), and the corresponding relative thermal sensitivity curves (d). The inset in (b) represents the enlargement of the 465−560 nm region of the emission spectrum. The related spectroscopic parameters of the emission patterns at each temperature are gathered in Tables S10 and S11. See the comment below for details. The emission spectra in the (b) part were normalized to the third (going from lower to higher wavelengths) emission peak.

Table S10. The positions of well-distinguished maxima of the solid-state emission patterns of **4** detected at various indicated temperatures (the position of the main maximum at each temperature was underlined), shown together with the chromaticity parameters of the CIE 1931 scale (Fig. S16).

T / K	$\lambda_{em,max}$ / nm	CIE 1931 chromaticity parameters		T / K	$\lambda_{em,max}$ / nm	CIE 1931 chromaticity parameters	
		x	y			x	y
10	469.5, 503.5, 542	0.282	0.482	170	473.5, 503, 540.5	0.342	0.506
30	469.5, 503.5, 541	0.285	0.486	190	472.5, 503, 542.5	0.348	0.508
50	470, 503.5, 543.5	0.291	0.487	210	474, 504, 542.5	0.353	0.508
70	470.5, 503.5, 540.5	0.297	0.492	230	474, 503, 541	0.351	0.507
90	471.5, 504, 543.5	0.305	0.496	250	473, 502.5, 543.5	0.345	0.503
110	472, 504, 540.5	0.315	0.500	270	473.5, 503.5, 543.5	0.344	0.502
130	472.5, 504, 539	0.323	0.506	290	473, 503.5, 537.5	0.345	0.503
150	473.5, 502.5, 538	0.332	0.504	-	-	-	-

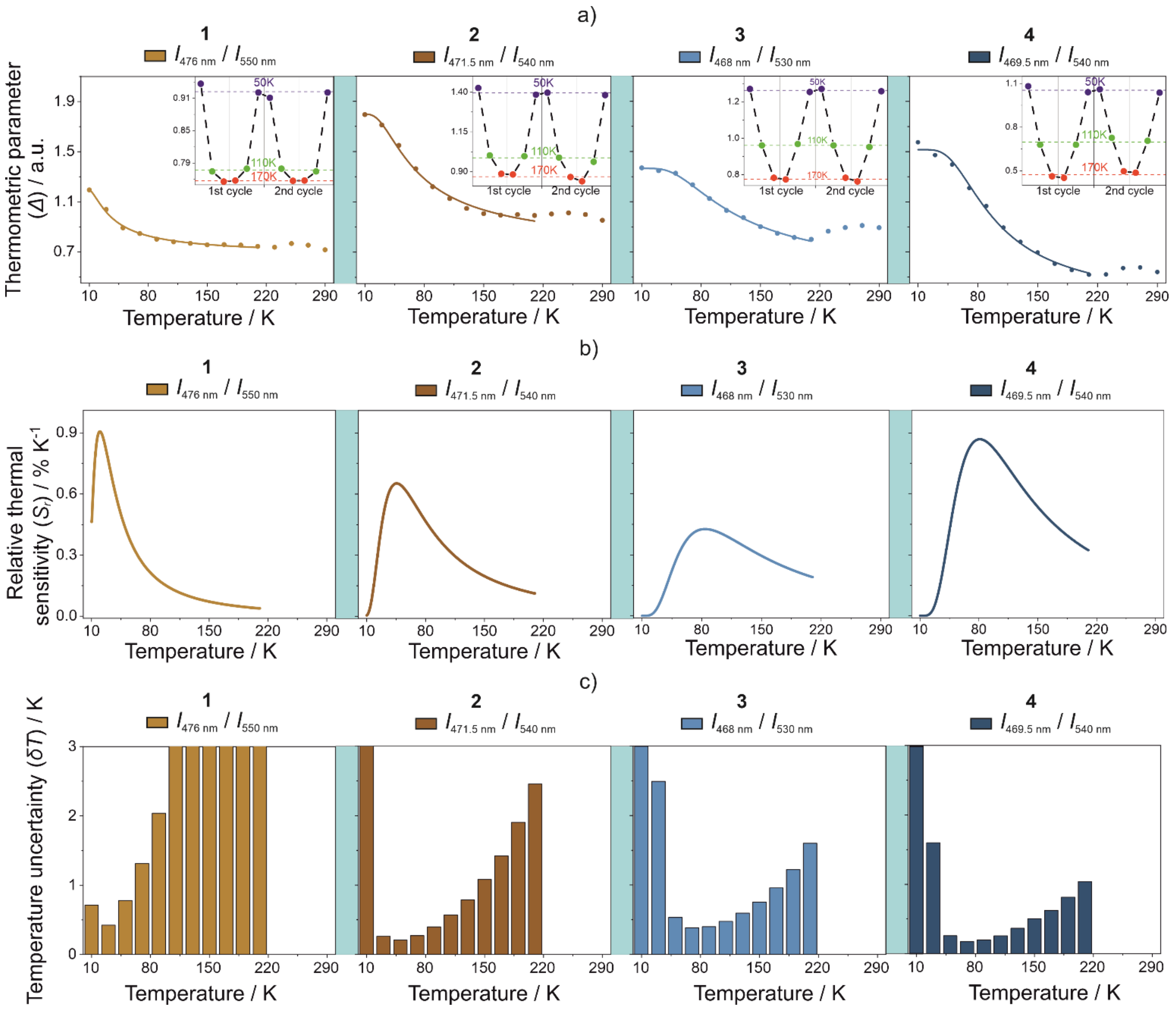

Figure S17. Selected optical thermometry characteristics for compounds **1–4**. The (a) part contains the best thermometric parameters obtained for all the compounds; in the inset of (a), the repeatability of the optical parameters is presented. Panel (b) contains the relative thermal sensitivity curves calculated based on experimental thermometric parameters shown in (a). The last panel, (c) contains the temperature uncertainty at indicated temperature ranges; this part was also calculated using the best thermometric parameter characteristics shown in (a). See the comment below for details.

Comment on Figures S16 and S17:

In the quest for the development of the thermometric parameter that most accurately enables temperature detection, a few different parameters were defined. This is presented precisely for compound **4** (Figure S16) while, for other compounds, only the optimized thermometric parameter is discussed (Figure S17).

For the case of **4** (Figure S16), the first approach was based on the intensity at maximum, in such a way five thermometric parameters were defined, namely, $I_{470\,\mathrm{nm}}^{\mathrm{Max}}/I_{540\,\mathrm{nm}}$, $I_{500\,\mathrm{nm}}^{\mathrm{Max}}/I_{540\,\mathrm{nm}}$, $I_{470\,\mathrm{nm}}^{\mathrm{Max}}/I_{500\,\mathrm{nm}}^{\mathrm{Max}}$, $I_{469.5\,\mathrm{nm}}/I_{540\,\mathrm{nm}}$, $I_{470\,\mathrm{nm}}/I_{540\,\mathrm{nm}}$ (where $I_{470\,\mathrm{nm}}^{\mathrm{Max}}$ is the intensity at the maximal value for the peak around 470 nm, $I_{540\,\mathrm{nm}}$ is the intensity at the maximum at 540 nm which does not change its position upon cooling/heating, $I_{500\,\mathrm{nm}}^{\mathrm{Max}}$ is the intensity at the maximal value for the peak around 500 nm, $I_{469.5\,\mathrm{nm}}$ is the intensity at 469.5 nm, $I_{470\,\mathrm{nm}}$ is the intensity at 470 nm).

The second approach was based on the ratios of areas under the emission bands, and similarly to the first approach, three thermometric parameters were defined. For the band at around 470 nm two different ranges for integration were taken: 466.5–477.5 nm (11 nm, larger, $A^{L}_{470\,\mathrm{nm}}$) and 468.5–474.5 nm (6 nm, smaller, $A^{S}_{470\,\mathrm{nm}}$). For the band at around 500 nm, the integration range was 497.5nm–510 nm ($A_{500\,\mathrm{nm}}$), while for the band at around 540 nm, the integration range was 525.5–560.5 nm ($A_{540\,\mathrm{nm}}$). Therefore, three respective thermometric parameters were as follows: $A_{470\,\mathrm{nm}}^{\mathrm{L}}/A_{540\,\mathrm{nm}}$, $A_{500\,\mathrm{nm}}/A_{540\,\mathrm{nm}}$, and $A_{470\,\mathrm{nm}}^{\mathrm{S}}/A_{540\,\mathrm{nm}}$ (Figure S16 c).

For all of the temperature-dependent thermometric parameters *Δ*, a classical Mott-Seitz model was applied, under the assumption of the existence of only one nonradiative channel of emission extinction:

$$\Delta(T) = \frac{\Delta_0}{1 + \alpha\, exp\left(\frac{\Delta E}{k_B T}\right)}$$

where:

Δ_0 = thermometric parameter at 0 K,

$\alpha = \frac{W_0}{W_R}$ is the ratio of non-radiative (W_0 at 0 K) and radiative (W_R) rates of decay,

ΔE = activation energy of non-radiative channel of decay.

All parameters obtained from the fitting of the function to gathered data points are presented in Table S11. To compare the performance of different thermometer parameters, a relative thermal sensitivity (S_r), derived from fitted curves, was used. We followed the equation:

$$S_r = \frac{\left|\frac{\partial \Delta}{\partial T}\right|}{\Delta}$$

Then, temperature uncertainty was calculated by the following equation:[S19]

$$\delta T = \left(\frac{\delta\Delta}{\Delta}\right)\left(\frac{1}{Sr}\right) = \left(\sqrt{\left(\frac{\delta_{DET}}{I_1}\right)^2 + \left(\frac{\delta_{DET}}{I_2}\right)^2}\right)\left(\frac{1}{Sr}\right)$$

where:

$\frac{\delta\Delta}{\Delta}$ is the uncertainty of the thermometric parameter, S_r is the relative thermal sensitivity, δ_{DET} in the uncertainty of the detector equal to 2 CPS (value determined based on standard deviation of the noise related to the detector, I_1 and I_2 are the values of the intensity at specific points in a non-normalized, non-smoothed emission spectrum.

Table S11. Comparison of best-fit parameters to the thermometric calibration curves for **1–4** (see Fig. S16 and S17, and related comment for details).

Compound	Thermometric parameter	Δ_0	α	$\frac{\Delta E}{k_B}$ / K
**1**	$I_{476\ \mathrm{nm}}/I_{550\ \mathrm{nm}}$	1.21(1)	0.79(2)	43(3)
**2**	$I_{471.5\ \mathrm{nm}}/I_{540\ \mathrm{nm}}$	1.80(2)	1.48(8)	105(8)
**3**	$I_{468\ \mathrm{nm}}/I_{530\ \mathrm{nm}}$	1.356(7)	1.89(8)	198(7)
**4**	$I^{\mathrm{Max}}_{470\ \mathrm{nm}}/I_{540\ \mathrm{nm}}$	1.51(2)	4.69(59)	230(18)
	$I^{\mathrm{Max}}_{500\ \mathrm{nm}}/I_{540\ \mathrm{nm}}$	1.70(2)	1.65(13)	159(12)
	$I^{\mathrm{Max}}_{500\ \mathrm{nm}}/I_{540\ \mathrm{nm}}$	0.896(3)	2.20(14)	307(11)
	$A^{L}_{470\ \mathrm{nm}}/A_{540\ \mathrm{nm}}$	0.423(6)	4.25(49)	242(18)
	$A_{500\ \mathrm{nm}}/A_{540\ \mathrm{nm}}$	0.575(6)	1.53(14)	178(15)
	$A^{S}_{470\ \mathrm{nm}}/A_{540\ \mathrm{nm}}$	0.255(3)	5.21(60)	258(17)
	$I_{469.5\ \mathrm{nm}}/I_{540\ \mathrm{nm}}$	1.52(2)	5.26(51)	219(14)
	$I_{470\ \mathrm{nm}}/I_{540\ \mathrm{nm}}$	1.51(2)	5.33(54)	231(14)

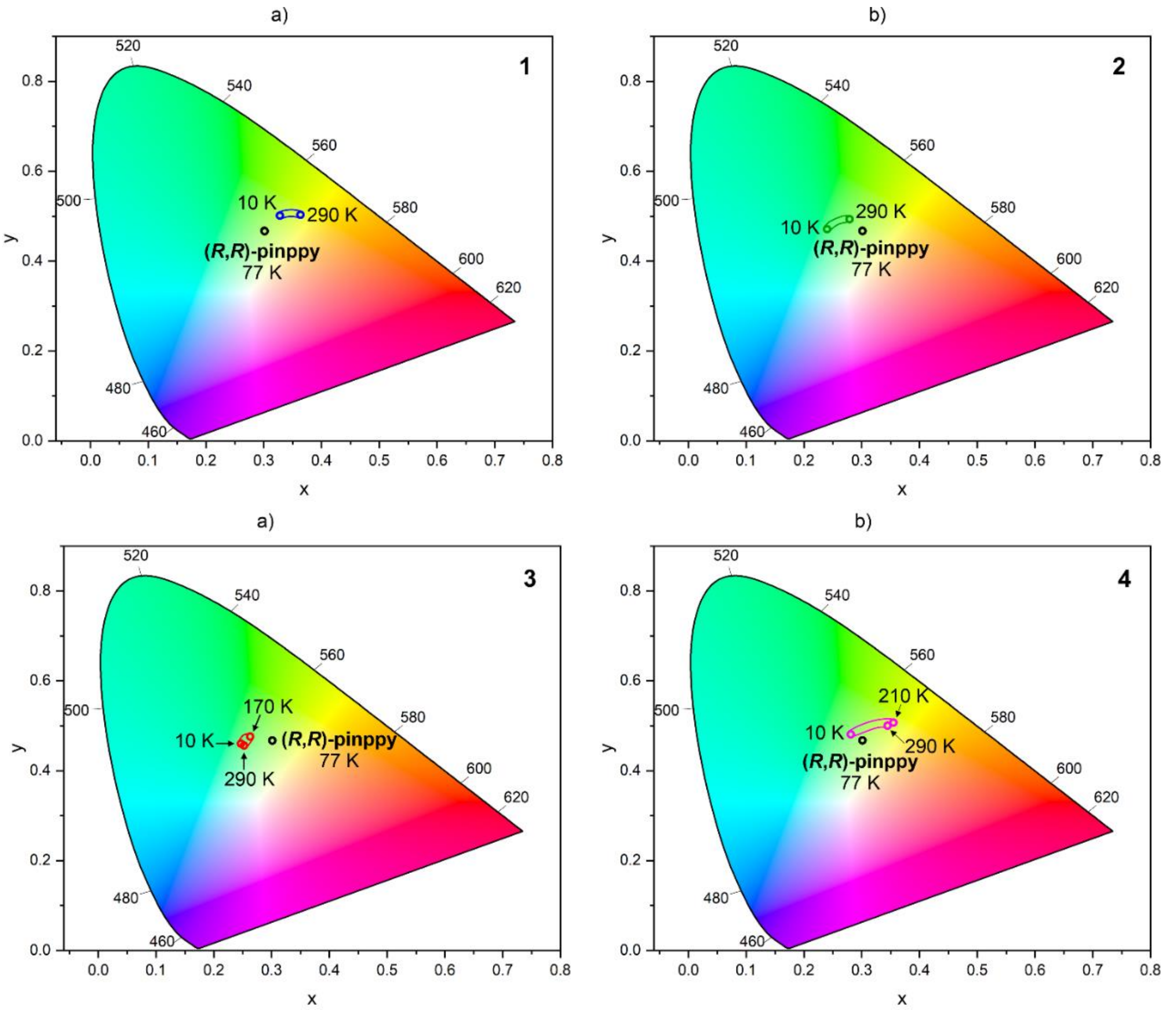

Figure S18. Thermal changes of the emission color in **1**−**4** and **(*R,R*)-pinppy** ligand presented on the CIE 1931 chromaticity diagrams. The related CIE 1931 chromaticity parameters are gathered in Tables S7, S8, S9, and S11, respectively; for **(*R,R*)-pinppy** *x, y* parameters are 0.301, 0.468, respectively.

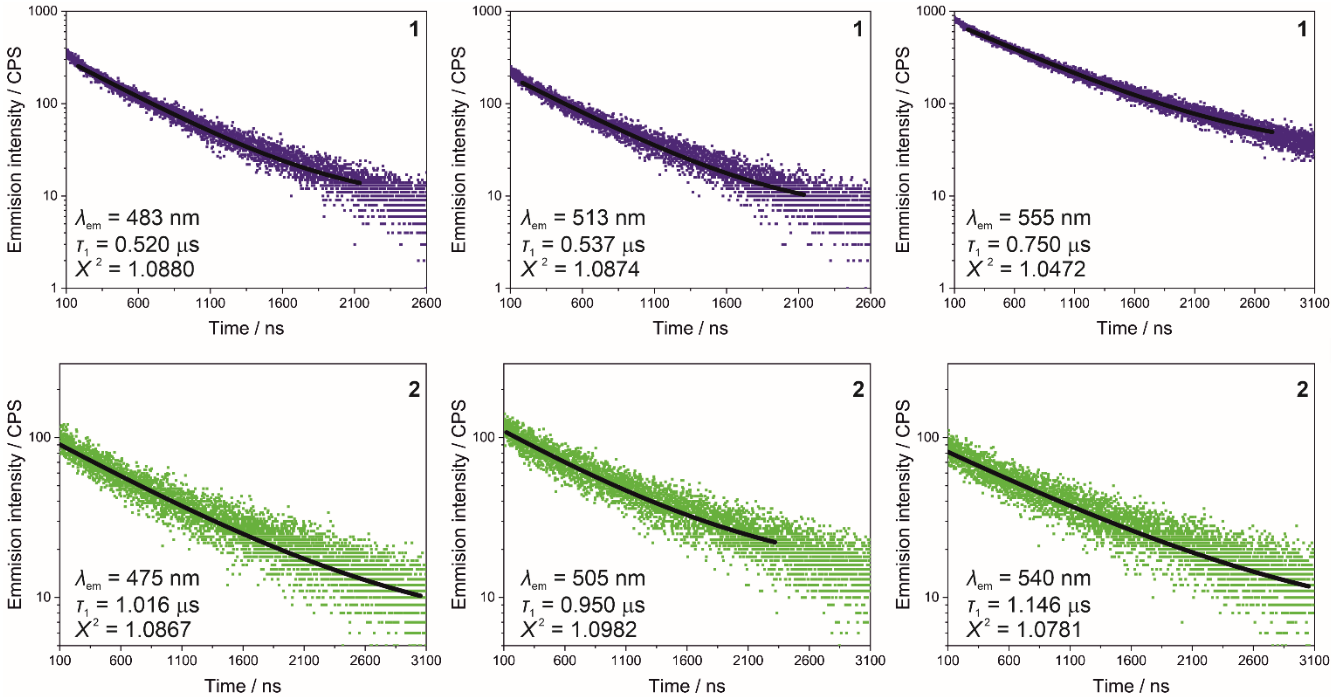

Figure S19. Wavelength-variable emission decay profiles for **1** and **2** under λ_{exc} = 374 nm at room temperature. The mono-exponential fitting was applied for each temperature. The best-fit parameters are roughly presented on the graphs while the detailed values are gathered in Table S12.

Table S12. Best-fit parameters for the room temperature emission decay profiles of **1** and **2** at indicated emission wavelengths.

Compound	$\lambda_{em.}$ / nm	τ (±τ)/ µs	X^2
**1**	483	0.520(±0.003)	1.0880
	513	0.537(±0.004)	1.0874
	555	0.750(±0.003)	1.0472
**2**	475	1.016(±0.012)	1.0876
	505	0.950(±0.018)	1.0982
	540	1.146(±0.017)	1.0781

Comment on the determination of emission lifetimes: It was possible to measure the room-temperature emission lifetimes for three different emission components only for **1** and **2** (Figure S18 and Table S12). For two other compounds, **3** and **4**, only the general emission lifetime for the main emission component was investigated; however, for **3** and **4**, it was possible to gather also the temperature dependences of these emission lifetimes (see Figures S19, S20, and S21, and Table S13).

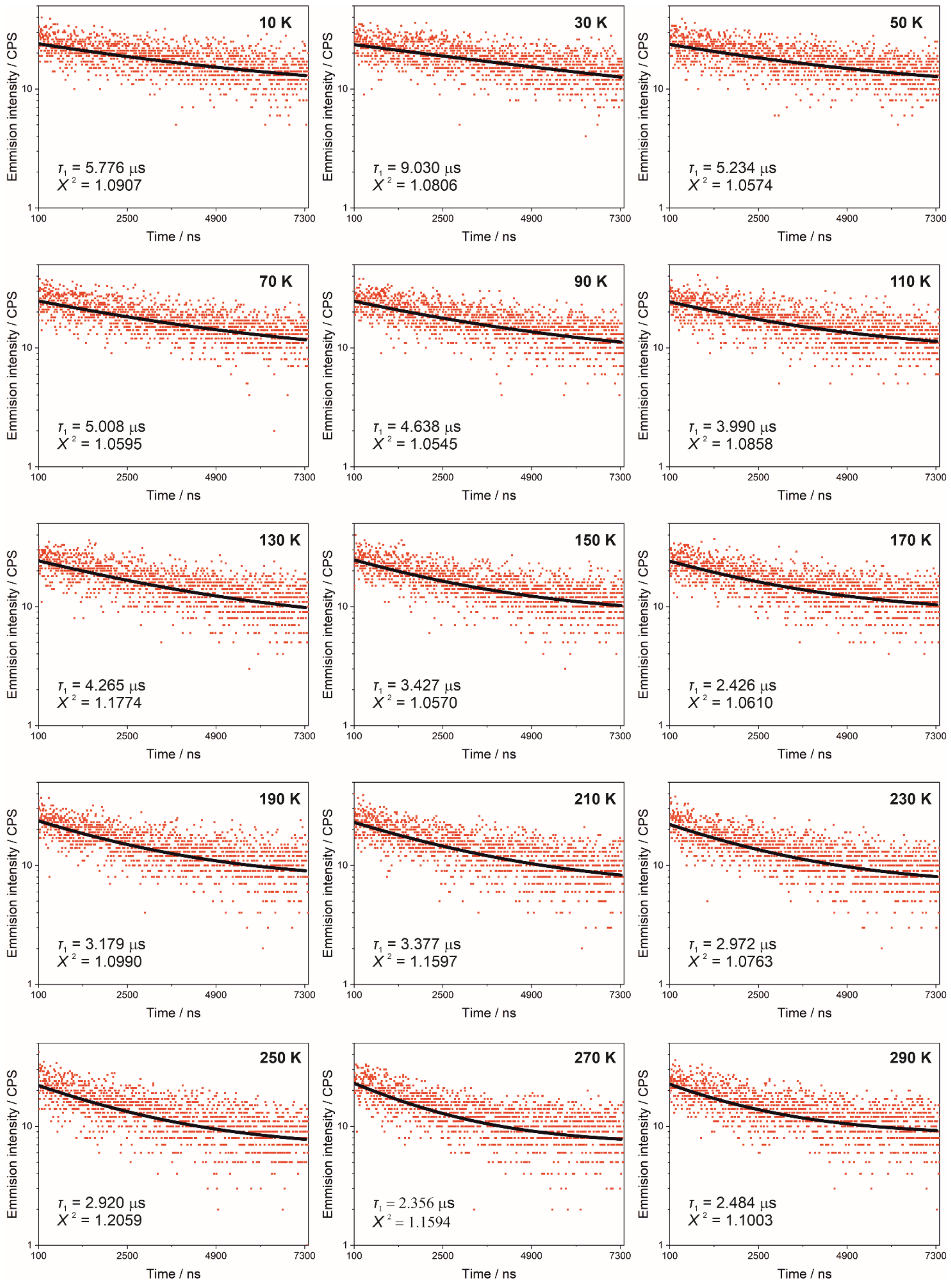

Figure S20. Temperature-variable emission decay profiles for **3** under λ_{exc} = 374 nm and λ_{em} = 500 nm, gathered in the 10–290 K temperature range. The mono-exponential fitting was applied for each temperature. The best-fit parameters are roughly presented on the graphs while the detailed values are gathered in Table S13.

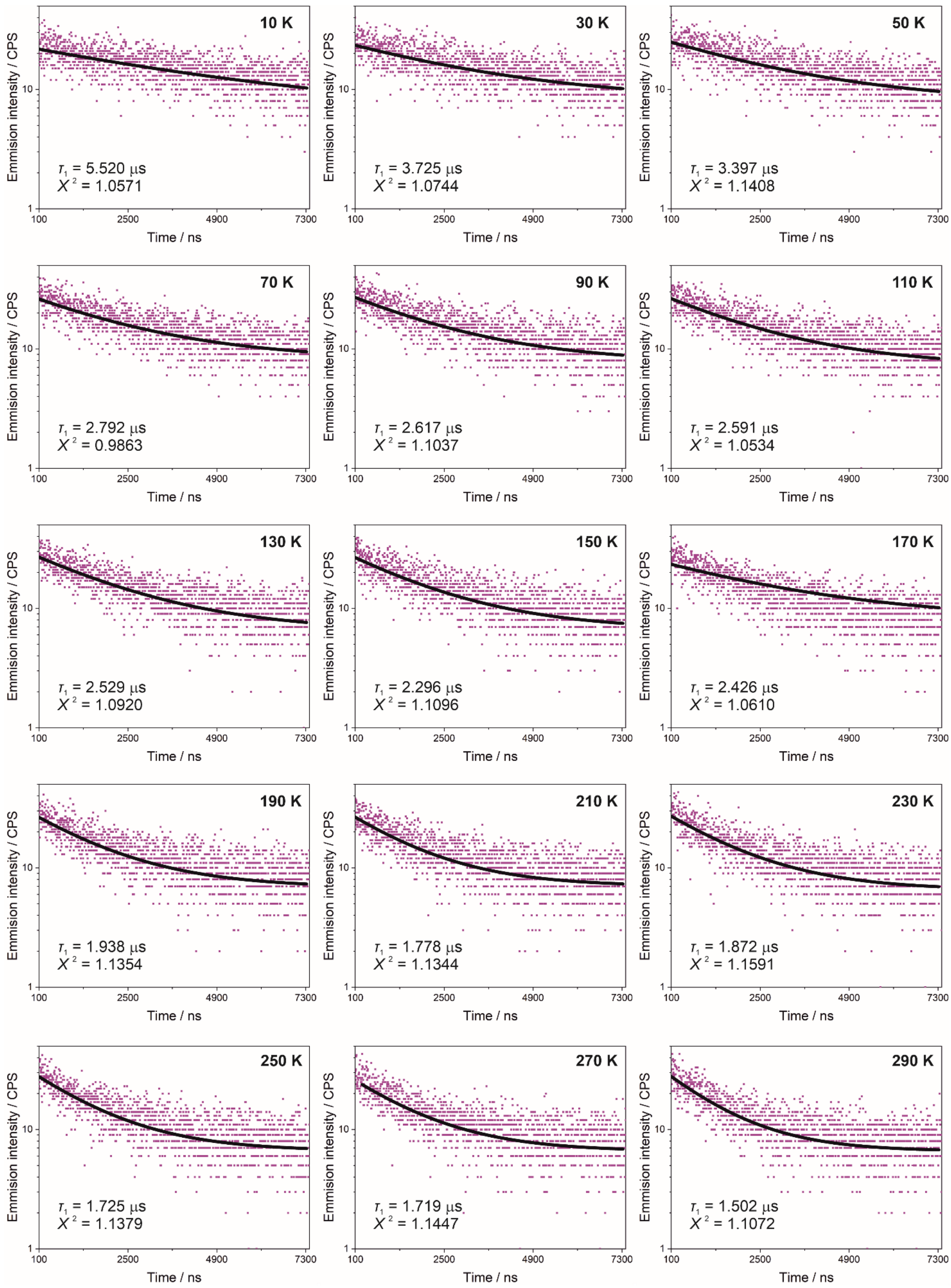

Figure S21. Temperature-variable emission decay profiles for **4** under λ_{exc} = 374 nm and λ_{em} = 510 nm, gathered in the 10–290 K temperature range. The mono-exponential fitting was applied for each temperature. The best-fit parameters are roughly presented on the graphs while the detailed values are gathered in Table S13.

Table S13. Best-fit parameters for the emission decay profiles of **3** and **4** to the mono-exponential decay function for the indicated temperature from the 10–290 K range (Figures S18 and S19).

**3**			**4**		
T / K	τ (±τ)/ μs	X^2	*T* / K	τ (±τ)/ μs	X^2
10	5.776(±1.285)	1.0907	10	5.520(±2.080)	1.0571
30	9.030(±3.034)	1.0806	30	3.725(±0.441)	1.0744
50	5.234(±1.039)	1.0574	50	3.397(±0.324)	1.1408
70	5.008(±0.813)	1.0595	70	2.792(±0.213)	0.9863
90	4.638(±0.673)	1.0545	90	2.617(±0.116)	1.1037
110	3.990(±0.527)	1.0858	110	2.591(±0.170)	1.0534
130	4.265(±0.520)	1.1774	130	2.529(±0.151)	1.0920
150	3.427(±0.352)	1.0570	150	2.296(±0.129)	1.1096
170	3.329(±0.351)	1.0443	170	2.426(±0.142)	1.0610
190	3.179(±0.292)	1.0990	190	1.938(±0.099)	1.1354
210	3.377(±0.326)	1.1597	210	1.778(±0.088)	1.1344
230	2.972(±0.262)	1.0763	230	1.872(±0.090)	1.1591
250	2.920(±0.248)	1.2059	250	1.725(±0.078)	1.1379
270	2.356(±0.164)	1.1594	270	1.719(±0.093)	1.1447
290	2.484(±0.211)	1.1003	290	1.502(±0.064)	1.1072

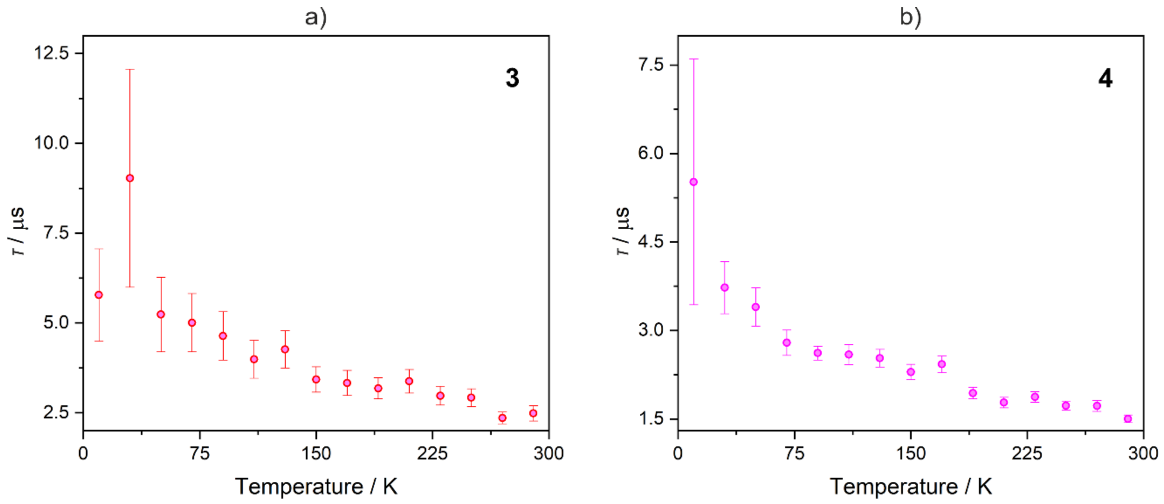

Figure S22. Temperature dependences of emission lifetime for **3** (a) and **4** (b) with the indicated experimental uncertainties.

Computational Details

The theoretical calculations for **1** were carried out using the ORCA 5.0.1 quantum chemistry program package.[S20] In the first step, the geometry of an anionic complex $[Ir^{III}(CN)_2(R,R\text{-pinppy})_2]^-$, consisting of Ir(III) center together with the whole surrounding **(*R*,*R*)-pinppy** ligands and cyanido ligands, taken from the SC-XRD experiment, was optimized using DFT methods. In our treatment, we completely omitted counter ions present in the crystal structure of **1**. The structure was optimized using the B3LYP hybrid exchange-correlation energy functional which presented its reasonable performance for predicting the geometry parameters of ground and excited states,[S21,S22] as well as, excitation energies for various organic and metal transition compounds.[S23,S24] The def2-TZVP basis set was used together with the charge-dependent atom-pairwise dispersion correction using D4(EEQ)-ATM model.[S25,S26] For the calculations, the LR-CPCM solvation model was used with chloroform as a solvent which is consistent with the solvent used for registering the experimental spectra.[S27] The comparison of the SC-XRD structure with the optimized ground state is presented in Figure S23 together with the chosen geometry parameters compared to the experimental ones in Table S14. They show overall good agreement within a deviation of around 0.02 A for the bond lengths and an order of one degree for the angles. The restricted KS determinant of a ground state served then as a reference one for the SOC TD-DFT calculations in the next step. To simulate UV-vis spectra, singlet excited states were optimized using TD-DFT and then mixed with calculated triplet excited states for the optimized ground geometry from the previous step. Scalar relativistic effects were included using the 0th order regular approximation (ZORA)[S28,S29] with a compatible segmented all-electron relativistically contracted basis set SARC-ZORA-TZVP with SARC/J option (general-purpose Coulomb fitting basis set for all-electron calculations which reduces to def2/J for atoms up to Kr and specially implemented auxiliary basis set for atoms beyond Kr, that is Ir in this case).[S30,S31] To accelerate the computation of two-electron integrals, in addition to the resolution of identity approximation for the Coulomb part (RIJ), the chain of spheres algorithm for the exchange part (COSX) was used.[S32,S33] The spin-orbit integrals were calculated using the RI-SOMF(1X) approximation that is: using mean-field potential with the inclusion of 1-electron terms together with Coulomb term computed with RI approximation and exchange terms evaluated via one-center exact integrals including the spin-other orbit interaction omitting DFT local correlation terms.[S34] The maximum number of centers to include in the integrals was set to 4. The list of the first 30 excited singlet states (for the geometry of the ground state optimized in the previous step) is presented in Table S16 together with SOC states obtained by mixing singlets and triplets with the calculated SO-coupling. The theoretical UV-vis spectra from Figure 7 were simulated using the orca_mapspc tool with a broadening of 1800 cm^{-1} for singlets only (TD-DFT) and spin-orbit states (SOC-corrected) compared to the experimental one. The relevant molecular orbitals with the highest contribution to the first five singlet and triplet states (Table S15) are presented in Figure S24. To better understand the mechanism of absorption (and later the emission from those levels) difference density maps for the first three excited SO-states were plotted in Figure S25. After the inspection, it can be seen that the transitions are mainly of metal-to-ligand charge transfer (MLCT) character with a slight admixture of CN^- to ligand CT mechanism. In the last step, to better elucidate the observed luminescence, we simply performed the geometry optimization of the first excited SO-state taking advantage of the possibility to calculate gradients for mixed states in ORCA software. After the optimization, we presented a few first relevant SOC corrected states for the new geometry in Tables S17 and S18 together with the MOs in Figure S26 and geometries compared in Figure S23b analogously to the UV-vis step. One can infer from the density difference maps that the main mechanism of the observed phosphorescence is the MLCT involving **(*R*,*R*)-pinppy** ligands and Ir(III) centers with a slight contribution from the cyanido units and the admixture of intraligand electronic transitions (Figure S27, see the main manuscript for details). The obtained energies of SO-states were compared with emission spectra of **1** (Figures 7 and S28). We did not present relative intensities and therefore lifetimes of the simulated emission bands based on calculated dipole-transition moments, because of the significant impact of vibronic coupling and intersystem crossing rates whose simulations are beyond the scope of this work.

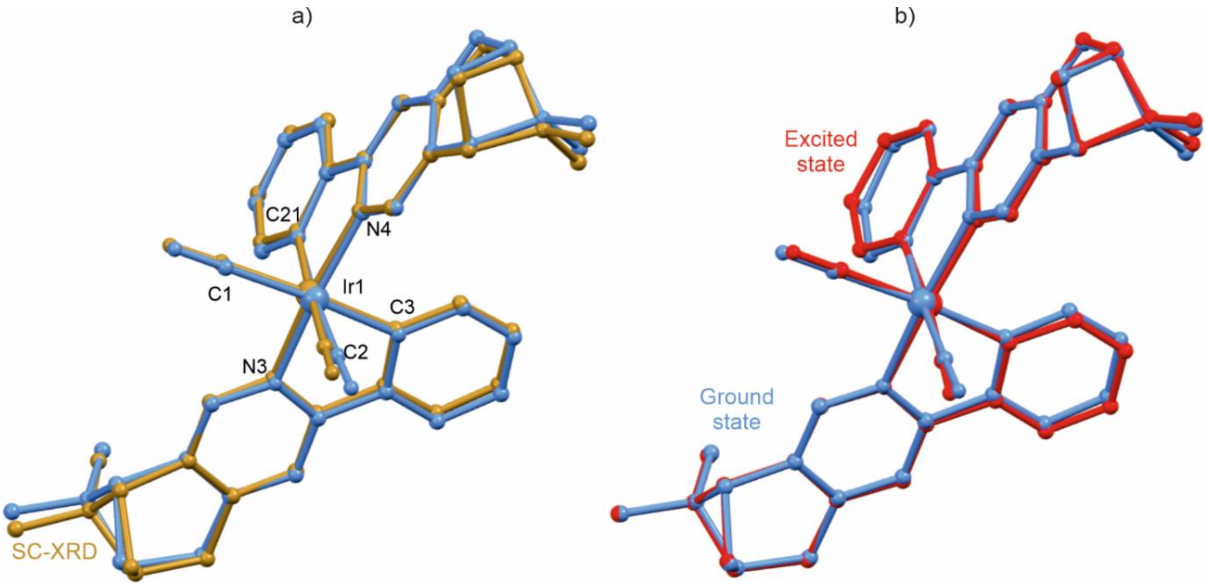

Figure S23. Comparison of the experimental crystal structure (gold, from the SC-XRD experiment, compound **1**) with the optimized ground state (blue) (a) and the comparison of the calculated ground state (blue) with the optimized first excited state (red) (b). Hydrogen atoms were omitted for clarity.

Table S14. The set of representative structural parameters of the octahedral iridium(III) complex from the SC-XRD analysis (compound **1**) with the DFT-optimized geometry of the ground and excited states.

	SC-XRD structure	Optimized ground state	Optimized excited state
Selected bond lengths *cis*-$[Ir^{III}(CN)_2(R,R\text{-pinppy})_2]^-$ complexes / Å,			
Ir1−C1	2.033(5)	2.073	2.112
Ir1−C2	2.044(5)	2.072	2.089
Ir1−C3	2.055(5)	2.061	2.020
Ir1−C21	2.050(5)	2.061	2.053
Ir1−N3	2.055(4)	2.068	2.095
Ir1−N4	2.047(4)	2.072	2.070
Selected angles in *cis*-$[Ir^{III}(CN)_2(R,R\text{-pinppy})_2]^-$ complexes / °			
C1−Ir1−C2	88.1(2)	91.4	84.6
C1−Ir1−C3	174.8(2)	175.4	175.7
C1−Ir1−C21	89.5(2)	91.1	85.93
C1−Ir1−N3	95.72(18)	96.1	96.6
C1−Ir1−N4	92.4(2)	89.9	88.9
C2−Ir1−C3	94.61(19)	90.0	92.2
C2−Ir1−C21	173.6(2)	175.0	170.1
C2−Ir1−N3	90.29(19)	91.1	88.6
C2−Ir1−N4	94.19(18)	96.1	96.0
C3−Ir1−C21	88.2(2)	87.9	97.4
C3−Ir1−N3	79.92(18)	79.5	80.3
C3−Ir1−N4	91.8(2)	94.3	94.4
C21−Ir1−N3	95.87(18)	93.0	95.3
C21−Ir1−N4	79.96(18)	79.5	81.0
N3−Ir1−N4	170.89(17)	170.5	173.1

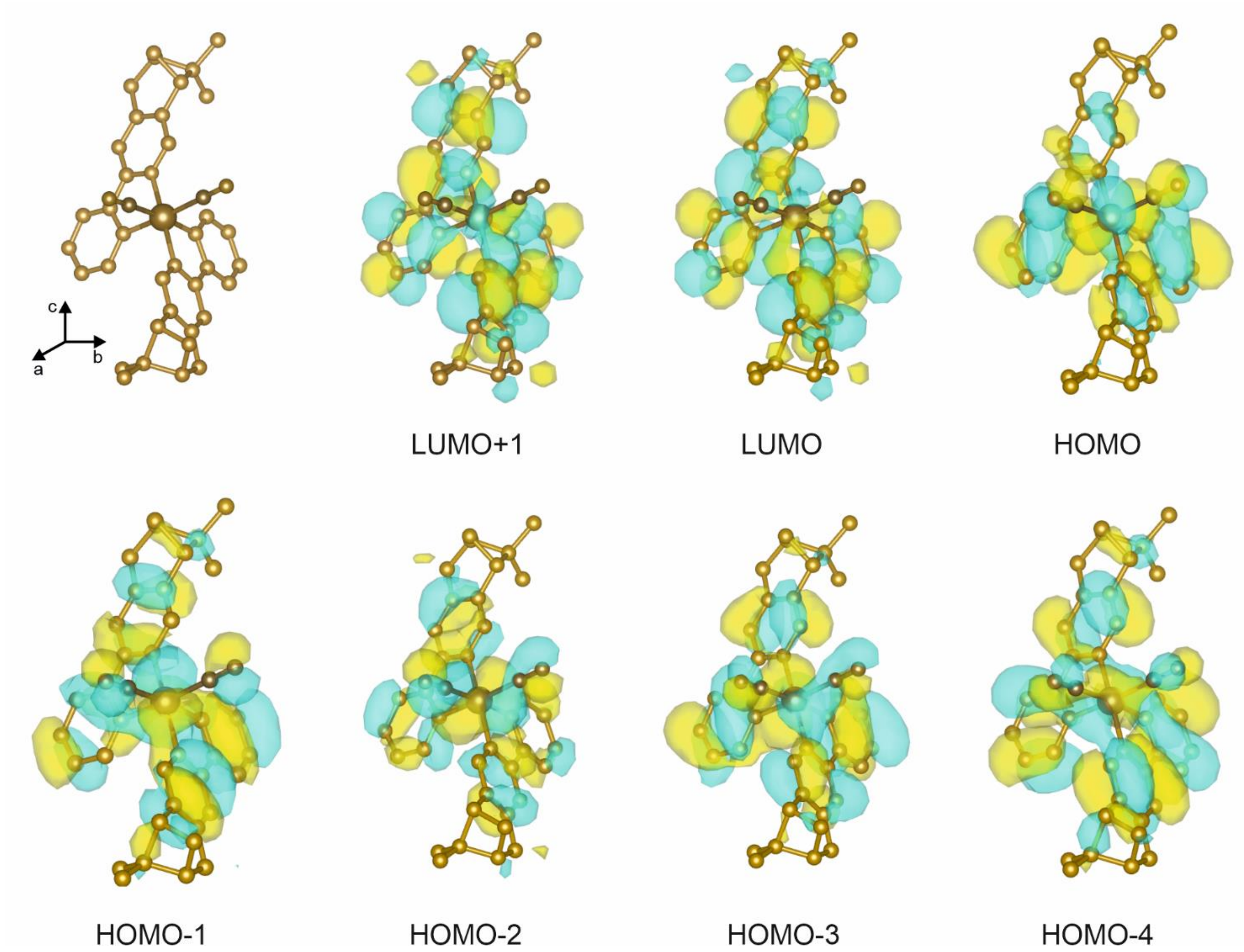

Figure S24. Selected molecular orbitals of the optimized ground state for *cis*-[$Ir^{III}(CN)_2$(*R*,*R*-pinppy)$_2$]$^-$ complexes. Hydrogen atoms were omitted for clarity. Orbitals are plotted with an isosurface level of 0.15.

Table S15. The energies of the first five singlet and triplet states of *cis*-[Ir^{III}(CN)$_2$(*R*,*R*-pinppy)$_2$]$^{-}$ complexes, shown together with the weights of molecular orbitals that contribute to each excitation. For each state factors greater than 0.1 were bolded.

State	Singlets		Triplets	
	Linear combination of molecular orbitals and corresponding weights	Energy / cm^{-1}	Linear combination of molecular orbitals and corresponding weights	Energy / cm^{-1}
1	**HOMO→LUMO (0.978)**	25834.3	HOMO−4→LUMO (0.061)<br>HOMO−4→LUMO+2 (0.013)<br>HOMO−3→LUMO+1 (0.031)<br>HOMO−3→LUMO+3 (0.019)<br>HOMO−2→LUMO (0.049)<br>**HOMO−1→LUMO+1 (0.198)**<br>**HOMO→LUMO (0.533)**	22666.1
2	**HOMO→LUMO+1 (0.974)**	26395.0	HOMO−5→LUMO (0.016)<br>HOMO−4→LUMO+1 (0.049)<br>HOMO−4→LUMO+3 (0.021)<br>HOMO−3→LUMO (0.050)<br>HOMO−3→LUMO+2 (0.013)<br>HOMO−2→LUMO+1 (0.054)<br>**HOMO−1→LUMO (0.275)**<br>**HOMO→LUMO+1 (0.421)**<br>HOMO→LUMO+3 (0.015)	22826.9
3	HOMO−5→LUMO (0.010)<br>HOMO−3→LUMO (0.083)<br>HOMO−2→LUMO+1 (0.098)<br>**HOMO−1→LUMO (0.790)**	29402.8	HOMO−5→LUMO+1 (0.017)<br>HOMO−5→LUMO+3 (0.0160)<br>HOMO−4→LUMO (0.054)<br>HOMO−4→LUMO+2 (0.017)<br>HOMO−3→LUMO+1 (0.022)<br>**HOMO−2→LUMO (0.182)**<br>HOMO−2→LUMO+1 (0.017)<br>HOMO−1→LUMO (0.024)<br>HOMO−1→LUMO+1 (0.174)<br>**HOMO→LUMO (0.321)**<br>HOMO→LUMO+1 (0.058)	25979.7
4	HOMO−3→LUMO+1 (0.027)<br>**HOMO−2→LUMO (0.457)**<br>**HOMO−1→LUMO+1 (0.322)**<br>**HOMO→LUMO+2 (0.169)**	29484.7	HOMO−5→LUMO (0.021)<br>HOMO−5→LUMO+2 (0.017)<br>HOMO−4→LUMO+1 (0.019)<br>HOMO−4→LUMO+3 (0.020)<br>HOMO−3→LUMO (0.025)<br>HOMO−3→LUMO+2 (0.016)<br>HOMO−2→LUMO (0.032)<br>**HOMO−2→LUMO+1 (0.158)**<br>**HOMO−1→LUMO (0.162)**<br>HOMO−1→LUMO+1 (0.022)<br>HOMO→LUMO (0.045)<br>**HOMO→LUMO+1 (0.386)**	26093.0
5	HOMO−3→LUMO+1 (0.046)<br>HOMO−2→LUMO (0.015)<br>**HOMO−1→LUMO+1 (0.172)**<br>**HOMO→LUMO+2 (0.745)**	29609.4	HOMO−5→LUMO+3 (0.011)<br>HOMO−4→LUMO+2 (0.044)<br>HOMO−3→LUMO+3 (0.058)<br>HOMO−2→LUMO (0.033)<br>HOMO−1→LUMO+1 (0.011)<br>HOMO−1→LUMO+3 (0.065)<br>**HOMO→LUMO+2 (0.704)**	27721.3

Table S16. The energies of singlet states and SO-states for the ground geometry of *cis*-[Ir^{III}(CN)$_2$(*R*,*R*-pinppy)$_2$]$^-$ complexes, and the composition of SO-states in terms of singlet and triplet states for the first thirty states.

TD-DFT (singlets)		SOC corrected TD-DFT (mixed)								
State	Energy / cm^{-1}	State	Energy / cm^{-1}	Spin	State	Energy / cm^{-1}	State	Energy / cm^{-1}	State	Energy / cm^{-1}
1	25834.3	1	23004.4	1 (91%)	31	30223.4	61	33485.1	91	36257.3
2	26395.0	2	23033.6	1 (93%)	32	30301.9	62	34057.1	92	36451.1
3	29402.8	3	23046.8	0 (2%)<br>1 (96%)	33	30314.7	63	34081.1	93	36526.7
4	29484.7	4	23202.7	1 (91%)	34	30503.2	64	34166.1	94	36566.3
5	29609.4	5	23220.4	1 (91%)	35	30540.5	65	34343.7	95	36610.5
6	30129.2	6	23250.4	1 (94%)	36	30786.0	66	34396.5	96	36678.3
7	30695.3	7	25145.5	0 (50%)<br>1 (41%)	37	30965.7	67	34971.5	97	36799.8
8	31197.7	8	25351.1	0 (39%)<br>1 (50%)	38	30974.2	68	35011.2	98	36819.1
9	31829.3	9	26105.7	0 (2%)<br>1 (89%)	39	31174.8	69	35041.4	99	36877.7
10	32216.5	10	26109.9	0 (3%)<br>1 (85%)	40	31180.5	70	35070.0	100	36918.7
11	33259.9	11	26170.5	1 (91%)	41	31276.9	71	35100.9	101	37084.2
12	33443.4	12	26187.7	0 (5%)<br>1 (87%)	42	31304.8	72	35346.3	102	37304.6
13	34087.1	13	26727.8	0 (30%)<br>1 (63%)	43	31587.2	73	35384.9	103	37436.6
14	34498.9	14	26993.5	0 (31%)<br>1 (59%)	44	31699.1	74	35414.8	104	37443.4
15	35034.4	15	27918.5	1 (89%)	45	31824.6	75	35427.0	105	37498.4
16	35340.5	16	27933.3	0 (4%)<br>1 (87%)	46	31910.7	76	35435.6	106	37510.7
17	35438.0	17	27977.8	0 (2%)<br>1 (88%)	47	31965.8	77	35558.0	107	37648.9
18	35690.0	18	28269.5	0 (19%)<br>1 (64%)	48	32018.9	78	35562.3	108	37727.1
19	35741.2	19	28477.7	0 (17%)<br>1 (77%)	49	32044.7	79	35631.5	109	37732.0
20	36145.5	20	28560	1 (94%)	50	32074.5	80	35649.9	110	37789.2
21	36597.1	21	28672.5	0 (8%)<br>1 (82%)	51	32618.6	81	35703.6	111	38663.9
22	36855.4	22	28734.3	0 (10%)<br>1 (78%)	52	32697.7	82	35755.7	112	39257.7
23	37922.3	23	28775.4	0 (1%)<br>1 (90%)	53	32959.6	83	35963.3	113	39294.2
24	38483.2	24	29057.2	0 (3%)<br>1 (85%)	54	33032.8	84	36042.5	114	39550.8
25	38803.5	25	29090.8	0 (3%)<br>1 (85%)	55	33096.1	85	36056.1	115	39699.1
26	39325.4	26	29094.7	1 (85%)	56	33122.2	86	36070.0	116	40130.3
27	39428.0	27	29234.7	0 (47%)<br>1 (48%)	57	33199.1	87	36090.5	117	40162.8
28	39712.6	28	29699.9	0 (13%)<br>1 (71%)	58	33278.5	88	36141.3	118	40382.2
29	39813.4	29	29723.0	0 (27%)<br>1 (56%)	59	33389.2	89	36189.2	119	40500.4
30	39843.1	30	30130.1	1 (90%)	60	33405.5	90	36198.0	120	41321.6

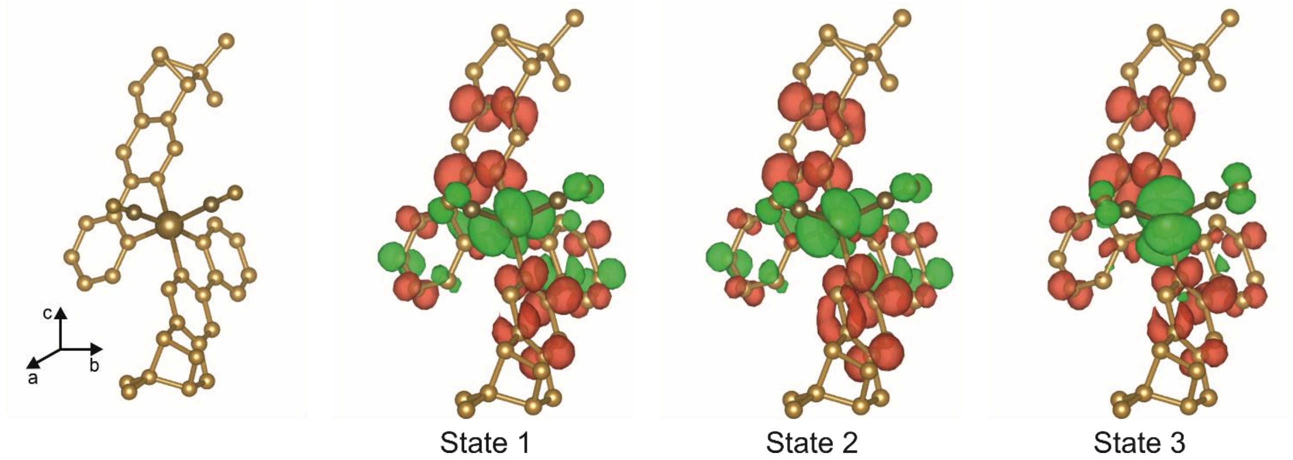

Figure S25. Difference density maps of three lowest SO-states (SOC corrected TD-DFT states, see Table S16) for the ground state geometry of *cis*-$[Ir^{III}(CN)_2(R,R\text{-pinppy})_2]^-$ complexes. Red color represents the positive (build-up) change in electron density, green represents the negative change (outflow) of electron density. Hydrogen atoms were omitted for clarity. The densities were plotted with an isosurface level of 0.0016.

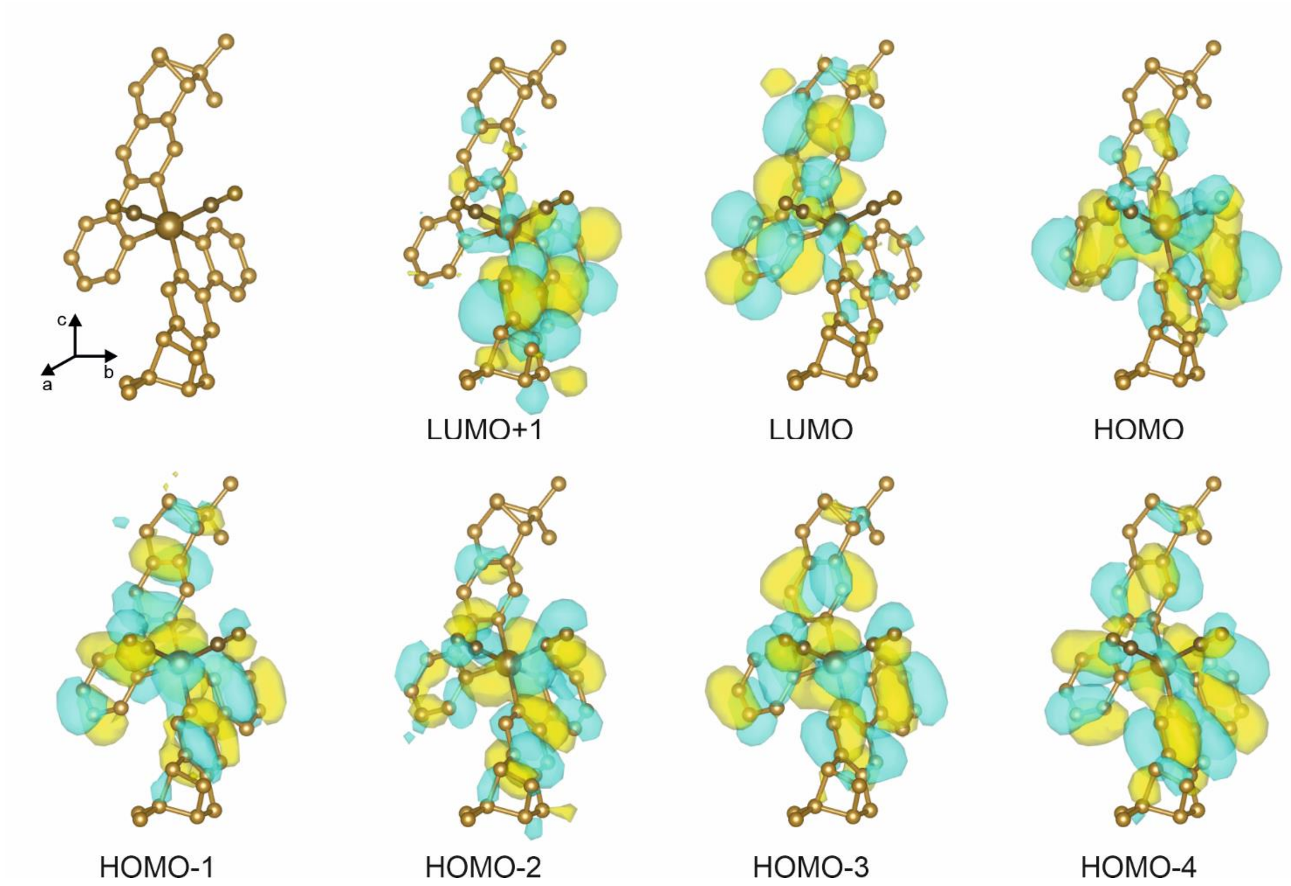

Figure S26. Selected molecular orbitals for the optimized geometry of the first excited SO-state of *cis*-$[Ir^{III}(CN)_2$(*R*,*R*-pinppy)$_2]^-$ complexes. Hydrogen atoms were omitted for clarity. Orbitals are plotted with an isosurface level of 0.15.

Table S17. The energies of the first seven singlet and triplet states for the optimized geometry of the first excited SO-state of *cis*-$[Ir^{III}(CN)_2(R,R\text{-pinppy})_2]^-$ complexes, together with the weights of molecular orbitals contributing to each excitation. For each state factors greater than 0.1 were bolded.

State	Singlets		Triplets	
	Linear combination of molecular orbitals and corresponding weights	Energy / cm^{-1}	Linear combination of molecular orbitals and corresponding weights	Energy / cm^{-1}
1	**HOMO→LUMO (0.987)**	21788.4	HOMO−4→LUMO (0.015)<br>HOMO−3→LUMO (0.034)<br>HOMO−2→LUMO (0.010)<br>**HOMO−1→LUMO (0.191)**<br>**HOMO→LUMO (0.687)**	19381.7
2	**HOMO→LUMO+1 (0.982)**	24178.8	HOMO−4→LUMO+1 (0.021)<br>HOMO−3→LUMO+1 (0.021)<br>HOMO−2→LUMO+1 (0.049)<br>HOMO−1→LUMO (0.076)<br>HOMO−1→LUMO+1 (0.041)<br>HOMO→LUMO (0.040)<br>**HOMO→LUMO+1 (0.673)**	21604.2
3	HOMO−3→LUMO (0.066)<br>HOMO−2→LUMO (0.094)<br>**HOMO−1→LUMO (0.796)**<br>HOMO→LUMO+2 (0.011)	27395.9	HOMO−5→LUMO (0.027)<br>HOMO−4→LUMO (0.040)<br>HOMO−2→LUMO (0.092)<br>**HOMO−1→LUMO (0.412)**<br>HOMO−1→LUMO+1 (0.024)<br>**HOMO→LUMO (0.225)**<br>**HOMO→LUMO+1 (0.102)**	22481.3
4	**HOMO→LUMO+2 (0.974)**	27581.7	HOMO−5→LUMO+1 (0.016)<br>HOMO−4→LUMO+1 (0.067)<br>HOMO−4→LUMO+2 (0.030)<br>HOMO−4→LUMO+3 (0.018)<br>HOMO−3→LUMO+2 (0.018)<br>HOMO−2→LUMO (0.040)<br>**HOMO−2→LUMO+1 (0.306)**<br>**HOMO−1→LUMO+1 (0.143)**<br>**HOMO→LUMO+1 (0.175)**<br>HOMO→LUMO+2 (0.036)<br>HOMO→LUMO+3 (0.017)	24744.1
5	HOMO−4→LUMO (0.029)<br>**HOMO−2→LUMO (0.827)**<br>**HOMO−1→LUMO (0.107)**	28064.6	HOMO−5→LUMO+1 (0.011)<br>HOMO−4→LUMO+1 (0.015)<br>HOMO−3→LUMO+3 (0.022)<br>HOMO−2→LUMO (0.018)<br>HOMO−2→LUMO+1 (0.022)<br>HOMO−1→LUMO+1 (0.012)<br>HOMO−1→LUMO+3 (0.018)<br>**HOMO→LUMO+2 (0.818)**	26006.9

Table S18. The energies of the first five singlet states and SO-states for the optimized geometry of the first excited SO-state together with the composition of SO-states in terms of singlet and triplet states.

TD-DFT		SOC corrected TD-DFT								
State	Energy / cm^{-1}	State	Energy / cm^{-1}	Spin	State	Energy / cm^{-1}	Spin	State	Energy / cm^{-1}	Spin
1	21788.4	1	19196.3	1 (98%)	8	22481.0	1 (98%)	15	26022.6	1 (99%)
2	24178.8	2	19304.2	1 (100%)	9	22567.6	1 (99%)	16	26027.0	1 (99%)
3	27395.9	3	19315.3	1 (100%)	10	22746.0	0 (26%)<br>1 (72%)	17	26034.6	1 (98%)
4	27581.7	4	21337.1	0 (47%)<br>1 (53%)	11	23858.9	0 (71%)<br>1 (28%)	18	27583.4	0 (99%)
5	28064.6	5	21550.8	1 (97%)	12	24798.0	1 (97%)	19	27629.6	0 (97%)
-	-	6	21585.9	0 (3%)<br>1 (94%)	13	24809.1	1 (98%)	20	28210.9	0 (98%)<br>1 (1%)
-	-	7	21713.1	0 (24%)<br>1 (75%)	14	25106.7	0 (29%)<br>1 (71%)	-	-	-

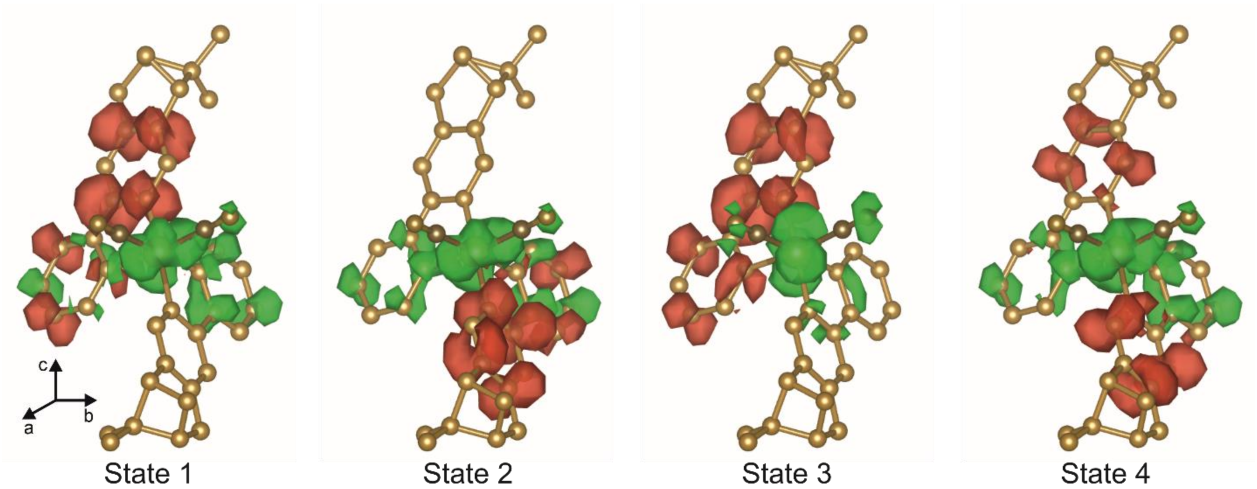

Figure S27. Density difference maps for the four lowest energy SOC corrected TD-DFT states in *cis*-$[Ir^{III}(CN)_2$(*R*,*R*-pinppy)$_2]^-$ complexes (for the optimized geometry of the first excited SO-state, see Table S18 for comparison). The red color represents a positive (build-up) change in electron density, the green represents a negative change (outflow) of electron density. Hydrogen atoms were omitted for clarity. The densities were plotted with an isosurface level of 0.0016.

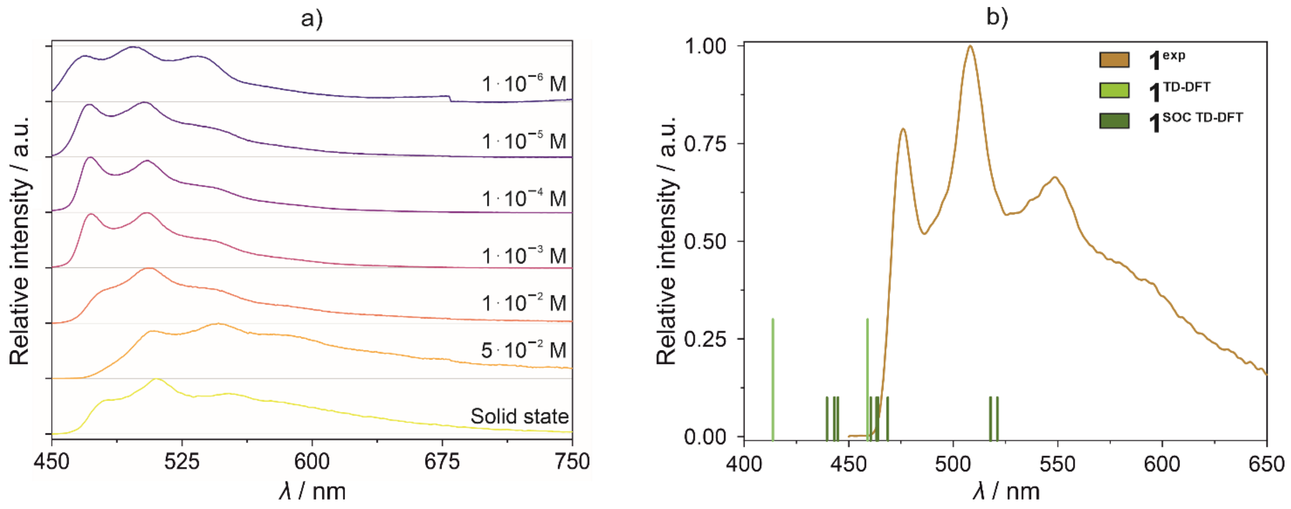

Figure S28. Room-temperature emission spectra of *cis*-$[Ir^{III}(CN)_2(R,R\text{-pinppy})_2]^-$ complexes of **1** dissolved in variable indicated concentrations in $CHCl_3$ (a), and the comparison of the solid-state emission spectrum of **1** at 10K together with the TD-DFT and SOC corrected TD-DFT calculation of excited states emission.

References to the Supporting Information

S1 H.-X. Liu, H.-B. Tan, M.-T. He, L. Li, Y.-H. Wang and C.-L. Long, Isolation and synthesis of two hydroxychavicol heterodimers from Piper nudibaccatum, *Tetrahedron*, 2015, **71**, 2369−2375.

S2 J. Brioche, C. Meyer and J. Cossy, Synthesis of 2-Aminoindolizines by 1,3-Dipolar Cycloaddition of Pyridinium Ylides with Electron-Deficient Ynamides, *Org. Lett.*, 2015, **17**, 2800−2803.

S3 Y. Fukazawa, V.Y. Vaganov, S. A. Shiplilovskikh, A. E. Rubtsov and A. V. Malkov, Stereoselective Synthesis of Atropisomeric Bipyridine N,N′-Dioxides by Oxidative Coupling, *Org. Lett*. 2019, **21**, 4798−4802.

S4 A. Kurutos, I. Orehovec, D. Saftić, L. Horvat, I. Crnolatac, I. Piantanida and T. Deligeorgiev, Cell penetrating, mitochondria targeting multiply charged DABCO-cyanine dyes, *Dyes Pigm.*, 2018, **158**, 517–525.

S5 M. K. Nazeeruddin, R. Humphry-Baker, D. Berner, S. Rivier, L. Zuppiroli and M. Graetzel, Highly Phosphorescence Iridium Complexes and Their Application in Organic Light-Emitting Devices, *J. Am. Chem. Soc.,* 2003, **125**, 8790−8797.

S6 B. F. Minaev, V. A. Minaeva, G. V. Baryshnikov, M. A. Girtu and H. Agren, Theoretical study of vibration spectra of sensitizing dyes for photoelectrical converters based on ruthenium(II) and iridium(III) complexes, *Russ. J. Appl. Chem.,* 2009, **82**, 1211–1221.

S7 G. M. Sheldrick, SHELXT - Integrated space-group and crystal-structure determination, *Acta Crystallogr., Sect. A: Found. Adv.,* 2015, **71**, 3–8.

S8 L. J. Farrugia, WinGX and ORTEP for Windows: an update, *J. Appl. Crystallogr.,* 2012, **45**, 849–854.

S9 R. Jankowski, J. J. Zakrzewski, M. Zychowicz, J. Wang, Y. Oki, S. Ohkoshi, S. Chorazy and B. Sieklucka, SHG-active NIR-emissive molecular nanomagnets generated in layered neodymium(III)–octacyanidometallate(IV) frameworks, *J. Mater. Chem. C*, 2021, **9**, 10705−10717.

S10 S. Chorazy, J. J. Zakrzewski, J. Wang, S. Ohkoshi and B. Sieklucka, Incorporation of hexacyanidoferrate(III) ion in photoluminescent trimetallic Eu(3-pyridone)[$Co_{1-x}Fe_x(CN)_6$] chains exhibiting tunable visible light absorption and emission properties, *CrystEngComm*, 2018, **20**, 5695–5706.

S11 M. Llunell, D. Casanova, J. Cirera, J. Bofill, P. Alemany, S. Alvarez, M. Pinsky and D. Avnir, *SHAPE v. 2.1. Program for the Calculation of Continuous Shape Measures of Polygonal and Polyhedral Molecular Fragments*, University of Barcelona: Barcelona, Spain, 2013.

S12 D. Casanova, J. Cirera, M. Llunell, P. Alemany, D. Avnir and S. Alvarez, Minimal Distortion Pathways in Polyhedral Rearrangements, *J. Am. Chem. Soc.*, 2004, **126**, 1755–1763.

S13 A. Ruiz-Martínez, D. Casanova and S. Alvarez, Polyhedral Structures with an Odd Number of Vertices: Nine-Coordinate Metal Compounds, *Chem. Eur. J.,* 2008, **14**, 1291–1303.

S14 D. A. Kleinman, Theory of Second Harmonic Generation of Light, *Phys. Rev.*, 1962, **128**, 1761–1775.

S15 D. A. Kleinman, Nonlinear Dielectric Polarization in Optical Media, *Phys. Rev.*, 1977, **126**, 1977–1979.

S16 G. R. Crane, J. G. Bergman, Violations of Kleinman symmetry in nonlinear optics: The forbidden coefficient of α-quartz, *J. Chem. Phys.*, 1976, **64**, 27–29.

S17 C. A. Dailey, B. J. Burke and G. J. Simpson, The general failure of Kleinman symmetry in practical nonlinear optical applications, *Chem. Phys. Lett.*, 2004, **390**, 8–13.

S18 H.-J. Zhao, Y.-F. Zhang and L. Chen, *J. Am. Chem. Soc.*, 2012, **134**, 1993–1995.

S19 J. Wang, J. J. Zakrzewski, M. Zychowicz, Y. Xin, H. Tokoro, S. Chorazy and S. Ohkoshi, Desolvation-Induced Highly Symmetrical Terbium(III) Single-Molecule Magnet Exhibiting Luminescent Self-Monitoring of Temperature, *Angew. Chem. Int. Ed*., 2023, **62**, e202306372.

S20 F. Neese, F. Wennmohs, U. Becker and C. Riplinger, The ORCA quantum chemistry program package, *J. Chem. Phys.*, 2020, **152**, 224108.

S21 A. D. Becke, Density-functional exchange-energy approximation with correct asymptotic behavior, *Phys. Rev. A*, 1988, **38**, 3098–3100.

S22 C. Lee, W. Yang and R. G. Parr, Development of the Colle-Salvetti correlation-energy formula into a functional of the electron density, *Phys. Rev. B*, 1988, **37**, 785–789.

S23 B. de Souza, G. Farias, F. Neese and R. Izsák, Predicting Phosphorescence Rates of Light Organic Molecules Using Time-Dependent Density Functional Theory and the Path Integral Approach to Dynamics, *J. Chem. Theory Comput.*, 2019, **15**, 1896–1904.

S24 C. Latouche, D. Skouteris, F. Palazzetti and V. Barone, TD-DFT Benchmark on Inorganic Pt(II) and Ir(III) Complexes, *J. Chem. Theory Comput.*, 2015, **11**, 3281–3289.

S25 F. Weigend and R. Ahlrichs, Balanced basis sets of split valence, triple zeta valence and quadruple zeta valence quality for H to Rn: Design and assessment of accuracy, *Phys. Chem. Chem. Phys.*, 2005, **7**, 3297–3305.

S26 E. Caldeweyher, S. Ehlert, A. Hansen, H. Neugebauer, S. Spicher, C. Bannwarth and S. Grimme, A generally applicable atomic-charge dependent London dispersion correction, *J. Chem. Phys.*, 2019, **150**, 154122.

S27 R. Cammi, B. Mennucci and J. Tomasi, Fast Evaluation of Geometries and Properties of Excited Molecules in Solution: A Tamm-Dancoff Model with Application to 4-Dimethylaminobenzonitrile, *J. Phys. Chem. A*, 2000, **104**, 5631–5637.

S28 E. van Lenthe, E. J. Baerends and J. G. Snijders, Relativistic regular two-component Hamiltonians, *J. Chem. Phys.*, 1993, **99**, 4597–4610.

S29 C. van Wüllen, Molecular density functional calculations in the regular relativistic approximation: Method, application to coinage metal diatomics, hydrides, fluorides and chlorides, and comparison with first-order relativistic calculations, *J. Chem. Phys.*, 1998, **109**, 392–399.

S30 D. A. Pantazis, X.-Y. Chen, C. R. Landis and F. J. Neese, All-Electron Scalar Relativistic Basis Sets for Third-Row Transition Metal Atoms, *J. Chem. Theory Comput.*, 2008, **4**, 908–919.

S31 M. Buhl, C. Reimann, D. A. Pantazis, T. Bredow and F. Neese, Geometries of Third-Row Transition-Metal Complexes from Density-Functional Theory, *J. Chem. Theory Comput.*, 2008, **4**, 1449–1459.

S32 F. Neese, F. Wennmohs, A. Hansen and U. Becker, Efficient, approximate and parallel Hartree-Fock and hybrid DFT calculations. A ‘chain-of-spheres’ algorithm for the Hartree-Fock exchange, *Chem. Phys.*, 2009, **356**, 98–109.

S33 R. Izsák and F. Neese, An overlap fitted chain of spheres exchange method, *J. Chem. Phys.*, 2011, **135**, 144105.

S34 F. Neese, Efficient and accurate approximations to the molecular spin-orbit coupling operator and their use in molecular g-tensor calculations, *J. Chem. Phys.*, 2005, **122**, 034107.
